# Integrative proteomic and glycoproteomic profiling of *Mycobacterium tuberculosis* culture filtrate

**DOI:** 10.1371/journal.pone.0221837

**Published:** 2020-03-03

**Authors:** Paula Tucci, Madelón Portela, Carlos Rivas Chetto, Gualberto González-Sapienza, Mónica Marín

**Affiliations:** 1 Sección Bioquímica, Facultad de Ciencias, Universidad de la República, Montevideo, Uruguay; 2 Unidad de Bioquímica y Proteómica Analíticas, Institut Pasteur de Montevideo, Montevideo, Uruguay; 3 Facultad de Ciencias, Universidad de la República, Montevideo, Uruguay; 4 Departamento de Laboratorio, Comisión Honoraria para la Lucha Antituberculosa y Enfermedades Prevalentes, Centro de Referencia Nacional para Micobacterias, Ministerio de Salud Pública, Montevideo, Uruguay; 5 Cátedra de Inmunología, DEPBIO, Facultad de Química, Universidad de la Republica Uruguay, Montevideo, Uruguay; Institut de Pharmacologie et de Biologie Structurale, FRANCE

## Abstract

Despite being the subject of intensive research, tuberculosis, caused by *Mycobacterium tuberculosis*, remains at present the leading cause of death from an infectious agent. Secreted and cell wall proteins interact with the host and play important roles in pathogenicity. These proteins are explored as candidate diagnostic markers, potential drug targets or vaccine antigens, and more recently special attention is being given to the role of their post-translational modifications. With the purpose of contributing to the proteomic and glycoproteomic characterization of this important pathogen, we performed a shotgun analysis of culture filtrate proteins of *M*. *tuberculosis* based on a liquid nano-HPLC tandem mass spectrometry and a label-free spectral counting normalization approach for protein quantification. We identified 1314 *M*. *tuberculosis* proteins in culture filtrate and found that the most abundant proteins belong to the extracellular region or cell wall compartment, and that the functional categories with higher protein abundance factor were virulence, detoxification and adaptation, and cell wall and cell processes. We could identify a group of proteins consistently detected in previous studies, most of which were highly abundant proteins. In culture filtrate, 140 proteins were predicted to contain one of the three types of bacterial N-terminal signal peptides. Besides, various proteins belonging to the ESX secretion systems, and to the PE and PPE families, secreted by the type VII secretion system using nonclassical secretion signals, were also identified. O-glycosylation was identified in 46 proteins, many of them lipoproteins and cell wall associated proteins. Finally, we provide proteomic evidence for 33 novel O-glycosylated proteins, aiding to the glycoproteomic characterization of relevant antigenic membrane and exported proteins. These findings are expected to collaborate with the research on pathogen derived biomarkers, virulence factors and vaccine candidates, and to provide clues to the understanding of the pathogenesis and survival strategies adopted by *M*. *tuberculosis*.

## Introduction

*Mycobacterium tuberculosis*, the causative agent of tuberculosis (TB) remains a major public health threat. According to the last Global Tuberculosis Report published by the World Health Organization (WHO) an estimate of 10 million people developed TB disease in 2018. Moreover, TB is at present the leading cause of death from a single infectious agent, causing an estimated 1.2 million deaths among HIV-negative people and approximately 250 thousand deaths among HIV-positive people [1]. Although TB diagnosis and successful treatment averts millions of deaths each year, there are still large and persistent gaps related to this infection that must be resolved in order to accelerate progress towards the goal of ending the TB epidemic endorsed by WHO [1].

*M*. *tuberculosis* (MTB) has evolved successful mechanisms to circumvent the hostile environment of the macrophage, such as inhibiting the phagosome-lysosome fusion and to escape the acidic environment inside the phagolysosome [2]. MTB may be unique in its ability to exploit adaptive immune responses, through inflammatory lung tissue damage, to promote its transmission [3]. It has been proposed that this microorganism was pressed by an evolutionary selection that resulted in an infection that induces partial immunity, where the host survives a long period after being infected with the pathogen, aiding in microorganism persistence and transmission [3]. MTB mechanisms of evasion of host immune system were proposed to have consequences in the design of TB vaccines [3] and to be in part responsible of the poor performance of immune-based diagnostic tools [4,5].

The cell envelope and secreted components of MTB are among the bacterial molecules most commonly described as potential biomarkers of the infection, or involved in host immune evasion. Mycobacteria possess a remarkably complex cell envelope consisting of a cytoplasmic membrane and a cell wall. These constitute an efficient permeability barrier that plays a crucial role in intrinsic drug resistance and contributes to the resilience of the pathogen in infected hosts [6]. Membrane and exported proteins are crucial players for maintenance and survival of bacterial organisms, and their contribution to pathogenesis and immunological responses make these proteins relevant targets for medical research [7]. In particular, these proteins are known to play pivotal roles in host-pathogen interactions and, therefore, represent potential drug targets and vaccine candidates [8].

The bulk of exported proteins in mycobacteria are transported by the general secretory Sec-translocase pathway. This is performed by recognition of the signal peptide in the nascent preprotein, which is subsequently transferred in an unfolded state to the machinery that executes its translocation across the membrane [9,10]. As in other bacteria, a further protein export system of mycobacteria is the Tat pathway, which exports folded preproteins with N-terminal signal peptides containing a twin-arginine motif [10]. Besides, mycobacteria utilize the specialized type VII secretion systems (T7SS) to export many of their important virulence proteins. The T7SS encompasses five homologous secretion systems (designated ESX-1 through ESX-5). Most pathogenic mycobacterial species, including the human pathogen *M*. *tuberculosis*, possess all five ESX systems [11,12]. The ability of MTB to subvert host immune defenses is related to the secretion of multiple virulence factors via the specialized T7SS [12].

Recent developments in mass spectrometry-based proteomics have highlighted the occurrence of numerous types of post-translational modifications (PTMs) in proteomes of prokaryotes which create an enormous diversity and complexity of gene products [13]. This PTMs, mainly glycosylation, lipidation and phosphorylation, are involved in signaling and response to stress, adaptation to changing environments, regulation of toxic and damaged proteins, protein localization and host-pathogen interactions. In MTB, more frequently O-glycosylation events have been reported [14], being this post-translational modification often found, in conjunction with acylation, in membrane lipoproteins [15]. A mechanistic model of this modification was proposed in which the initial glycosyl molecule is transferred to the hydroxyl oxygen of the acceptor Thr or Ser residue, a process catalyzed by the protein O-mannosyltransferase (PMT) (Rv1002c) [16]. Hereafter, further sugars are added one at a time, a process that in *M*. *smegmatis* was reported to be catalyzed by the mannosyltransferase PimE (encoded by the gene *Msmeg_5149*, homologous to the gene Rv1159 in *M*. *tuberculosis*) [17]. Sec-dependent secretion has been proposed to be linked to O-glycosylation [16], and this modification appears essential for MTB virulence, since Rv1002c deficient strains are highly attenuated in immunocompromised mice [17].

Despite the vital importance of glycosylated proteins in MTB pathogenesis, the current knowledge in this regard is still limited, and in culture filtrates of this pathogen a few secreted and cell wall-associated glycoproteins have been identified to date [15,18,19]. Initial evidence confirmed eight lipoprotein sequences of MTB proteins which conferred concanavalin A (ConA) binding to a chimeric reporter protein, including Apa (Rv1860), LpqH (Rv3763), Mpt83 (Rv2873) and PstS1 (Rv0934) [20]. Regarding the identification of O-glycosylated proteins in MTB secreted proteins a glycoproteomic approach reported 41 putative mannosylated proteins, being many of them lipoproteins, after ConA chromatography enrichment and 2D gel electrophoresis [18]. In a more recent glycoproteomic approach a ConA enrichment technique combined with the use of different collision energy dissociation techniques, allowed the identification of O-glycosylation sites in 13 MTB proteins [19], including Apa (Rv1860), 6 proteins found in the former screen using ConA chromatography [18] and 6 novel glycoproteins. Recent evidence using whole cell extracts revealed that glycosylation could be much more frequent than previously thought, explaining the phenotypic diversity and virulence in the *Mycobacterium tuberculosis* complex [14].

In this study we describe a straightforward methodology based on a high throughput label-free quantitative proteomic approach in order to provide a comprehensive identification, quantification and evaluation of the extent of O-glycosylation of proteins in *M*. *tuberculosis* H37Rv culture filtrate. The results presented here make focus on the principal exported and secreted virulence factors with the aim to contribute to a deep proteomic and glycoproteomic characterization of this relevant pathogen and to collaborate to a better understanding of the pathogenesis and survival strategies adopted by MTB.

## Materials and methods

### Mycobacterial strain and growth conditions

*Mycobacterium tuberculosis* H37Rv strain (ATCC^®^ 25618^™^) was grown for 3 weeks at 37°C in Lowenstein Jensen solid medium and after growth was achieved it was subcultured in Middlebrook 7H9 broth supplemented with albumin, dextrose, and catalase (ADC) enrichment (Difco, Detroit, MI, USA) for 12 days with gentle agitation at 37°C. Mycobacterial cells were pelleted at 4000xg for 15 min at 4°C and washed 3 times with cold phosphate-buffered saline. Mycobacterial cells were subsequently cultured as surface pellicles for 3 to 4 weeks at 37°C without shaking in 250 mL of Sauton minimal medium, a synthetic protein-free culture medium, which was prepared as previously described [21].

### Culture filtrate protein preparation

Bacterial cells were removed by centrifugation and culture filtrate protein (CFP) was prepared by filtering the supernatant through 0.2 μM pore size filters (Millipore, USA). After sterility testing of CFP in Mycobacteria Growth Indicator Tube (MGIT) supplemented with MGIT 960 supplement (BD, Bactec) for 42 days at 37°C in BD BACTEC^™^ MGIT^™^ automated mycobacterial detection system, CFP was concentrated using centrifugal filter devices (Macrosep Advance, 3kDa MWCO (Pall Corporation, USA)). Concentrated CFP was buffer exchanged to phosphate-buffered saline and total protein concentration was quantified by BCA (Pierce BCA Protein Assay Kit, Thermo Fischer Scientific). *M*. *tuberculosis* concentrated CFP samples diluted in SDS-PAGE loading buffer were loaded onto 15% SDS-PAGE and silver nitrate staining was performed as described elsewhere [22].

### Liquid chromatography tandem mass spectrometry (LC MS/MS)

Two replicas of *M*. *tuberculosis* CFP (25 μg) were loaded in SDS-PAGE 15% and stained with CBB G-250 as described elsewhere [23]. Six gel slices were excised from each lane according to protein density. In-gel Cys alkylation, in gel-digestion and peptide extraction was performed as described before [24]. Tryptic peptides were separated using nano-HPLC (UltiMate 3000, Thermo Scientific) coupled online with a Q-Exactive Plus hybrid quadrupole-Orbitrap mass spectrometer (Thermo Fischer Scientific). Peptide mixtures were injected into a trap column Acclaim PepMap 100, C18, 75 um ID, 20 mm length, 3 um particle size (Thermo Scientific) and separated into a Reprosil-Pur 120 C18-AQ, 3 μm (Dr. Maisch) self-packed column (75μm ID, 49 cm length) at a flow rate of 250 nL/min. Peptide elution was achieved with 105 min gradient from 5% to 55% of mobile phase B (A: 0.1% formic acid; B: 0.1% formic acid in 80% acetonitrile). The mass spectrometer was operated in data-dependent acquisition mode with automatic switching between MS and MS/MS scans. The full MS scans were acquired at 70K resolution with automatic gain control (AGC) target of 1 × 10^6^ ions between m/z = 200 to 2000 and were surveyed for a maximum injection time of 100 milliseconds (ms). Higher-energy collision dissociation (HCD) was used for peptide fragmentation at normalized collision energy set to 30. The MS/MS scans were performed using a data-dependent top12 method at a resolution of 17.5K with an AGC of 1 × 10^5^ ions at a maximum injection time of 50 ms and isolation window of 2.0 m/z units. A dynamic exclusion list with a dynamic exclusion duration of 45 s was applied.

### LC-MS/MS data analysis

LC-MS/MS data analysis was performed in accordance to the PatternLab for proteomics 4.0 software (http://www.patternlabforproteomics.org) data analysis protocol [25]. The proteome (n = 3993 proteins) from *M*. *tuberculosis* (Reference strain ATCC 25618/H37Rv UP000001584) was downloaded from Uniprot (March 2017) (https://www.uniprot.org/proteomes/). A target-reverse data-base including the 123 most common contaminants was generated using PatternLab’s database generation tool. Thermo raw files were searched against the database using the integrated Comet [26] search engine (2016.01rev.3) with the following parameters: mass tolerance from the measured precursor m/z(ppm): 40; enzyme: trypsin, enzyme specificity: semi-specific, missed cleavages: 2; variable modifications: methionine oxidation; fixed modifications: carbamidomethylation of cysteine. Peptide spectrum matches were then filtered using PatternLab’s Search Engine Processor (SEPro) module to achieve a list of identifications with less than 1% of false discovery rate (FDR) at the protein level [27]. Results were post-processed to only accept peptides with six or more residues and proteins with at least two different peptide spectrum matches. These last filters led to a FDR at the protein level lower than 1% for all search results. Proteins were further grouped according to a maximum parsimony criteria in order to identify protein clusters with shared peptides and to derive the minimal list of proteins [28]. Spectrum counts of proteins identified in each technical replicate were statistically compared with paired Mann-Whitney test.

For the O-glycosylation analysis raw files were searched against the same database using the parameters described above with the addition of the following variable modifications in S or T amino acid residues: Hex = 162.052824 Da, Hex-Hex = 324.1056 Da or Hex-Hex-Hex = 486.1584 Da. Monoisotopic mass of each neutral loss modification was defined in Comet search engine according to the values recorded in Unimod public domain database (http://www.unimod.org/). Each O-glycosylation was tested independently and a maximum of 2 modifications per peptide was allowed.

Peptide spectrum matches were filtered and post-processed using SEPro module, using the same parameters as described above and proteins were grouped according to a maximum parsimony criteria [28].

### Protein analysis

Identified proteins in each replicate were compared by area-proportional Venn Diagram comparison (BioVenn [29]) and a list of common proteins was generated. Further analysis only considered proteins present in both replicates of LC MS/MS analysis. SEPro module retrieved a list of protein identified with Uniprot code. Molecular weight, length, complete sequence, gene name and *M*. *tuberculosis* locus identified (Rv) was obtained using the Retrieve/ID mapping Tool of Uniprot website (https://www.uniprot.org/uploadlists/) [30]. Protein functional category was obtained by downloading *M*. *tuberculosis* H37Rv genome sequence Release 3 (2018-06-05) from Mycobrowser website (https://mycobrowser.epfl.ch/) [31].

### Protein O-glycosylation analysis

Proteins bearing O-glycosylated peptides in both replicates were compared by area-proportional Venn Diagram comparison (BioVenn [29]) and a list of common glycosylated proteins for each of the analyzed modifications, *i*.*e*. Hex, Hex-Hex and Hex-Hex-Hex, was generated. Further analysis was manually performed in order to identify common modified peptides in the list of common glycosylated proteins, as well as common modifications (as 1 peptide could contain up to two modifications). As a result of this analysis a list of proteins with common modifications was generated, consisting in proteins having the same modified peptide in both replicates. This list of O-glycosylated proteins was considered for subsequent analysis. For O-glycosylation site assignation the utility XDScoring of Patternlab for proteomics developed for statistical phosphopeptide site localization [32], was preliminary tested in our data.

### Signal peptide and transmembrane helices prediction

In order to identify potentially secreted proteins, the SignalP 5.0 Server (http://www.cbs.dtu.dk/services/SignalP/) was used to detect the presence of N-terminal signal sequences in the analyzed set of proteins. The organism group selected was gram-positive bacteria. This version of the Server, recently launched, incorporates a deep recurrent neural network-based approach that improves signal peptide (SP) prediction across all domains of life and classify them into three type of prokaryotic signal peptides: Sec/SPI (SP): standard secretory signal peptides transported by the Sec translocon and cleaved by Signal Peptidase I, Sec/SPII (LIPO): lipoprotein signal peptides transported by the Sec translocon and cleaved by Signal Peptidase II and Tat/SPI (TAT): signal peptides transported by the Tat translocon and cleaved by Signal Peptidase I [33]. If a signal peptide is predicted, the cleavage site (CS) position is also reported. *M*. *tuberculosis* H37Rv reference proteome (UP000001584) obtained from UniProt was also submitted to SignalP 5.0 signal peptide prediction [33]. Transmembrane helices in protein sequences were predicted by the TMHMM 2.0 algorithm (http://www.cbs.dtu.dk/services/TMHMM/).

### Estimation of protein abundance and comparative analysis

To estimate protein abundance Normalized Spectral Abundance Factor (NSAF) calculated with PatternLab for proteomics software was considered. NSAF allows for the estimation of protein abundance by dividing the sum of spectral counts for each identified protein by its length, thus determining the spectral abundance factor (SAF), and normalizing this value against the sum of the total protein SAFs in the sample [34,35]. Proteins were ordered according to their NSAF. NSAF values corresponding to percentile 75th, 90th and 95th were calculated, and the groups of proteins above these values were identified as P75%, P90% and P95% proteins, respectively. The list of proteins obtained in this study was compared with other proteomic studies [9,36,37] by Venn Diagram comparison (Venny 2.1, BioinfoGP [38]) and NSAF of proteins identified in all studies, 3 studies, 2 studies or only this study were statistically compared with unpaired Mann-Whitney test. The protein abundance determined for CFP identified in this study (NSAF) was compared with the protein abundance calculated for *M*. *tuberculosis* proteins identified in a previous study using the exponentially modified protein abundance index (emPAI) [9].

### Protein classification

Gene Onthology (GO) analysis of the culture filtrate proteins was performed with David Gene Functional Classification Tool [39,40] using the Cellular Component Ontology database and total proteins of *M*. *tuberculosis* H37Rv (NCBI:txid83332) as background. With this analysis principal categories of enriched terms (p<0.05) for P75%, P90%, P95% and total proteins were determined. Functional classification of culture filtrate proteins was performed according to functional categories of *M*. *tuberculosis* database Mycobrowser [31].

Proteins with O-glycosylation modifications were analyzed with David Gene Functional Classification Tool [39,40] using Cellular Component, Biological Processes and Molecular functions Ontology database and total proteins of *M*. *tuberculosis* H37Rv (NCBI:txid83332) as background.

### O-glycosylation validation

The same analytical workflow described for O-glycosylation analysis of our data was performed using the raw data files deposited at the ProteomeXchange Consortium with the dataset identifier PXD000111 [37]. This analysis was performed in order to compare the modified peptides identified in our work against additional biological replicates obtained in a previous work that extensively characterized culture filtrate proteins of *M*. *tuberculosis* H37Rv [37]. Additionally, some relevant scans corresponding to glycosylated peptides were searched in Mascot Server MS/MS Ions Search (Mascot, Matrix Science Limited [41]). Search was performed against NCBIprot (AA) database of all taxonomies. Search parameters were defined as peptide mass tolerance: ± 10 ppm, MS/MS mass tolerance: ± 0.15 Da, enzyme: semiTrypsin, fixed modifications: Carbamidomethyl (C), variable modifications: Hex (ST), Hex(2) (ST) or Hex(3) (ST), according to the searched modification. Other parameters were set to default values.

## Results and discussion

### Characterization of culture filtrate proteins using LC MS/MS

*M*. *tuberculosis* H37Rv was cultured following a classical method using Sauton minimal medium, a synthetic protein-free culture medium compatible with proteomic downstream analysis [21] and four different batches of culture filtrate proteins (CPF) were analyzed by gel electrophoresis and silver nitrate staining. An electrophoretic pattern showing a variety of proteins from approx. 10 kDa to 100 kDa was observed (S1A Fig). As similar patterns were observed with the different CFP preparations a composed sample was prepared for LC MS/MS analysis. A high throughput analysis was performed using a shotgun quantitative approach based on a liquid nano-HPLC and tandem mass spectrometry workflow. The proteins present in two technical replicates were resolved in SDS-PAGE and 6 different portions of each lane were further selected for LC MS/MS analysis (S1B Fig). For further analysis, each lane was batch-processed, including the different portions analyzed, in order to visualize the whole protein composition of culture filtrate. 1427 (0.28% FDR) and 1429 (0.41% FDR) different MTB proteins were detected in CFP(1) and CFP(2), respectively (S1 Table). The mass spectrometry proteomics data (raw data and search files) have been deposited at the MassIVE repository with the dataset MSV000084184 and announced via ProteomeXchange PXD014964 (doi:10.25345/C5PW8Q).

Qualitative comparison of both datasets using a Venn Diagram bioinformatic tool showed that 1314 MTB proteins (92%) were shared between both replicates (S1C Fig) and spectrum counts quantitative comparison showed that there were not statistical differences among them (S1D Fig). The full list of 1314 common proteins, which was used for further analysis, is provided in S1 Table. Proteins showed a wide distribution of molecular weights, however most of them were of low molecular weight (median 31.97 kDa, Q1 21.25 kDa, Q3 46.50 kDa), which was consistent with the profile observed in S1A and S1B Fig. Previous research has shown that the vast majority of protein spots resolved in 2D gel electrophoresis of *M*. *tuberculosis* H37Rv CFP were found in the molecular weight range of 6–70 kDa [21]. Moreover, consistent with our results, proteins identified by LC-MS/MS in a well characterized CFP, showed that the majority of the proteins were found in the 10–50 kDa range, with an average theoretical mass of 31.0 kDa [36].

### Protein classification using a quali-quantitative analysis

**Fig 1:**
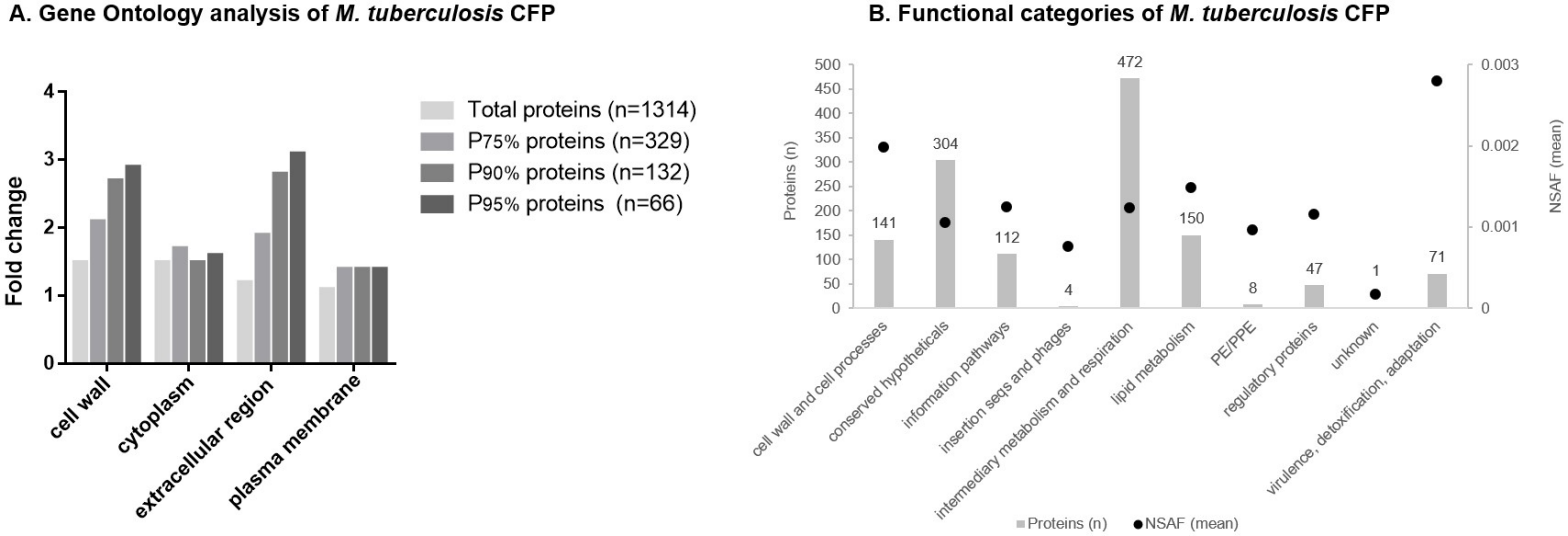
Quali-quantitative protein classification. (1A) Fold change of principal categories of enriched terms (p<0.05) obtained analyzing common proteins with David Gene Functional Classification Tool [39,40] using the Cellular Component Ontology database and *M*. *tuberculosis* H37Rv total proteins as background. Proteins were ordered considering normalized spectral abundance factor (NSAF) and percentile 75th, 90th and 95th NSAF were calculated. Fold change of the lists above each defined percentile (P75%, P90% and P95% proteins) analyzed using the same approach is shown. (1B) Functional categories of CFP according to *M*. *tuberculosis* database Mycobrowser [31]. Bars represent number of proteins corresponding to each category (number is indicated above each bar, scale in left axe) and dots represent mean NSAF of proteins in each category (scale is indicated in right axe).

Quantitative proteomics based on spectral counting methods are straightforward to employ and have been shown to correctly detect differences between samples [42]. In order to consider sample-to-sample variation obtained when carrying out replicate analyses, and the fact that longer proteins tend to have more peptide identifications than shorter proteins, Patternlab for Proteomics software uses NSAF (Normalized spectral abundance factor) [43] for spectral counting normalization. NSAF was shown to yield the most reproducible counts across technical and biological replicates [34]. Using the sum of NSAF of both replicates (Total NSAF, included in S1 Table) the common list of CFP was ordered according to protein abundance and arbitrarily grouped in 4 subgroups (P95%, P90%, P75% and total CFP), consisting of 66, 132, 329 and 1314 proteins, respectively. P95% comprised proteins above 95th percentile NSAF, thus representing the most abundant proteins in the sample. P90% and P75% comprised proteins above 90th and 75th percentile, respectively. These subgroups of proteins were functionally classified using Gene Ontology, Cellular Component analysis, and principal categories of enriched terms (p<0.05) were determined (Fig 1A). Considering the subgroup of total CFP proteins 4 principal categories (cell wall, cytoplasm, extracellular region and plasma membrane) were similarly enriched with respect to *M*. *tuberculosis* H37Rv total proteins used as background (fold change 1.5, 1.5, 1.2 and 1.1, respectively). However, when considering the subgroups of more abundant proteins, the categories cell wall and extracellular region showed a marked increase of fold enrichment with protein abundance, achieving these categories in P95% subgroup a fold enrichment of 2.9 (p = 8.3e-18) and 3.1 (p = 2.0e-8), respectively. This tendency was not observed in cytoplasm and plasma membrane categories. Thus, our analysis indicates that the subgroups of more abundant proteins contained mainly proteins of extracellular region and cell wall compartment.

The annotated *M*. *tuberculosis* H37Rv proteins have been classified into 12 distinct functional categories in the *M*. *tuberculosis* database Mycobrowser [31]. Functional classification of proteins identified in this study showed that proteins were distributed across ten of those functional groups (Fig 1B). Most of the identified proteins are involved in intermediary metabolism and respiration (35.9%). However, when protein abundance is considered, the category with the highest protein mean NSAF is virulence, detoxification, adaptation followed by cell wall and cell processes (Fig 1B). In particular, enzymes involved in detoxification of reactive oxygen intermediates (KatG (Rv1908c), SodA (Rv3846) and TxP (Rv1932)), which participate in the resistance of the bacterium to the oxidative stress inside host cells [44,45], are representatives of this functional category and belong to P95% protein subgroup.

Considering the search for pathogen-derived biomarkers for *M*. *tuberculosis* active diagnosis, we looked in the list of CFP for principal protein antigens detected in clinical samples [46], confirming the presence of 11 out of 12. Moreover, these putative biomarkers exhibited on average a high NSAF, being 10 of them in the P90% subgroup. This information however, should be taken into account cautiously, since biomarkers related to pathogen infection may not necessarily correspond to *in vitro* culture highly-expressed proteins.

In sum, the quali-quantitative analysis of the LC MS/MS analysis presented here served to evidence a global correlation between highly secreted proteins and their biological implication in key pathways related to mycobacterial pathogenicity. Particular stress or starvation *in vitro* conditions [37], hypoxic or non-replicative persistence models, different MTB lineages, native and mutant strains, as well as outbreak-related clinical isolates could be confidently analyzed and compared by means of this approach, bringing answers to scientific questions related to MTB virulence, persistence and drug resistance.

### Prediction of secreted proteins

Given the results obtained the question arises whether the presence of certain proteins in CFP is due to bacterial leakage/autolysis in combination with high levels of protein expression and extracellular stability, rather than to protein-specific export mechanisms. Using SignalP 5.0 peptide prediction server [33] a total of 392 proteins were predicted to have one type of signal peptide in *M*. *tuberculosis* proteome (207 SP, 113 LIPO and 72 TAT). Of those we identified 140 in CFP (62 SP, 53 LIPO and 25 TAT), being many of them known secreted proteins, particularly FbpA (Rv3804c), FbpB (Rv1886c), FbpC (Rv0129c), Apa (Rv1860), Mpt64 (Rv1980c), PstS1 (Rv0934), LpqH (Rv3736), among others (S2 Table).

To export proteins across its unique cell wall, besides the signal-sequence-dependent secretory pathways, mycobacteria utilize up to five distinct ESX secretion systems (designated ESX-1 through ESX-5, referred to as the type VII secretion system: T7SS), with various functions in virulence, iron acquisition, and cell surface decoration [11]. The ESX-1 system was the first of the T7SS to be identified and is responsible for the secretion of EsxA (6 kDa early secretory antigenic target, ESAT-6, Rv3875) and EsxB (Rv3874) [47]. Proteins belonging to ESX secretion systems gene clusters as well as closely related PE and PPE multigene families are *M*. *tuberculosis* secreted proteins that do not have classical secretion signals [12,48]. PE and PPE proteins are acidic, glycine-rich proteins, that are unique to mycobacteria, and significantly expanded in slow-growing pathogenic mycobacteria [48,49]. The T7SS is responsible of the export of PE and PPE proteins, mainly through the ESX-5 system [10,50]. We identified in CFP several proteins of ESAT-6 family, including EsxA (Rv3875) and EsxB (Rv3874), and various proteins of ESX-1 secretion system which count with experimental evidence of being secreted [30]. None of those were predicted by SignalP to contain a signal peptide (S2 Table). Finally, we detected 8 PE and PPE family proteins in our sample, from which 3 were predicted to have a signal peptide (S2 Table).

The presence in our CFP sample of several leaderless proteins, many of them with high level of expression, could reflect some extent of bacterial autolysis. Indeed, different autolysis markers were detected in our protein list, including GroEL (Rv0440), L-lactate dehydrogenase (Rv1872c), isocitrate dehydrogenase (Rv3339c) [51], glutamine synthetase GlnA1 (Rv2220), superoxide dismutase SodA (Rv3846), bacterioferritin Bfr (Rv1876) and malate dehydrogenase Mdh (Rv1240) [52]. In particular, the presence of SodA and GlnA1 in culture filtrate of actively growing MTB culture was described as not due to a protein-specific export mechanism, but rather to bacterial leakage or autolysis. The extracellular abundance of these enzymes was additionally related to their high level of expression and stability [52].

In summary, various proteins with signal peptides were detected in our sample and several other proteins related to T7SS were identified. The SignalP 5.0 server was a suitable approach in order to predict secreted proteins with classical signal peptides but it has limitations to analyze proteins bearing non-classical secretion signals. Besides, different autolysis protein markers were identified, evidencing certain degree of bacterial lysis probably combined with high protein expression and extracellular stability.

### Integrative analysis with previous proteomic studies

Former research studies, which used different and complementary approaches to characterize *M*. *tuberculosis H37Rv* CFP, were compared against our results [9,36,37]. Malen *et al*. evaluated a culture filtrate of *M*. *tuberculosis* H37Rv, considerably enriched for secreted proteins and identified 257 proteins (254 annotated with Rv identifier) [36]. Later, de Souza *et al*. performed a proteomic screening of proteins in culture filtrate, membrane fraction and whole cell lysate of *M*. *tuberculosis*, identifying 2182 proteins in the different fractions, and specifically 458 proteins in CFP [9]. In a recent report, Albrethsen *et al*. characterized the culture filtrate proteome of *M*. *tuberculosis* H37Rv bacteria in normal log-phase growth and after 6 weeks of nutrient starvation and detected 1362 different proteins [37]. Through this comparison we evidenced a common group of 122 proteins consistently detected (S2A Fig). Among them, 41 belong to the P90% subgroup indicating that these are highly abundant proteins (S3 Table). Several relevant proteins in terms of their implication in virulence, vaccine design and diagnosis are included in this common group (S3 Table). Besides, in this group, 50% of the proteins were predicted to have one type of signal peptide, whereas in the group of 221 particular proteins (not identified in the 3 studies considered in the comparison) less than 6% (n = 13) of the proteins were predicted as having a secretion signal peptide (S3 Table). Particular proteins were mostly classified as related to intermediate metabolism and respiration (n = 62), a fact that could indicate that most are cytoplasmatic proteins, observed in CFP due to bacterial lysis. However, interestingly, 18 particular proteins were classified as related to cell wall and cell processes, including some proteins of the T7SS systems, and this category exhibited the highest protein mean NSAF (S3 Table), consistent with their preferred location in culture filtrate.

Proteins identified in the four studies (N = 4) are on average more abundant than proteins identified in the other groups analyzed (N = 3, N = 2 or N = 1) (S2B Fig). Moreover, proteins identified in at least 2 studies (N = 3 or N = 2) are globally more abundant than proteins identified exclusively in the present work. The fact that proteins identified only in this study are mostly predicted as not having signal peptide, as well as poorly abundant, confirmed that bacterial lysis occurred during culture. It is important however to note that all autolysis markers identified in our sample were found in at least one of the previous studies, suggesting that bacterial lysis is a common observation in MTB culture filtrate.

By comparing our data against the proteomic quantitative approach performed by de Souza *et al* [9] we identified a subgroup of highly represented proteins consisting of those also present in the three fractions studied by them, *i*.*e*. culture filtrate, membrane fraction and whole cell lysate. This subgroup accounted for 43.2% of protein abundance expressed as NSAF in this work and 29.2% of emPAI calculated by the cited research (S4 Table).

As a whole these observations show that the CFP prepared in the present work exhibited a good correlation with previous studies, both in terms of qualitative proteomic composition as well as in relation to the quantitative estimation of protein abundance. Proteins highly represented in our sample are proteins either frequently identified by others using complementary approaches in culture filtrates of MTB, and thus confirming that our sample is enriched in proteins that the bacteria does secrete, or ubiquitously detected in different *M*. *tuberculosis* cellular fractions, indicating that these could represent highly expressed proteins.

By this integrative analysis we evidenced 30 proteins not annotated with proteomic data in Mycobrowser website (Release 3 (2018-06-05)) [31] (S5 Table). This list, principally composed by proteins classified as conserved hypotheticals, includes the ESX-3 secretion-associated protein EspG3 (Rv0289) identified with 4 unique peptides in CFP(1) and 5 unique peptides in CFP(2) and the Two component sensor histidine kinase DosT (Rv2027c) identified with 2 unique peptides in each replicate. The information presented here is expected to be included in this relevant mycobacterial database in order to be easily available to research community. Further comparison of these proteins with the results obtained in a proteome-wide scale approach based on SWATH mass spectrometry [53] allow us the identification, to the best of our knowledge, of 8 proteins without previous evidence of expression at the protein level. All of them were identified with at least two unique peptides (S5 Table). Sequence coverage and peptide spectra of possible toxin MazF7 (Rv2063A), a ribonuclease belonging to toxin-antitoxin system [54], and Acyl carrier protein (ACP) MbtL (Rv1344), an enzyme thought to be involved in fatty acid biosynthesis [55], are presented in S3 Fig.

### O-glycosylation analysis

To conclude our integrative analysis of MTB culture filtrate, the presence of O-mannosylated proteins was evaluated. To this date, only mannose has been fully validated as the sugar decorating glycosylated proteins in *M*. *tuberculosis*. Although the pentose sugar arabinose, as well as other hexose sugars like galactose or glucose, were described as a potential glycan in 45 kDa antigen (Apa (Rv1860) [56], only mannose was confirmed as the covalently bounded sugar [15,57]. Recently, in proteins derived from MTB whole cell extracts other O-linked sugars, as well as several N-glycosylation events, were reported [14]. However, no further validation of the newly identified sugars is currently available [15]. Taken this into account, our analysis was restricted to the evaluation of peptides containing hexoses and multi hexose modifications (up to 3 hexoses at each glycosylation site) [15,57].

Our rationale was that the nano LC MS/MS technology used in this work, by having more than four orders of magnitude intrascan dynamic range and a femtogram-level sensitivity, would allow the direct identification of modified peptides, without affinity-based strategies for glycosylated protein enrichment. A similar approach was exploited to evaluate the whole cell lysate of different MTB lineages [14], and in a complementary way the present work evaluated glycosylation of non-previously enriched culture filtrate proteins.

O-glycosylation profile analysis revealed the presence of 69 common glycosylation events in 61 common modified peptides in both replicas of MTB culture filtrate (Table 1). The O-glycosylated common peptides were identified in 167 scans, consisting in at least 2 scans per peptide (1 scan per replica) and a maximum of 8 scans in the case of Hex-Hex-Hex modification of Alanine and proline rich secreted protein Apa (Rv1860) (S6 Table). In many cases the unmodified peptide was identified along with the modified peptide, indicating that glycosylated and unglycosylated proteins isoforms are present (some examples are shown in S4 Fig), as was reported for the conserved lipoprotein LprG [58].

**Table 1:** O-glycosylation profile of *M*. *tuberculosis* culture filtrate proteins identified by LC MS/MS.

Modification		Hex	Hex-Hex	Hex-Hex-Hex
**Replica # 1**	**Modified Peptides (n)**	268	94	68
	**Peptide FDR (%, n/N)**	**0.15** (27/17879)	**0.13** (22/17513)	**0.14** (24/17635)
	**Modified Proteins (n)**	212	91	62
	**Protein FDR (%, n/N)**	**0.94** (14/1494)	**0.95** (14/1467)	**1.00** (15/1505)
**Replica #2**	**Modified Peptides (n)**	107	72	66
	**Peptide FDR (%, n/N)**	**0.13** (22/16603)	**0.15** (25/16614)	**0.12** (20/16716)
	**Modified Proteins (n)**	95	67	57
	**Protein FDR (%, n/N)**	**0.99** (15/1509)	**0.99** (15/1511)	**0.99** (15/1515)
**Common analysis**	**Common modified proteins (n)**	36	23	15
	**Common modified peptides (n)**	29	17	15
	**Common modifications (n)**	35	18	16
	**Proteins with common modifications (n)**	24	17	13

FDR: False discovery rate, n: number, N: total number.

**Fig 2:**
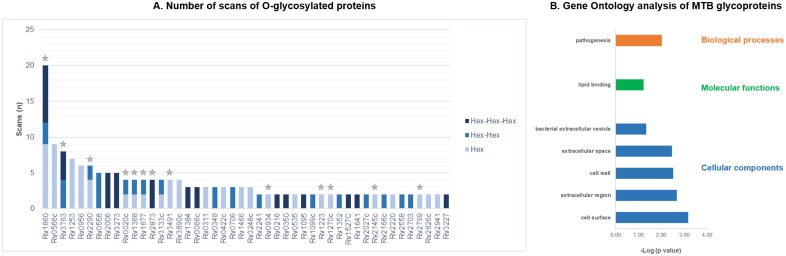
Description of O-glycosylated proteins in *M*. *tuberculosis* CFP. (2A) Scans of O-glycosylated peptides identified in MTB culture filtrate proteins. Each analyzed modification is displayed with a different bar color. Individual scans of both replicates were considered (n = 46). Previously known O-glycosylated proteins (n = 13) are indicated with a grey star. (2B) Gene Ontology analysis of MTB culture filtrate glycoproteins. Principal categories of enriched terms (p<0.05) obtained analyzing proteins with common glycosylation in both replicates with David Gene Functional Classification Tool [39,40] using Molecular Functions, Biological Processes and Cellular Component Ontology database and *M*. *tuberculosis* H37Rv total proteins as background.

O-glycosylation modifications were detected in 46 different MTB culture filtrate proteins including 7 lipoglycoproteins (S6 Table). 23 of the O-glycosylated proteins presented at least 3 scans of the modified peptide and 7 exhibited more than one of the searched modifications (Fig 2A). Of those, 10 proteins have previous evidence of being mannosylated, summarized in Mehaffy *et al* [15], and 3 additional proteins (HtrA (Rv1223), DsbF (Rv1677) and Wag31 (Rv2145c)) were found with the same modification in a later report [14]. It is Interesting to highlight the high number of scans of modified peptides corresponding to Apa (Rv1860), a largely characterized secreted mannosylated glycoprotein [56,57]. It is currently believed that mannosylated proteins can act as potential adhesins and it was demonstrated that Apa is associated with the cell wall and binds lung surfactant protein A (SP-A) and other immune system C-TLs containing homologous functional domains [59]. In addition, the 19 kDa lipoprotein antigen precursor LpqH (Rv3763), also showing an important number of Hex-Hex and Hex-Hex-Hex modified peptides, is a well-known glycosylated protein exposed in the bacterial cell envelope, that was postulated to be used by mycobacteria to enable their entry into the macrophage through interaction with mannose receptors (MRs) of this host cells [60].

### O-glycosylated proteins classification

Glycosylation plays a significant role in MTB adaptive processes and in cellular recognition between the pathogen and its host [59,60]. Significantly enriched biological processes and molecular function categories of the glycoproteins identified here were, respectively, pathogenesis (GlnA1, LpqH, PstS1 and DevR are some proteins assigned to this category) and lipid binding (including lipoproteins LprA and LprF) (Fig 2B). As expected, our GO analysis showed that most of the glycoproteins identified were preferentially localized in the cell surface and extracellular region (Fig 2B).

O-glycosylated proteins identified in this study are distributed in 7 functional categories according to *M*. *tuberculosis* database [35] (Table 2). Most of them are involved in intermediary metabolism and respiration (n = 15) and in cell wall and cell processes (n = 11). Particularly, to this latter category belong the vast majority of known O-mannosylated proteins (Table 2).

**Table 2:** Functional categories of predicted O-glycosylated proteins according to *M*. *tuberculosis* database (Mycobrowser [31]).

Functional category	Protein	Locus	Predicted Hex position	Predicted Signal peptide^a^	TMHHM no.^b^	References ^c^
**Cell wall and cell processes**	PstS1	Rv0934	S299	LIPO(Sec/SPII)		[18,20]
	LprA	Rv1270c	T40	LIPO(Sec/SPII)	1	[18,19]
	LprF	Rv1368	S50 & S53	LIPO(Sec/SPII)	1	[14,18]
	DsbF	Rv1677	T33 & T40	LIPO(Sec/SPII)		[14]
	Apa	Rv1860	T313, T315 & T316	SP(Sec/SPI)	1	[14,18,19]
	Wag31	Rv2145c	S192	NO		[14]
	LppO	Rv2290	T73 & T75	LIPO(Sec/SPII)		[14,19]
	Rv2799	Rv2799	T73	NO	1	[14,18,19]
	Mpt83	Rv2873	T49	LIPO(Sec/SPII)		[18,20]
	LpqH	Rv3763	S31, T34 & T35	LIPO(Sec/SPII)		[18,20]
	EsxC	Rv3890c	S35	NO		This work
**Virulence, detoxification, adaptation**	DnaK	Rv0350	T402	NO		This work
	OtsB1	Rv2006	T148 & S149	NO		This work
**Information pathways**	RplV	Rv0706	S43	NO		This work
	DeaD	Rv1253	T263 & T294	NO		This work
	InfC	Rv1641	S114	NO		This work
	SigA	Rv2703	S83	NO		This work
**Lipid metabolism**	Pks5	Rv1527c	T810	LIPO(Sec/SPII)		This work
	FadD28	Rv2941	T500	NO		This work
**Regulatory proteins**	FhaA	Rv0020c	S332 & S336	NO		[18]
	Rv0348	Rv0348	T115	NO		This work
	DosT	Rv2027c	S421	NO		This work
	DesvR	Rv3133c	S148, T151 & T156	NO		This work
**Intermediary metabolism and respiration**	Icd2	Rv0066c	S651	NO		This work
	Rv0216	Rv0216	S122	NO		This work
	ThiD	Rv0422c	T2	NO		This work
	PnP	Rv0535	T142	NO		This work
	MenH	Rv0558	S32	NO		This work
	PurN	Rv0956	S24	NO		This work
	PhoH2	Rv1095	T309	NO		This work
	GlpX	Rv1099c	S169	NO		This work
	HtrA	Rv1223	S212	NO	1	[14]
	CarB	Rv1384	T409	LIPO(Sec/SPII)		This work
	GlnA1	Rv2220	T36	NO		This work
	AceE	Rv2241	S32	NO		This work
	AroA	Rv3227	S349	NO		This work
	SahH	Rv3248c	T473	NO		This work
	Rv3273	Rv3273	S735	NO	10	This work
**Conserved hypotheticals**	Rv0311	Rv0311	S10	NO		This work
	Rv0566c	Rv0566c	T52, S53 & T55	NO		This work
	Rv1352	Rv1352	T23	SP(Sec/SPI)	1	This work
	Rv1466	Rv1466	S5	NO		This work
	Rv2166c	Rv2166c	S39	NO		This work
	Rv2558	Rv2558	T82	NO		This work
	Rv2826c	Rv2826c	S192	NO		This work
	Rv3491	Rv3491	S167 & S176	SP(Sec/SPI)	1	[14,18,19]

^*a*^ Number of transmembrane helices predicted by TMHMM 2.0 server (http://www.cbs.dtu.dk/services/TMHMM/).

^*b*^ SignalP 5.0 software prediction of signal peptide (http://www.cbs.dtu.dk/services/SignalP/.

^*c*^ Proteins with previous evidence of O-glycosylation are referenced.

The occurrence of some cytosolic glycosylated proteins in our sample may be associated with partial cellular lysis, as mentioned above. However, it is important to note that the presence of this modification in proteins without signal peptide is not expected, since glycosylation has been related to sec-dependent secretion [16]. Coincident with our results, some glycoproteins without signal peptide or transmembrane helices have been previously described, two of them also detected in our study (Table 2) [14,18]. In addition, it was demonstrated that the protein O-mannosyl transferase (Rv1002c) deficiency may have broader implications in the physiology and virulence of the mycobacteria, by combining decreased levels of immuno-dominant glycosylated proteins and altered bacterial cellular pathways, most notably amino acid biosynthesis [15]. Aiding to these results, our data indicate that the variability of substrates related to the glycosylation pathway in MTB is greater than expected, a fact also observed in Birnahu *et al*. report [14] and in the glycoproteome characterization of the related Gram positive *Streptomyces coelicolor* [61].

### O-glycosylation validation and site assignation

Of the 46 identified glycoproteins, 9 were proposed as such in the ConA-lectin affinity capture approach performed by Gonzalez-Zamorano *et al*. [18], including several lipoproteins, whereas 5 have been identified in the glycoproteomic analysis of Smith *et al*. [19], where O-linked glycosylation sites were manually assigned after extensive data curation (Table 2). A comparison of O-glycosylation site assignation was performed, although it is important to note that the precise O-glycosylation site assignation is hampered by the fact that collision energies used for peptide fragmentation cause the breakage of the weaker O-glycosydic bond leaving behind mostly unmodified fragments (glycosylation site p-value is presented in S6 Table). Our results are in good agreement in the case of the 5 glycosylated proteins in common, both in regard to O-glycosylated peptide as well as O-glycosylated site identification (S7 Table). Besides, 9 proteins of our list were described in the glycoproteomic analysis of Birhanu *et al*. [14] with the same type of O-glycosylation, and 5 with the same O-glycosylation site (S7 Table). Of those, we identified the same mono- or polyhexose modifications in DsbF (Rv1677), a probable conserved lipoprotein (S5 Fig), confirming that the glycosylation they encountered in whole cell extract of MTB is also present in culture filtrate.

**Fig 3:**
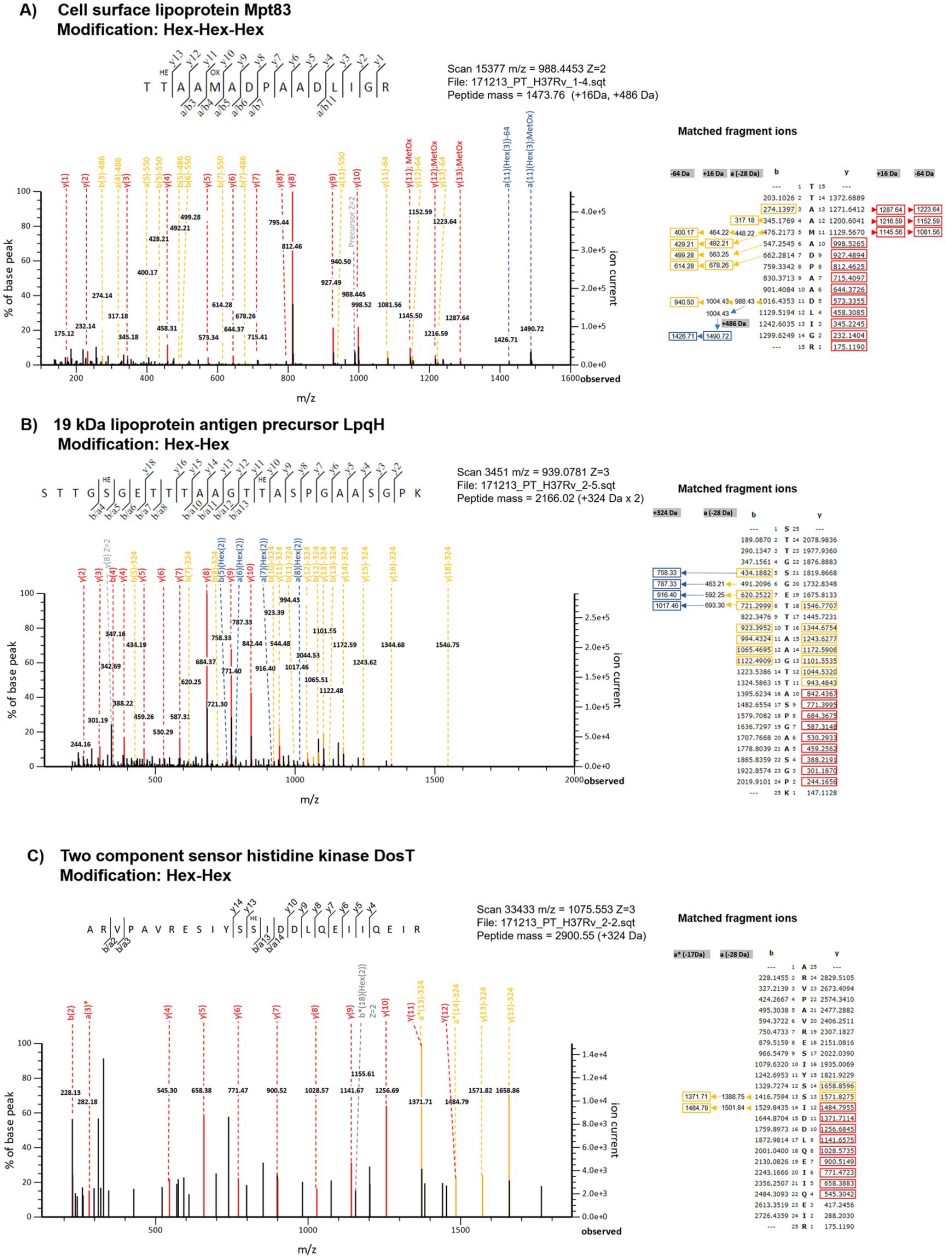
Glycopeptide spectra validation. Peptide fragmentation spectrum of (3A) Mpt83 (Hex-Hex-Hex modification), (3B) LpqH (Hex-Hex modification) and (3C) DosT (Hex-Hex modification). Spectra statistically confirmed by Mascot Server MS/MS Ions Search (HE = Hex(3) or Hex(2)). Fragment ions matches obtained in Mascot Server are indicated in each adjacent table. Color code: Red: unmodified ions, orange: ions with neutral losses, blue: ions bearing modifications, grey: charged ions/precursor assigned in Patternlab for Proteomics.

By analyzing proteins which O-glycosylation site was assigned measuring ConA reactivity through peptide cassette sequences screening [20], we confirmed our assignation for LpqH (Rv3763) and Mpt83 (Rv2873). Due to their relevance in *M*. *tuberculosis* virulence and immune modulation [62], manual validation of peptide spectra of Mpt83 and LpqH, including peptide ions fragment matches, are presented in Fig 3A and 3B, respectively. Although both proteins are largely evidenced as being O-glycosylated due to their interaction with ConA, as native proteins [18] or after heterologous expression in *M*. *smegmatis* [63–65], to our knowledge this is the first direct glycoproteomic identification in culture filtrate of MTB, of Mpt83 and LpqH derived O-glycosylated peptides. In both cases, O-glycosylation site assignation is coincident with the evidence in *M*. *smegmatis* model [63,65].

Another interesting protein in terms of its proposed role as active infection biomarker is PstS1, a periplasmic lipoprotein involved in phosphate transport across the membrane. It has been identified as a ConA interacting protein [18,20] and its immunoreactivity was proposed to be related to O-mannosylation [66], but direct evidence of its O-glycosylation in culture filtrate in first provided here (S5 Fig). Interestingly, the O-glycosylation site assignation differs from what was observed in the mycobacterial cassette expression system [20] or in a *P*. *pastoris* recombinant version of this protein [66].

Furthermore, we looked for O-glycosylated proteins in the raw data files deposited by Albrethsen *et al*. [37] at the ProteomeXchange Consortium. By means of this approach we confirmed 17 modified peptides (38 scans) in common with our results, corresponding to 8 proteins. Except for the adenosylhomocysteinase SahH (Rv3248c)—an enzyme involved in the L-homocysteine biosynthesis -, the rest of those proteins were evidenced as O-glycosylated in previous reports (S8 Table).

In brief, we are reporting 33 novel O-glycosylated proteins including hexose and multi-hexose modifications (Table 2). S5 Fig shows several examples of modified peptides spectra with good scores of known and novel glycoproteins. The scan number of each modified peptide spectra is supplied in S6 Table to access the remaining spectra in the publicly available raw data (doi:10.25345/C5PW8Q).

Considering novel O-mannosylated proteins identified in this study, a DosT (Rv2027c) O-glycosylated peptide spectrum bearing two hexoses was manually validated, as this protein has not previous proteomic annotation in Mycobrowser database (Fig 3C). DosT is a hypoxia sensor histidine kinase of the two component regulatory system DevRS/DosT which is essential for mycobacterial entry into and survival in the latent, dormant state [67,68]. The glycoproteomic study from Birhanu *et al*. described this protein as bearing two other different types of O-linked sugars [14], but Hex-Hex modification in that protein has not been reported previously. DevR, also found O-glycosylated by us (S5 Fig), is a regulatory protein induced by DosT under hypoxia. It is required for survival of MTB under hypoxic conditions and for its transition to normoxic metabolism [69]. Further work to validate this observation is warranted, as the dormancy survival regulator system is an attractive target for persistent *M*. *tuberculosis* infection treatment.

In summary, the information presented here serve to aid in the glycoproteomic characterization of this relevant pathogen, confirming previous knowledge and enlarging the set of putative MTB O-glycosylated proteins.

## Conclusion

Membrane and exported proteins are crucial players for maintenance and survival of bacterial organisms in infected hosts, and their contribution to pathogenesis and immunological responses make these proteins relevant targets for biomedical research [7]. Consistently, various of the proteins identified in *M*. *tuberculosis* CFP were proposed as relevant mycobacterial virulence factors [44], putative active infection biomarkers [46] or vaccine candidates [70,71].

The shotgun proteomic approach employed in this work allowed a deep comprehension of *M*. *tuberculosis* H37Rv culture filtrate proteins by reporting proteomic evidence in this sub-fraction for 1314 proteins. In that sense it is important to note that although this method is highly sensitive, specificity was prioritized by selecting as post-processing criteria only proteins with at least two different peptide spectrum matches.

In addition to proteins that have not been reported in *M*. *tuberculosis* H37Rv CFP, we also found proteins consistently detected in previous proteomic studies which were further confirmed as highly abundant proteins. Many of them were detected in culture filtrates of MTB or in different *M*. *tuberculosis* cellular fractions, including membrane fraction and whole cell lysate. This suggests that two complementary pathways are accounting for our observations. On one hand, the abundance of certain proteins in CFP appeared to be truly related to protein-specific export mechanisms, while on the other hand the presence of cytoplasmic markers in our sample evidenced the occurrence of bacterial autolysis combined with high levels of protein expression and extracellular stability. Nevertheless, the GO ontology Cellular Component analysis and the integrative analysis performed with relevant research papers confirmed that our sample is indeed enriched in proteins that the bacteria secretes to the extracellular space. Supporting this, we could identify several proteins with predicted N-terminal signal peptide indicating that these are targeted to the secretory pathways [72], as well as various proteins belonging to the ESX secretion systems and to PE and PPE families, known to be secreted by T7SS, but recognized for not having classical secretion signals [48].

Moreover, the quali-quantitative analysis performed showed a global correlation between highly secreted proteins and their implication in pathways related to virulence, detoxification and adaptation. This approach could be replicated in the future in order to answer remaining questions related to MTB pathogenicity.

Given the increasing evidence indicating that glycosylated proteins are often immune-dominant antigens with a key roles in MTB virulence and host-pathogen interactions [13,15], our integrative analysis also sought to expand the current knowledge in relation to the glycoproteins present in the culture filtrate of this pathogen. We described the identification of 69 glycosylation events, including hexose and multi-hexose modifications, in 46 MTB proteins. In particular, several lipoproteins were found glycosylated in culture filtrate. Lipoproteins have been shown to play key roles in adhesion to host cells, modulation of inflammatory processes, and translocation of virulence factors into host cells [73]. The growing evidence of glycosylation of mycobacterial lipoproteins including the results presented here, indicates that glycosylation plays a significant role in the function and regulation of this group of proteins. Along with lipoproteins, other relevant glycoproteins identified were mainly involved in pathogenesis and cell wall processes. Direct O-mannosylation proteomic evidence was supplied for various known glycoproteins and several novel proteins were predicted as bearing hexose-linked modifications. Protein glycosylation data presented here, including the coexistence of related protein glycoforms evidenced in this work, should be considered for designing antibody-based diagnostic tests targeting *M*. *tuberculosis* antigens. Besides, as reported for other pathogens [74,75], protein glycosylation diversity could be a key mechanism to provide antigenic variability aiding in the immune subversion of this pathogen.

Our study provided an integrative evaluation of MTB culture filtrate proteins, bringing evidence of the expression of some proteins not previously detected at protein level, and confirming and enlarging the database of O-glycosylated proteins. Although additional functional studies will be required to understand the potential relevance of the novel described glycoproteins in pathogen biology, this information may raise new questions on the role of protein O-glycosylation in the virulence and persistence of MTB, as well as it will contribute to deepen the knowledge of its main biomarkers, virulence factors and vaccine candidates.

## Supporting information

S1 Raw images (PDF)

S1 Fig Analysis of *M*. *tuberculosis* CFP by liquid chromatography tandem mass spectrometry (LC-MS/MS). **S1A**: *M*. *tuberculosis* CFP analysis by 1D SDS-PAGE 15% and silver nitrate staining. **S1B**: *M*. *tuberculosis* CFP analysis by 1D SDS-PAGE 15% and CCB G-250 staining. **S1C**: Spectrum counts of proteins identified in each technical replicate. **S1D**: Analysis of proteins identified in each replicate by area-proportional Venn Diagram comparison [29].
(TIF)

S2 Fig Comparison of *M*. *tuberculosis* CFP with other relevant proteomic studies. **S2A**: Analysis of *M*. *tuberculosis* CFP protein list (CFP TB: this study) versus other proteomic studies of *M*. *tuberculosis* CPF. **S2B**: Protein abundance estimation of proteins identified this study (CFP TB) and in all of the three other studies evaluated (N = 4), in this study and in two other studies (N = 3), in this study and in one other study (N = 2), or only in this study (N = 1).
(TIF)

S3 Fig Sequence coverage and representative spectra of possible toxin MazF7 (Rv2063A) and Acyl carrier protein (ACP) MbtL (Rv1344). (PDF)

S4 Fig Proteins showing glycosylated and unglycosylated equivalent peptides. Some protein examples are shown: 1) Apa (modification: Hex), 2) LprF (modification: Hex), LppO (modification: Hex-Hex), Apa (modification: Hex-Hex-Hex).
(PDF)

S5 Fig Scans of glycosylated peptides either confirmed in Mascot Server MS/MS Ions search against NCBIprot (AA) or visualized in mass spectrum viewer (PatternLab for proteomics). Some examples are shown: 1) DsbF (modification Hex-Hex), 2) LppO (modification: Hex), 3) PstS1 (modification Hex), 4) FhaA (modifcation Hex), 5) DevR (modification Hex-Hex), 6) DnaK (modification Hex-Hex-Hex), 7) Rv3273 (modification Hex-Hex-Hex), 8) Icd2 (modification Hex-Hex-Hex), 9) EsxC (modification Hex), 10) SahH (modification Hex), 11) DeaD (modification Hex), 12 Pks5 (modification Hex-Hex-Hex), 13) Wag31 (modification Hex), 14) GlnA1 (modification Hex), 15) AceE (modification Hex-Hex), 16) FadD28 (modification Hex), 17) Rv3491 (modification Hex), 18) Rv1352 (modification Hex-Hex), 19) CarB (modification Hex-Hex-Hex).
(PDF)

S1 Table Proteins identified with nano-HPLC MS/MS. Sheet 1) Common proteins list including Uniprot identification, protein description, protein length and molecular weight, gene name and *M*. *tuberculosis H37Rv* gene annotation (Rv) of Sanger Institut (http://sanger.ac.uk/projects/M_tuberculosis/Gene_list/). Sheet 2) Proteins identified in replica CFP(1), Sheet 3) Proteins identified in replica CFP(2), both lists including Uniprot identification as obtained in Patternlab for Proteomics, sequence count, spectrum count, number of unique peptides, protein coverage and protein description. Sheet 3 and Sheet 4) Values used to build Fig 1A and 1B, respectively.
(XLSX)

S2 Table Proteins with predicted signal peptides. Sheet 1) Signal peptide prediction (SignalP 5.0) in *M*. *tuberculosis* H37Rv reference proteome (UP000001584), Sheet 2) Signal peptide prediction (SignalP 5.0) in *M*. *tuberculosis* H37Rv CFP, Sheet 3) Proteins in *M*. *tuberculosis* H37Rv CFP with signal peptides predicted with SignalP 5.0.
(XLSX)

S3 Table Integrative analysis of CFP proteins. Sheet 1) Common proteins detected in *M*. *tuberculosis* CFP, Sheet 2) Proteins not detected in de Souza, Malen and Alberthsen analysis of *M*. *tuberculosis* CFP, Sheet 3) Signal Peptide Analysis, Sheet 4) Functional analysis, Sheet 5) Values used to build S2 Fig, Sheet 6) Values used to build S4 Table.
(XLSX)

S4 Table Protein abundance comparison against de Souza *et al*, 2011. Comparison of our proteomic data against the proteomic quantitative approach performed by de Souza *et al*, 2011 [9].
(DOCX)

S5 Table Proteins without proteomic annotation in Mycobrowser and/or not previously detected at proteomic level. Sheet 1) Proteins identified in *M*. *tuberculosis* H37Rv CFP without proteomic annotation in Mycobrowser (Release 3 (2018-06-05)) [31]. Sheet 2) Proteins in *M*. *tuberculosis* H37Rv CFP without previous evidence of expression at protein level, Sheet 3) Scans of peptides confirming proteins identified in *M*. *tuberculosis* H37Rv CFP without previous evidence at protein level.
(XLSX)

S6 Table Scans of O-glycosylated peptides in *M*. *tuberculosis* H37Rv culture filtrate proteins. Sheet 1) Total scans of O-glycosylated peptides. Sheet 2) Scans of O-glycosylated peptides belonging to lipoglycoproteins. Each table includes the File name where the scan was identified, the scan number, peptide charge (Z), measured and theorical mass and the difference (in ppm), scores (primary, secondary, etc), peptide sequence, modification (glycan), glycosylation site p-value, protein and gene data. Sheet 3 and Sheet 4) Values used to build Fig 2A and 2B, respectively.
(XLSX)

S7 Table O-glycosylation site comparison with available literature. O-glycosylation site comparison against Smith *et al*., 2014 [19], Birhanu *et al*., 2019 [14] and Herrmann *et al*., 2000 [20].
(XLSX)

S8 Table O-glycosylation analysis of raw files of Alberthsen *et al*, 2013. Common O-glycosylated proteins (Sheet 1) and scans confirming O-glycosylated peptides (Sheet 2) identified by us in the analysis of the raw data files deposited by Albrethsen *et al*. [37].
(XLSX)

## References

[1] World Health Organization. Global Tuberculosis Report 2019. Geneva; 2019.

[2] Meena LS, Rajni. Survival mechanisms of pathogenic Mycobacterium tuberculosis H37Rv. FEBS J. 2010 6;277(11):2416–27. 10.1111/j.1742-4658.2010.07666.x 20553485

[3] Ernst JD. Mechanisms of M. tuberculosis Immune Evasion as Challenges to TB Vaccine Design. Cell Host Microbe. 2018 7;24(1):34–42. 10.1016/j.chom.2018.06.004 30001523 PMC6482466

[4] Chegou NN, Hoek KG, Kriel M, Warren RM, Victor TC, Walzl G. Tuberculosis assays: past, present and future. Expert Rev Anti Infect Ther. 2011 4 10;9(4):457–69. 10.1586/eri.11.23 21504402

[5] Steingart KR, Flores LL, Dendukuri N, Schiller I, Laal S, Ramsay A, et al Commercial serological tests for the diagnosis of active pulmonary and extrapulmonary tuberculosis: an updated systematic review and meta-analysis. PLoS Med. 2011;8(8):e1001062 10.1371/journal.pmed.1001062 21857806 PMC3153457

[6] Niederweis M, Danilchanka O, Huff J, Hoffmann C, Engelhardt H. Mycobacterial outer membranes: in search of proteins. Trends Microbiol. 2010 3;18(3):109–16. 10.1016/j.tim.2009.12.005 20060722 PMC2931330

[7] Daffé M, Etienne G. The capsule of Mycobacterium tuberculosis and its implications for pathogenicity. Tuber Lung Dis. 1999 6;79(3):153–69. 10.1054/tuld.1998.0200 10656114

[8] Bell C, Smith GT, Sweredoski MJ, Hess S. Characterization of the Mycobacterium tuberculosis Proteome by Liquid Chromatography Mass Spectrometry-based Proteomics Techniques: A Comprehensive Resource for Tuberculosis Research. J Proteome Res. 2012 1 30;11(1):119–30. 10.1021/pr2007939 22053987

[9] de Souza GA, Leversen NA, Malen H, Wiker HG. Bacterial proteins with cleaved or uncleaved signal peptides of the general secretory pathway. J Proteomics. 2011;75(2):502–10. 10.1016/j.jprot.2011.08.016 21920479

[10] van Winden VJC, Houben ENG, Braunstein M. Protein Export into and across the Atypical Diderm Cell Envelope of Mycobacteria. Microbiol Spectr. 2019 7 5;7(4). 10.1128/microbiolspec.gpp3-0043-2018 PMC10957183 31400094

[11] Solomonson M, Setiaputra D, Makepeace KAT, Lameignere E, Petrotchenko EV., Conrady DG, et al Structure of EspB from the ESX-1 type VII secretion system and insights into its export mechanism. Structure. 2015;23(3):571–83. 10.1016/j.str.2015.01.002 25684576

[12] Shah S, Briken V. Modular Organization of the ESX-5 Secretion System in Mycobacterium tuberculosis. Front Cell Infect Microbiol. 2016;6(May):1–7. 27200304 10.3389/fcimb.2016.00049 PMC4852179

[13] van Els CACM, Corbière V, Smits K, van Gaans-van den Brink JAM, Poelen MCM, Mascart F, et al Toward Understanding the Essence of Post-Translational Modifications for the Mycobacterium tuberculosis Immunoproteome. Front Immunol. 2014;5:361 10.3389/fimmu.2014.00361 25157249 PMC4127798

[14] Birhanu AG, Yimer SA, Kalayou S, Riaz T, Zegeye ED, Holm-Hansen C, et al Ample glycosylation in membrane and cell envelope proteins may explain the phenotypic diversity and virulence in the Mycobacterium tuberculosis complex. Sci Rep. 2019 12 27;9(1):2927 10.1038/s41598-019-39654-9 30814666 PMC6393673

[15] Mehaffy C, Belisle JT, Dobos KM. Mycobacteria and their sweet proteins: An overview of protein glycosylation and lipoglycosylation in M. tuberculosis. Tuberculosis. 2019;115:1–13. 10.1016/j.tube.2019.01.001 30948163

[16] VanderVen BC, Harder JD, Crick DC, Belisle JT. Export-mediated assembly of mycobacterial glycoproteins parallels eukaryotic pathways. Science (80-). 2005 8 5;309(5736):941–3. 10.1126/science.1114347 16081738

[17] Liu C-F, Tonini L, Malaga W, Beau M, Stella A, Bouyssie D, et al Bacterial protein-O-mannosylating enzyme is crucial for virulence of Mycobacterium tuberculosis. Proc Natl Acad Sci. 2013 4 16;110(16):6560–5. 10.1073/pnas.1219704110 23550160 PMC3631654

[18] González-Zamorano M, Hernández GM, Xolalpa W, Parada C, Vallecillo AJ, Bigi F, et al Mycobacterium tuberculosis glycoproteomics based on ConA-lectin affinity capture of mannosylated proteins. J Proteome Res. 2009;8(2):721–33. 10.1021/pr800756a 19196185

[19] Smith GT, Sweredoski MJ, Hess S. O-linked glycosylation sites profiling in Mycobacterium tuberculosis culture filtrate proteins. J Proteomics. 2014 1 31;97:296–306. 10.1016/j.jprot.2013.05.011 23702328 PMC3806883

[20] Herrmann JL, Delahay R, Gallagher A, Robertson B, Young D. Analysis of post-translational modification of mycobacterial proteins using a cassette expression system. FEBS Lett. 2000 5 19;473(3):358–62. 10.1016/s0014-5793(00)01553-2 10818240

[21] Mattow J, Schaible UE, Schmidt F, Hagens K, Siejak F, Brestrich G, et al Comparative proteome analysis of culture supernatant proteins from virulent Mycobacterium tuberculosis H37Rv and attenuated M. bovis BCG Copenhagen. Electrophoresis. 2003;24(19–20):3405–20. 10.1002/elps.200305601 14595687

[22] Ausubel BR, Kingston RE, Moore DD, Seidman JG, Smith JA, Struhl FM. Current protocols in molecular biology. John Wiley and Sons, New York; 1999.

[23] Steinberg TH. Chapter 31 Protein Gel Staining Methods. In 2009 p. 541–63. 10.1016/S0076-6879(09)63031-7 19892191

[24] Lima A, Duran R, Schujman GE, Marchissio MJ, Portela MM, Obal G, et al Serine/threonine protein kinase PrkA of the human pathogen Listeria monocytogenes: biochemical characterization and identification of interacting partners through proteomic approaches. J Proteomics. 2011;74(9):1720–34. 10.1016/j.jprot.2011.03.005 21406257

[25] Carvalho PC, Lima DB, Leprevost FV, Santos MDM, Fischer JSG, Aquino PF, et al PatternLab for proteomics 4.0: A one-stop shop for analyzing shotgun proteomic data. Nat Protoc. 2016 1 10;11(1):102–17. 10.1038/nprot.2015.133 26658470 PMC5722229

[26] Eng JK, Hoopmann MR, Jahan TA, Egertson JD, Noble WS, MacCoss MJ. A Deeper Look into Comet—Implementation and Features. J Am Soc Mass Spectrom. 2015 11 27;26(11):1865–74. 10.1007/s13361-015-1179-x 26115965 PMC4607604

[27] Carvalho PC, Fischer JSG, Xu T, Cociorva D, Balbuena TS, Valente RH, et al Search engine processor: Filtering and organizing peptide spectrum matches. Proteomics. 2012 4 1;12(7):944–9. 10.1002/pmic.201100529 22311825 PMC3334471

[28] Zhang B, Chambers MC, Tabb DL. Proteomic Parsimony through Bipartite Graph Analysis Improves Accuracy and Transparency. J Proteome Res. 2007 9;6(9):3549–57. 10.1021/pr070230d 17676885 PMC2810678

[29] Hulsen T, de Vlieg J, Alkema W. BioVenn—a web application for the comparison and visualization of biological lists using area-proportional Venn diagrams. BMC Genomics. 2008 10 16;9(1):488. 18925949 10.1186/1471-2164-9-488 PMC2584113

[30] UniProt Consortium T. UniProt: the universal protein knowledgebase. Nucleic Acids Res. 2018 3 16;46(5):2699–2699. 10.1093/nar/gky092 29425356 PMC5861450

[31] Kapopoulou A, Lew JM, Cole ST. The MycoBrowser portal: A comprehensive and manually annotated resource for mycobacterial genomes. Tuberculosis. 2011 1;91(1):8–13. 10.1016/j.tube.2010.09.006 20980200

[32] de Fischer JSdG, dos Santos MDM, Marchini FK, Barbosa VC, Carvalho PC, Zanchin NIT. A scoring model for phosphopeptide site localization and its impact on the question of whether to use MSA. J Proteomics. 2014;129:42–50. 10.1016/j.jprot.2015.01.008 25623781

[33] Almagro Armenteros JJ, Tsirigos KD, Sønderby CK, Petersen TN, Winther O, Brunak S, et al SignalP 5.0 improves signal peptide predictions using deep neural networks. Nat Biotechnol. 2019 2 18; 10.1038/s41587-019-0036-z 30778233

[34] McIlwain S, Mathews M, Bereman MS, Rubel EW, MacCoss MJ, Noble WS. Estimating relative abundances of proteins from shotgun proteomics data. BMC Bioinformatics. 2012;13:308 10.1186/1471-2105-13-308 23164367 PMC3599300

[35] Sudha D, Kohansal-Nodehi M, Kovuri P, Manda SS, Neriyanuri S, Gopal L, et al Proteomic profiling of human intraschisis cavity fluid. Clin Proteomics. 2017;14(1):1–12. 28450823 10.1186/s12014-017-9148-y PMC5404285

[36] Malen H, Berven FS, Fladmark KE, Wiker HG. Comprehensive analysis of exported proteins from Mycobacterium tuberculosis H37Rv. Proteomics. 2007;7(10):1702–18. 10.1002/pmic.200600853 17443846

[37] Albrethsen J, Agner J, Piersma SR, Højrup P, Pham T V., Weldingh K, et al Proteomic Profiling of Mycobacterium tuberculosis Identifies Nutrient-starvation-responsive Toxin–antitoxin Systems. Mol Cell Proteomics. 2013 5;12(5):1180–91. 10.1074/mcp.M112.018846 23345537 PMC3650330

[38] Oliveros JC. Venny. An interactive tool for comparing lists with Venn’s diagrams.

[39] Huang DW, Sherman BT, Lempicki RA. Bioinformatics enrichment tools: paths toward the comprehensive functional analysis of large gene lists. Nucleic Acids Res. 2009 1;37(1):1–13. 10.1093/nar/gkn923 19033363 PMC2615629

[40] Huang DW, Sherman BT, Lempicki RA. Systematic and integrative analysis of large gene lists using DAVID bioinformatics resources. Nat Protoc. 2009 1 1;4(1):44–57. 10.1038/nprot.2008.211 19131956

[41] Perkins DN, Pappin DJC, Creasy DM, Cottrell JS. Probability-based protein identification by searching sequence databases using mass spectrometry data. Electrophoresis. 1999 12 1;20(18):3551–67. 10.1002/(SICI)1522-2683(19991201)20:18<3551::AID-ELPS3551>3.0.CO;2-2 10612281

[42] Wang M, You J, Bemis KG, Tegeler TJ, Brown DPG. Label-free mass spectrometry-based protein quantification technologies in proteomic analysis. Briefings Funct Genomics Proteomics. 2008 6 25;7(5):329–39. 10.1093/bfgp/eln031 18579615

[43] Zybailov B, Mosley AL, Sardiu ME, Coleman MK, Florens L, Washburn MP. Statistical Analysis of Membrane Proteome Expression Changes in Saccharomyces cerevisiae. J Proteome Res. 2006 9;5(9):2339–47. 10.1021/pr060161n 16944946

[44] Forrellad MA, Klepp LI, Gioffré A, Sabio García J, Morbidoni HR, de la Paz Santangelo M, et al Virulence factors of the Mycobacterium tuberculosis complex. Virulence. 2013;4(1):3–66. 10.4161/viru.22329 23076359 PMC3544749

[45] Nambi S, Long JE, Mishra BB, Baker R, Murphy KC, Olive AJ, et al The Oxidative Stress Network of Mycobacterium tuberculosis Reveals Coordination between Radical Detoxification Systems. Cell Host Microbe. 2015 6 10;17(6):829–37. 10.1016/j.chom.2015.05.008 26067605 PMC4465913

[46] Tucci P, González-Sapienza G, Marin M. Pathogen-derived biomarkers for active tuberculosis diagnosis. Front Microbiol. 2014;5(OCT). 10.3389/fmicb.2014.00549 PMC4202705 25368609

[47] Stanley SA, Raghavan S, Hwang WW, Cox JS. Acute infection and macrophage subversion by Mycobacterium tuberculosis require a specialized secretion system. Proc Natl Acad Sci. 2003 10 28;100(22):13001–6. 10.1073/pnas.2235593100 14557536 PMC240734

[48] Abdallah AM, Vandenbroucke-Grauls CMJE, Luirink J, Gey van Pittius NC, Cox J, Appelmelk BJ, et al Type VII secretion—mycobacteria show the way. Nat Rev Microbiol. 2007;5(11):883–91. 10.1038/nrmicro1773 17922044

[49] Cole ST, Brosch R, Parkhill J, Garnier T, Churcher C, Harris D, et al Deciphering the biology of Mycobacterium tuberculosis from the complete genome sequence. Nature. 1998;393(6685):537–44. 10.1038/31159 9634230

[50] Korotkova N, Freire D, Phan TH, Ummels R, Creekmore CC, Evans TJ, et al Structure of the Mycobacterium tuberculosis type VII secretion system chaperone EspG 5 in complex with PE25-PPE41 dimer. Mol Microbiol. 2014;94(2):367–82. 10.1111/mmi.12770 25155747 PMC4192059

[51] Andersen P, Askgaard D, Ljungqvist L, Bennedsen J, Heron I. Proteins released from Mycobacterium tuberculosis during growth. Infect Immun. 1991;59(6):1905–10. 1903768 10.1128/iai.59.6.1905-1910.1991 PMC257941

[52] Tullius M V., Harth G, Horwitz MA. High extracellular levels of Mycobacterium tuberculosis glutamine synthetase and superoxide dismutase in actively growing cultures are due to high expression and extracellular stability rather than to a protein-specific export mechanism. Infect Immun. 2001;69(10):6348–63. 10.1128/IAI.69.10.6348-6363.2001 11553579 PMC98770

[53] Schubert OT, Ludwig C, Kogadeeva M, Zimmermann M, Rosenberger G, Gengenbacher M, et al Absolute proteome composition and dynamics during dormancy and resuscitation of mycobacterium tuberculosis. Cell Host Microbe. 2015;18(1):96–108. 10.1016/j.chom.2015.06.001 26094805

[54] Tiwari P, Arora G, Singh M, Kidwai S, Narayan OP, Singh R. MazF ribonucleases promote Mycobacterium tuberculosis drug tolerance and virulence in guinea pigs. Nat Commun. 2015 5 22;6(1):6059. 25608501 10.1038/ncomms7059

[55] Huang Y, Ge J, Yao Y, Wang Q, Shen H, Wang H. Characterization and site-directed mutagenesis of the putative novel acyl carrier protein Rv0033 and Rv1344 from Mycobacterium tuberculosis. Biochem Biophys Res Commun. 2006;342(2):618–24. 10.1016/j.bbrc.2006.01.178 16487939

[56] Dobos KM, Swiderek K, Khoo KH, Brennan PJ, Belisle JT. Evidence for glycosylation sites on the 45-kilodalton glycoprotein of Mycobacterium tuberculosis. Infect Immun. 1995 8;63(8):2846–53. 7622204 10.1128/iai.63.8.2846-2853.1995 PMC173386

[57] Dobos KM, Khoo KH, Swiderek KM, Brennan PJ, Belisle JT. Definition of the full extent of glycosylation of the 45-kilodalton glycoprotein of Mycobacterium tuberculosis. J Bacteriol. 1996 5;178(9):2498–506. 10.1128/jb.178.9.2498-2506.1996 8626314 PMC177971

[58] Alonso H, Parra J, Malaga W, Payros D, Liu C-F, Berrone C, et al Protein O-mannosylation deficiency increases LprG-associated lipoarabinomannan release by Mycobacterium tuberculosis and enhances the TLR2-associated inflammatory response. Sci Rep. 2017 12 11;7(1):7913 10.1038/s41598-017-08489-7 28801649 PMC5554173

[59] Ragas A, Roussel L, Puzo G, Rivière M. The Mycobacterium tuberculosis Cell-surface Glycoprotein Apa as a Potential Adhesin to Colonize Target Cells via the Innate Immune System Pulmonary C-type Lectin Surfactant Protein A. J Biol Chem. 2007 2 23;282(8):5133–42. 10.1074/jbc.M610183200 17158455

[60] Diaz-Silvestre H, Espinosa-Cueto P, Sanchez-Gonzalez A, Esparza-Ceron MA, Pereira-Suarez AL, Bernal-Fernandez G, et al The 19-kDa antigen of Mycobacterium tuberculosis is a major adhesin that binds the mannose receptor of THP-1 monocytic cells and promotes phagocytosis of mycobacteria. Microb Pathog. 2005 9;39(3):97–107. 10.1016/j.micpath.2005.06.002 16098710

[61] Keenan T, Dowle A, Bates R, Smith MCM. Characterization of the Streptomyces coelicolor Glycoproteome Reveals Glycoproteins Important for Cell Wall Biogenesis. 2019; 10.1128/mBio.01092-19 PMC6593405 31239379

[62] Dheda K, Barry CE, Maartens G. Tuberculosis Vol. 387, The Lancet. Lancet Publishing Group; 2016 p. 1211–26. 10.1016/S0140-6736(15)00151-8 PMC11268880 26377143

[63] Parra J, Marcoux J, Poncin I, Canaan S, Herrmann JL, Nigou J, et al Scrutiny of Mycobacterium tuberculosis 19 kDa antigen proteoforms provides new insights in the lipoglycoprotein biogenesis paradigm. Sci Rep. 2017 3 8;7. 10.1038/srep43682 PMC5341126 28272507

[64] Garbe T, Harris D, Vordermeier M, Lathigra R, Ivanyi J, Young D. Expression of the Mycobacterium tuberculosis 19-kilodalton antigen in Mycobacterium smegmatis: immunological analysis and evidence of glycosylation. Infect Immun. 1993 1;61(1):260–7. 8418047 10.1128/iai.61.1.260-267.1993 PMC302713

[65] Michell SL, Whelan AO, Wheeler PR, Panico M, Easton RL, Etienne AT, et al The MPB83 antigen from Mycobacterium bovis contains O-linked mannose and (1—>3)-mannobiose moieties. J Biol Chem. 2003 5 2;278(18):16423–32. 10.1074/jbc.M207959200 12517764

[66] Bando-Campos G, Juárez-López D, Román-González SA, Castillo-Rodal AI, Olvera C, López-Vidal Y, et al Recombinant O-mannosylated protein production (PstS-1) from Mycobacterium tuberculosis in Pichia pastoris (Komagataella phaffii) as a tool to study tuberculosis infection. Microb Cell Fact. 2019 12 19;18(1):11 10.1186/s12934-019-1059-3 30660186 PMC6339365

[67] Kumar A, Toledo JC, Patel RP, Lancaster JR, Steyn AJC. Mycobacterium tuberculosis DosS is a redox sensor and DosT is a hypoxia sensor. Proc Natl Acad Sci. 2007 7 10;104(28):11568–73. 10.1073/pnas.0705054104 17609369 PMC1906723

[68] Sivaramakrishnan S, De Montellano PRO. The DosS-DosT/DosR Mycobacterial Sensor System Vol. 3, Biosensors. MDPI AG; 2013 p. 259–82. 10.3390/bios3030259 PMC4082495 25002970

[69] Leistikow RL, Morton RA, Bartek IL, Frimpong I, Wagner K, Voskuil MI. The Mycobacterium tuberculosis DosR Regulon Assists in Metabolic Homeostasis and Enables Rapid Recovery from Nonrespiring Dormancy. J Bacteriol. 2010 3 15;192(6):1662–70. 10.1128/JB.00926-09 20023019 PMC2832541

[70] Hatherill M, Tait D, McShane H. Clinical Testing of Tuberculosis Vaccine Candidates In: Tuberculosis and the Tubercle Bacillus, Second Edition American Society of Microbiology; 2016 p. 193–211. 10.1128/microbiolspec.TBTB2-0015-2016 28087924

[71] Khoshnood S, Heidary M, Haeili M, Drancourt M, Darban-Sarokhalil D, Nasiri MJ, et al Novel vaccine candidates against Mycobacterium tuberculosis. Int J Biol Macromol. 2018 12;120:180–8. 10.1016/j.ijbiomac.2018.08.037 30098365

[72] Nielsen H. Predicting secretory proteins with signaIP In: Methods in Molecular Biology. 2017 p. 59–73. 10.1007/978-1-4939-7015-5_6 28451972

[73] Kovacs-Simon A, Titball RW, Michell SL. Lipoproteins of Bacterial Pathogens. Infect Immun. 2011 2;79(2):548 10.1128/IAI.00682-10 20974828 PMC3028857

[74] York IA, Stevens J, Alymova IV. Influenza virus N-linked glycosylation and innate immunity. Biosci Rep. 2019 1 31;39(1):BSR20171505. 10.1042/BSR20171505 PMC6328934 30552137

[75] Børud B, Bårnes GK, Brynildsrud OB, Fritzsønn E, Caugant DA. Genotypic and Phenotypic Characterization of the O-Linked Protein Glycosylation System Reveals High Glycan Diversity in Paired Meningococcal Carriage Isolates. J Bacteriol. 2018 8 15;200(16):e00794–17. 10.1128/JB.00794-17 29555702 PMC6060354

