# Supplementary material for: Integrative proteomic and glycoproteomic profiling of Mycobacterium tuberculosis culture filtrate
Source: PLoS One. 2020 Mar 3;15(3):e0221837. doi: 10.1371/journal.pone.0221837 (PMC7053730; doi:10.1371/journal.pone.0221837)
Supplement: S1 Raw images — (PDF) [file pone.0221837.s001.pdf]

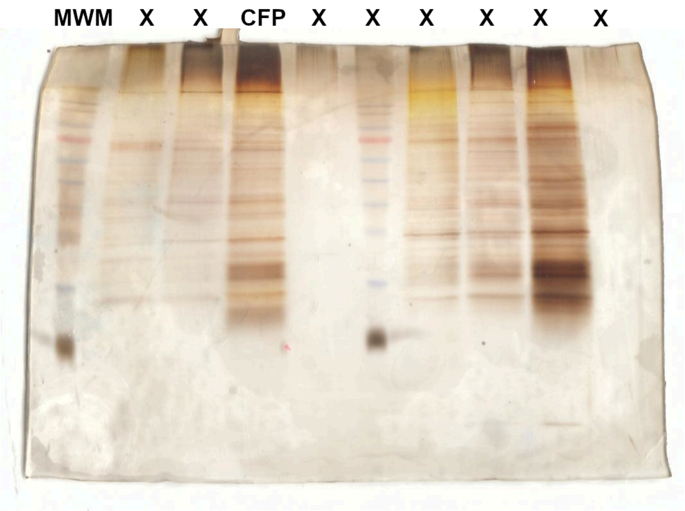

S1A Original Gel Image

MWM: Molecular weight marker (Thermo Fischer Scientific, # 26616).

CFP: M. tuberculosis culture filtrate proteins (CFP).

Method used to capture the image: Desktop Image Scanner

Staining method: Silver nitrate

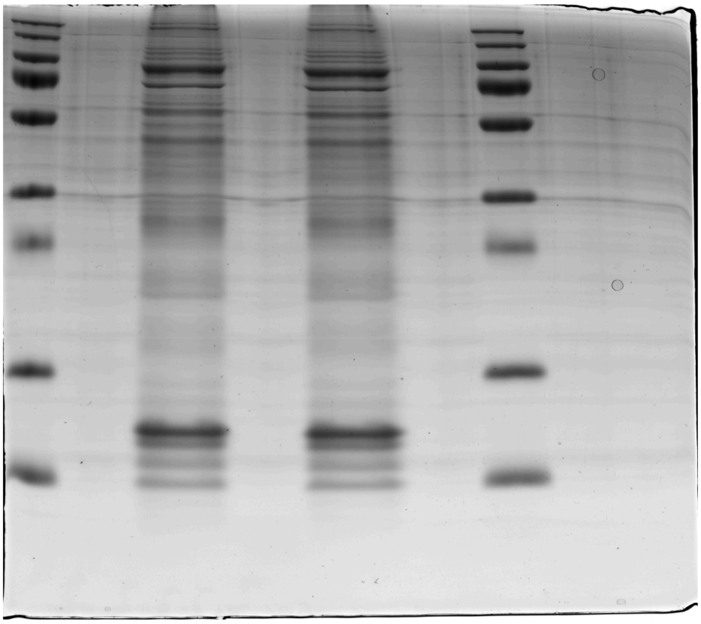

S1B Original Gel Image

MWM: Molecular weight marker (Thermo Fischer Scientific, # 26616).
CFP(1) and CFP(2): Technical replicates of M. tuberculosis culture filtrate proteins.
Method used to capture the image: Desktop Image Scanner
Staining method: CBB G-250
