# Supplementary figures and images for: Integrative proteomic and glycoproteomic profiling of Mycobacterium tuberculosis culture filtrate
Source: PLoS One. 2020 Mar 3;15(3):e0221837. doi: 10.1371/journal.pone.0221837 (PMC7053730; doi:10.1371/journal.pone.0221837)

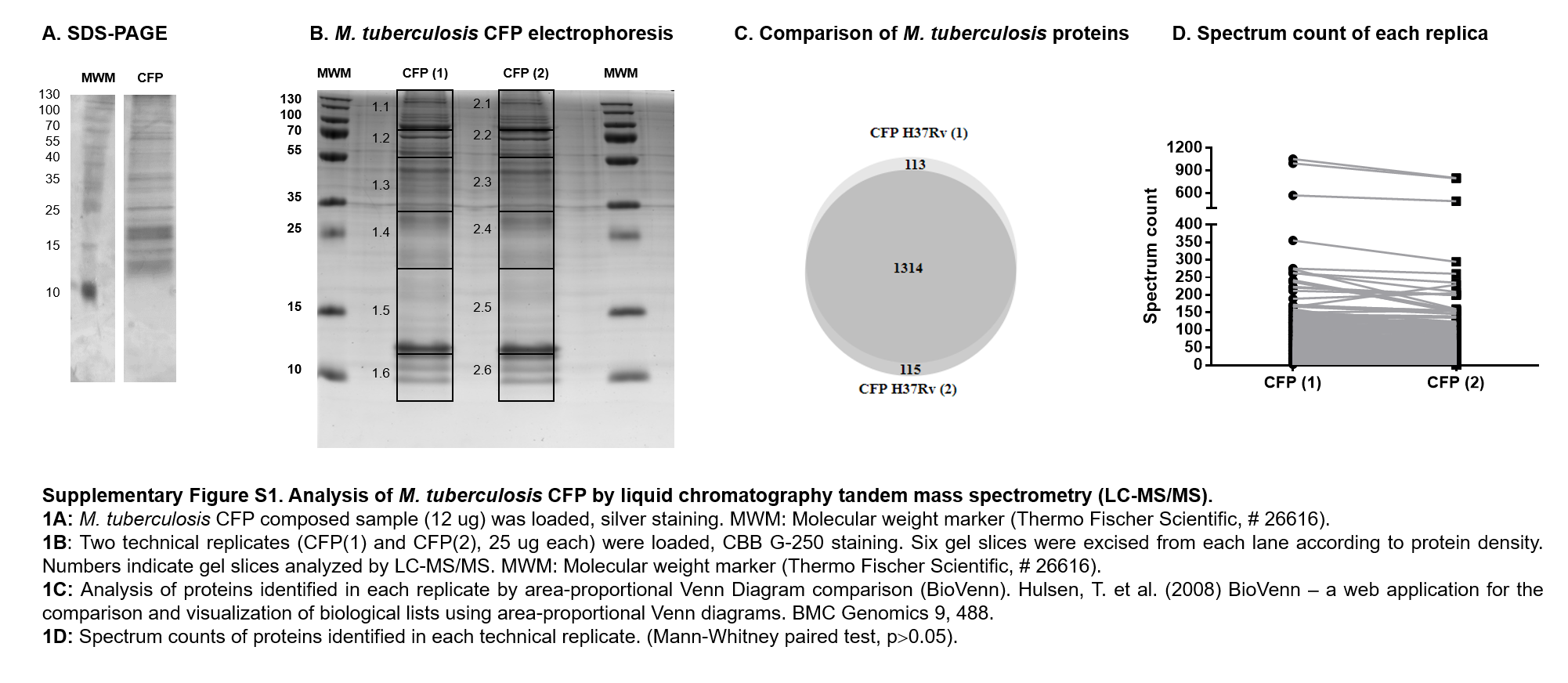

Supplement: S1 Fig — S1A: M. tuberculosis CFP analysis by 1D SDS-PAGE 15% and silver nitrate staining. S1B: M. tuberculosis CFP analysis by 1D SDS-PAGE 15% and CCB G-250 staining. S1C: Spectrum counts of proteins identified in each technical replicate. S1D: Analysis of proteins identified in each replicate by area-proportional Venn Diagram comparison [29]. (TIF) [file pone.0221837.s002.tif]

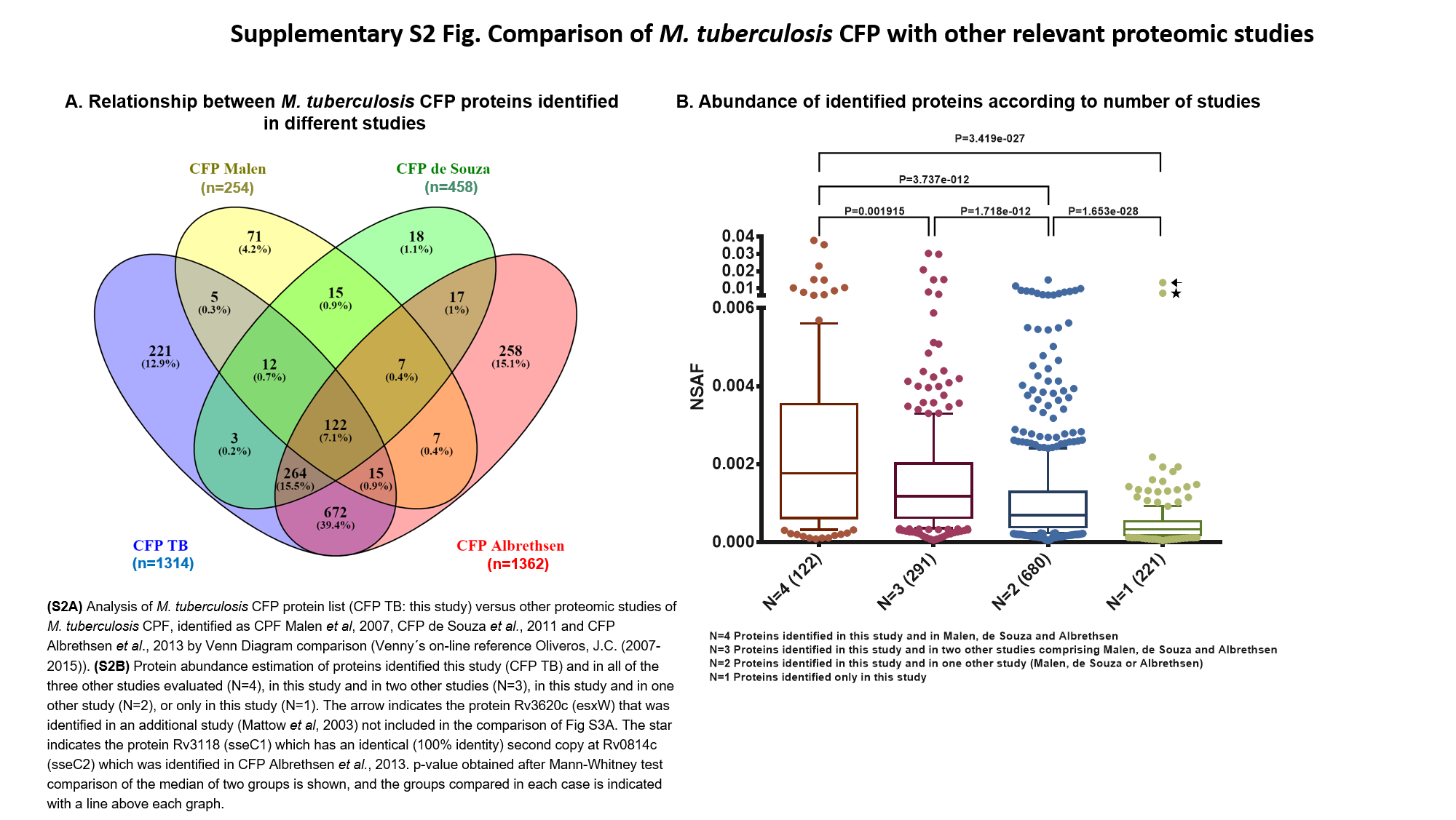

Supplement: S2 Fig — S2A: Analysis of M. tuberculosis CFP protein list (CFP TB: this study) versus other proteomic studies of M. tuberculosis CPF. S2B: Protein abundance estimation of proteins identified this study (CFP TB) and in all of the three other studies evaluated (N = 4), in this study and in two other studies (N = 3), in this study and in one other study (N = 2), or only in this study (N = 1). (TIF) [file pone.0221837.s003.tif]
