# Supplementary material for: Integrative proteomic and glycoproteomic profiling of Mycobacterium tuberculosis culture filtrate
Source: PLoS One. 2020 Mar 3;15(3):e0221837. doi: 10.1371/journal.pone.0221837 (PMC7053730; doi:10.1371/journal.pone.0221837)

MazF7 (Rv2063A) Possible toxin MazF7

File Statistics Tools

Control Follow up Result Browser

Spec FDR: 0 / 0 = NaN% Pep FDR: 0 / 0 = NaN% Prot FDR: 0 / 0 = NaN% # Prot (Max Parsimony): 1635 Unique Prot: 1634 Unlabeled Decoys: 0 / 0

View mode: ○ Proteins ◉ Proteins Max Pars ○ Peptides ○ Scans mazf7

	Locus	Group	GroupType	Length	#UniquePeptides	MolWt	SequenceCount	SpectrumCount	NSAF	Coverage	Protein Score	Description
1	P0CL62	958	Unique	136	6	14208.7	6	9	0.000572660478457528	0.7426	26.2139	Probable endoribonuclease MazF7 OS=Mycobacterium tuberculosis (strain ATCC 25618 / H37Rv) GN=mazF7 PE=1 SV=1

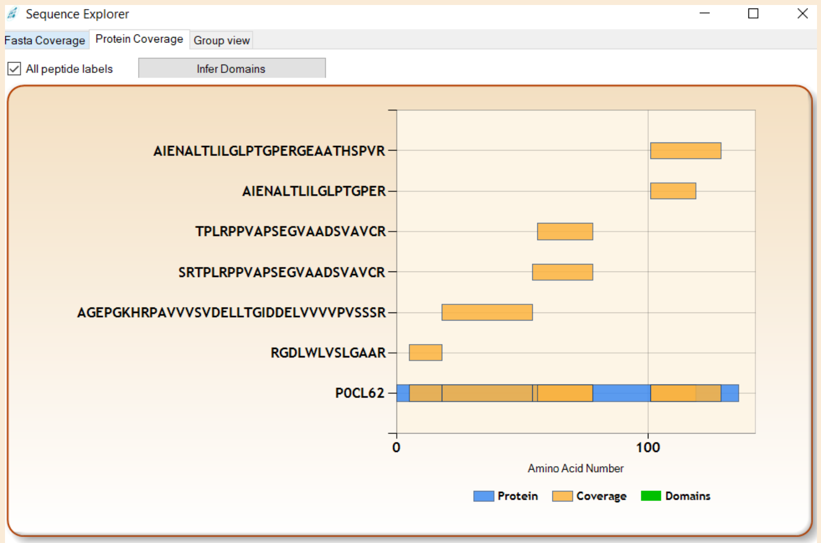

	File Name	Scan No	Z	Unique	MeasuredMH	TheoreticalMH	PPM	PrimaryScore	SecondaryScore	DeltaCN	PeaksMatched	BayesianScore	PeptideSequence
1	171213_PT_H37Rv_1-5.sqt	13269	3	True	2250.169346	2249.170674	-2.07895	3.4005	10.5847061622308	0.5406	25	0.53012	R.TPLRPPVAPSEGVAADSVAVCR.G
2	171213_PT_H37Rv_1-5.sqt	23449	4	True	2884.563864	2883.568682	-2.83162	3.1984	8.36447210439857	0.5229	33	0.51318	R.AIENALTLILGLPTGPERGEAATHSPVR.W
3	171213_PT_H37Rv_1-5.sqt	20081	2	True	1413.797537	1413.796109	1.01005	3.4682	7.93777477618463	0.4941	19	0.50783	R.RGDLWLVSLGAAR.A
4	171213_PT_H37Rv_1-5.sqt	27111	3	True	1878.06699	1878.06949	-1.33116	3.1224	7.56360667479839	0.4876	24	0.49616	R.AIENALTLILGLPTGPER.G
5	171213_PT_H37Rv_1-5.sqt	20067	3	True	1413.793899	1413.796109	-1.56317	2.9076	6.08797544748883	0.4237	22	0.45541	R.RGDLWLVSLGAAR.A
6	171213_PT_H37Rv_1-5.sqt	26207	4	True	3726.988669	3726.007231	-5.87928	2.6753	5.17787121347217	0.4821	25	0.45319	R.AGEPGKHRPAVVVSVDELLTGIDDELVVVVPVSSSR.S
7	171213_PT_H37Rv_1-5.sqt	11824	4	True	2492.295193	2492.303813	-3.45866	3.1656	4.45675018086982	0.3782	39	0.41881	R.SRTPLRPPVAPSEGVAADSVAVCR.G
8	171213_PT_H37Rv_1-5.sqt	23502	3	True	2884.564427	2883.568682	-2.63645	1.9313	1.78379129957888	0.3476	18	0.39683	R.AIENALTLILGLPTGPERGEAATHSPVR.W
9	171213_PT_H37Rv_1-5.sqt	27125	2	True	1878.061626	1878.06949	-4.1873	2.3446	4.21990778519745	0.3623	17	0.39378	R.AIENALTLILGLPTGPER.G

>Mycobacterium tuberculosis H37Rv|Rv2063A|mazF7
LAEPR**RGDLWLVSLGAAR**AGEPGKHRPAVVVSVDELLTGIDDELVVVVPVSSSRSRTPLRPPVAPSEGVAADSVAVCRGVRAVARARLVERLGALKPATMR**AIENALTLILGLPTGPER**GEAATHSPVRWTGGRDP

In bold and red: Peptide spectra shown in next slides

MazF7 (Rv2063A) Possible toxin MazF7

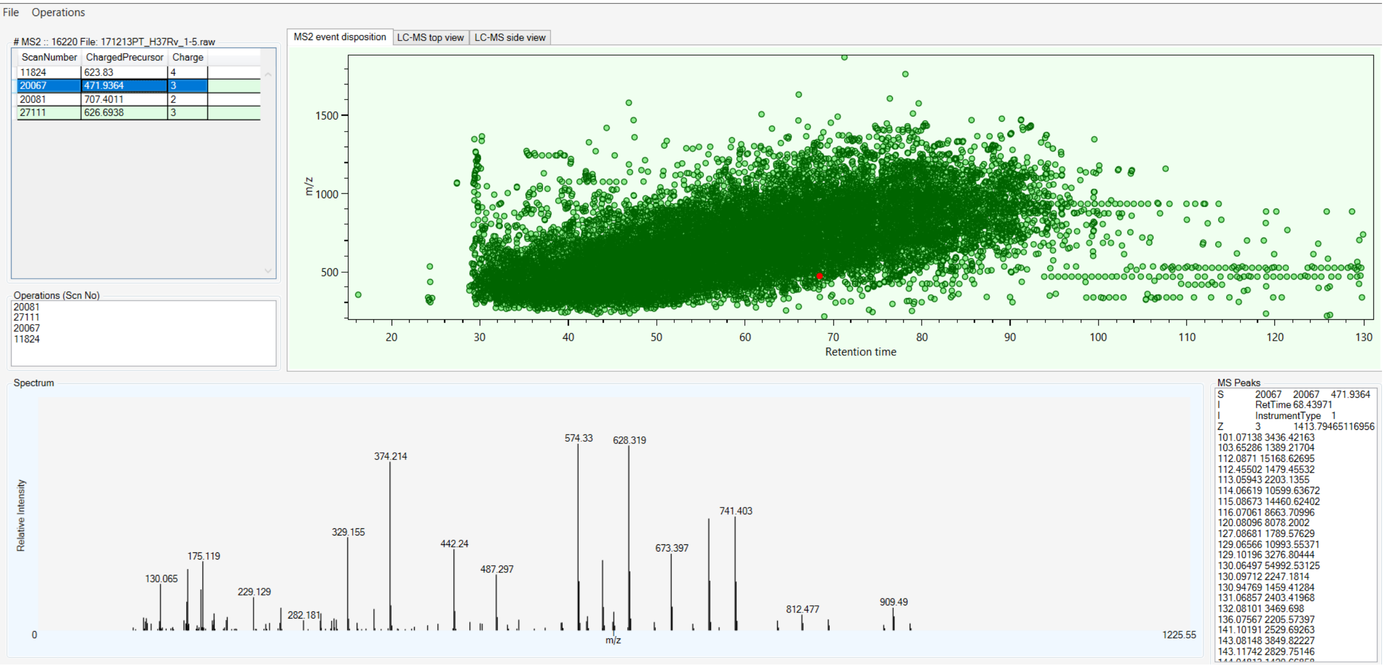

Peptide: RGDLWLVSLGAAR

MS/MS Fragmentation of **RGDLWLVSLGAAR**
Found in **WP_003410654.1** in **NCBIprot**, MULTISPECIES: mRNA interferase MazF7 [Mycobacterium]

Match to Query 1: 1412.787375 from(1413.794651,1+) index(2)
Data file Scans MazF7.txt

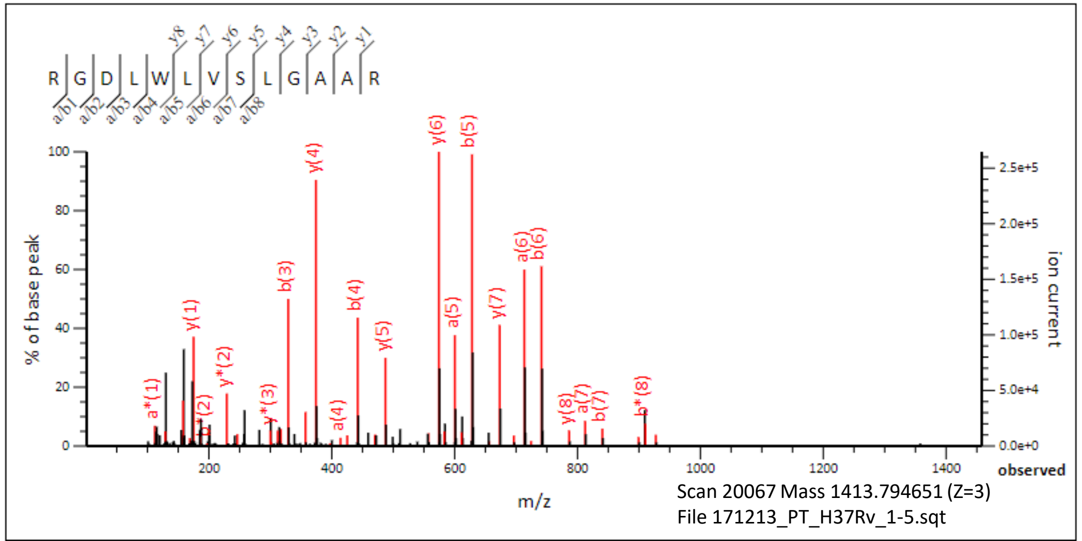

MazF7 (Rv2063A) Possible toxin MazF7

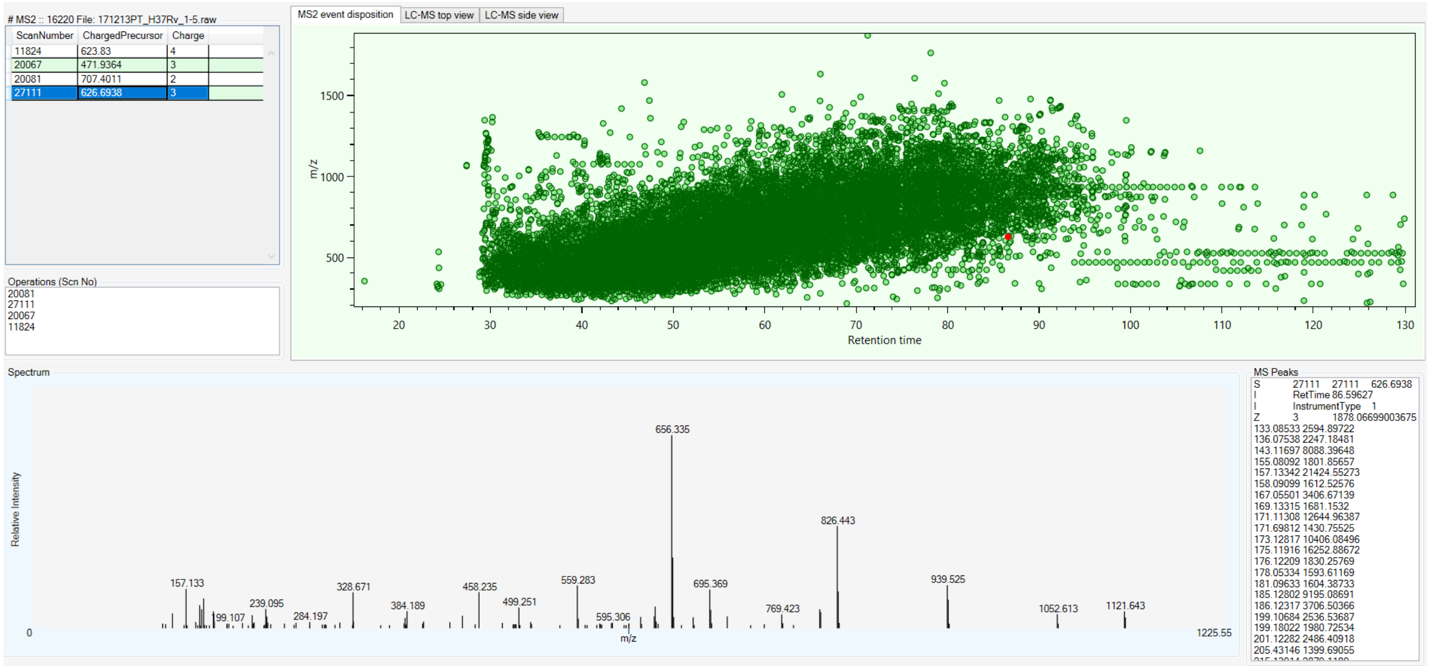

Peptide: AIENALTLILGLPTGPER

MS/MS Fragmentation of **AIENALTLILGLPTGPER**
Found in **WP_003410654.1** in **NCBIprot**, MULTISPECIES: mRNA interferase MazF7 [Mycobacterium]

Match to Query 3: 1877.059714 from(1878.066990,1+) index(4)
Data file Scans MazF7.txt

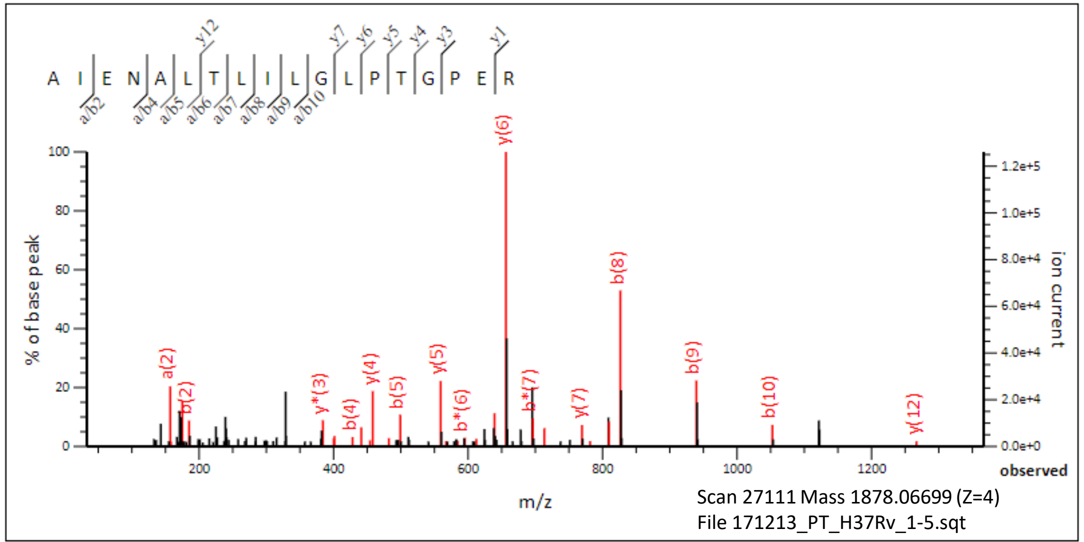

MbtL (Rv1344) Acyl carrier protein (ACP) MbtL

File Statistics Tools

Control Follow up Result Browser

Spec FDR: 0 / 0 = NaN% Pep FDR: 0 / 0 = NaN% Prot FDR: 0 / 0 = NaN% # Prot (Max Parsimony): 1642 Unique Prot: 1643 Unlabeled Decoys: 0 / 0

View mode: (•) Proteins ( ) Proteins Max Pars ( ) Peptides ( ) Scans mbtl

	Locus	Group	GroupType	Length	#UniquePeptides	MolWt	SequenceCount	SpectrumCount	NSAF	Coverage	Protein Score	Description
1	P9WQF1	890	Unique	84	4	8945.5	4	5	0.000556871453134169	0.869	12.5988	Acyl carrier protein MbtL OS=Mycobacterium tuberculosis (strain ATCC 25618 / H37Rv) GN=mbtL PE=1 SV=1

Sequence Explorer

Fasta Coverage | Protein Coverage | Group view

[x] All peptide labels   Infer Domains

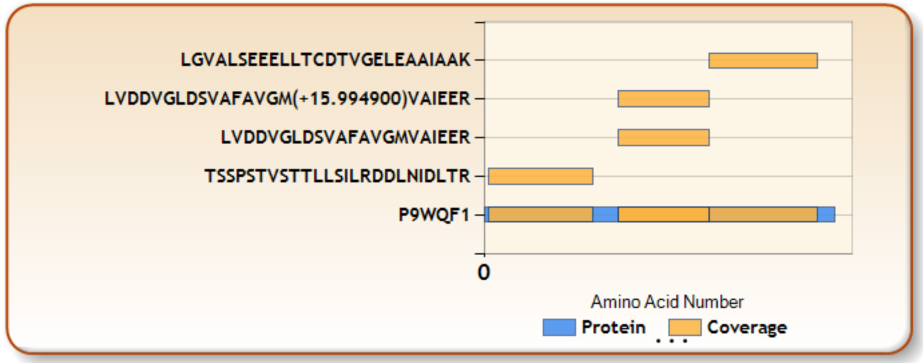

	File Name	Scan No	Z	Unique	MeasuredMH	TheoreticalMH	PPM	PrimaryScore	SecondaryScore	DeltaCN	PeaksMatched	BayesianScore	PeptideSequence
1	171213_PT_H37Rv_2-6.sqt	24157	3	True	2719.449803	2718.451981	-2.03276	3.3616	6.40093767661368	0.4721	32	0.79142	M.TSSPSTVSTTLLSILRDDLNIDLTR.V
2	171213_PT_H37Rv_2-6.sqt	24052	3	True	2703.379917	2702.380451	-1.43672	2.4312	4.66810998576197	0.5038	18	0.46976	R.LGVALSEEELLTCDTVGELEAAIAAK.Y
3	171213_PT_H37Rv_2-6.sqt	24412	3	True	2306.182957	2305.174425	2.247	2.4455	6.57845153183954	0.4686	19	0.44444	R.LVDDVGLDSVAFAVGMVAIEER.L
4	171213_PT_H37Rv_2-6.sqt	24067	3	True	2703.37723	2702.380451	-2.43066	2.4948	6.96644427533082	0.4436	22	0.42201	R.LGVALSEEELLTCDTVGELEAAIAAK.Y
5	171213_PT_H37Rv_2-6.sqt	22115	3	True	2321.169416	2321.169325	0.0392	1.8657	2.46275384514687	0.3764	19	0.3838	R.LVDDVGLDSVAFAVGM(+15.994900)VAIEER.L

>Mycobacterium tuberculosis H37Rv|Rv1344|mbtL
MWRYPLSTRLALPNTPGVASFAMTSSPSTVSTTLLSILRDDLNIDLTRVTPDAR**LVDDVGLDSVAFAVGMVAIEER****LGVALSEEELLTCDTVGELEAAIAAK**YRDE

In bold, red and green: Peptide spectra shown in next slides

MbtL (Rv1344) Acyl carrier protein (ACP) MbtL

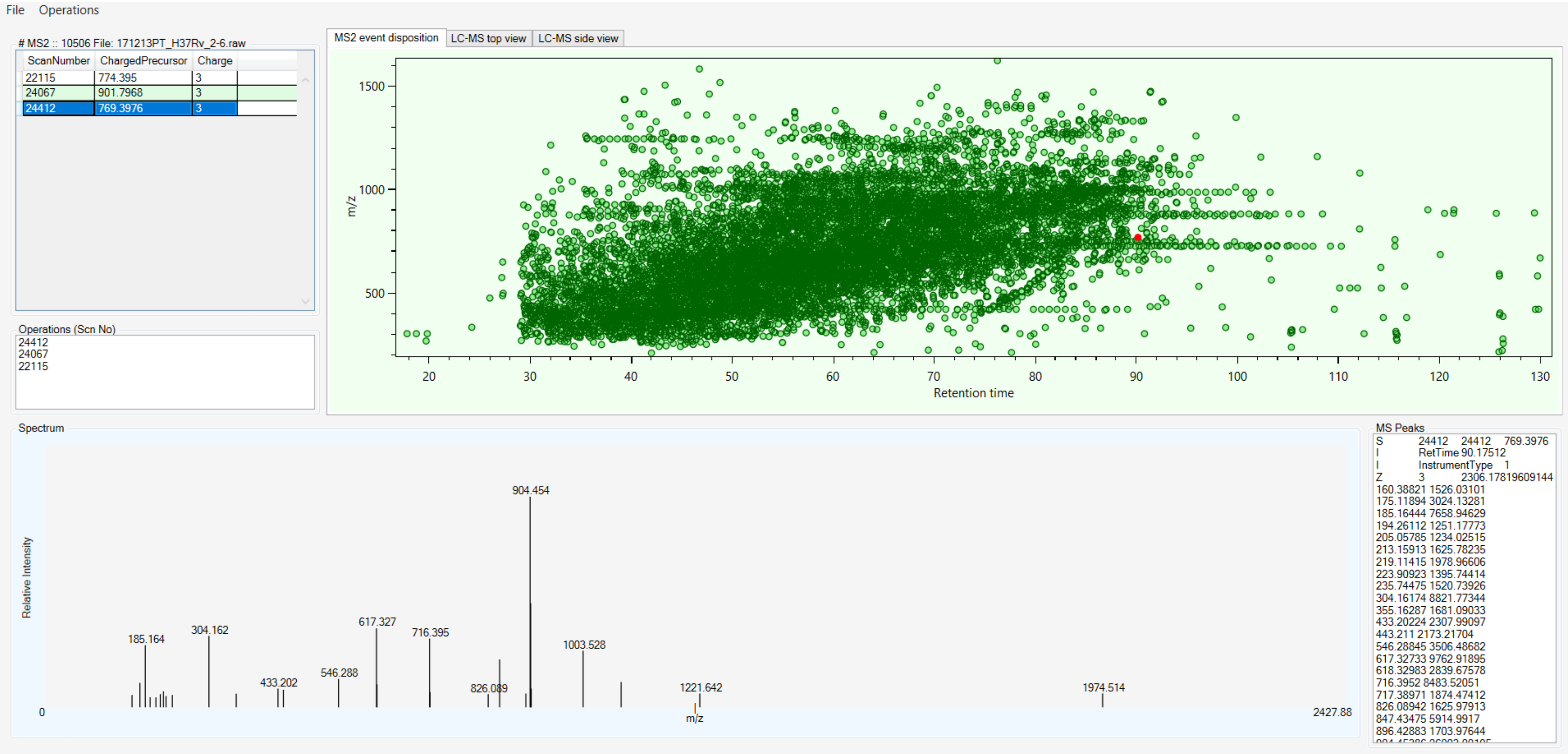

Peptide:

R.LVDDVGLDSVAFAVGMVAIEER.L

MS/MS Fragmentation of **LVDDVGLDSVAFAVGMVAIEER**
Found in **Q73ZP6.1** in **NCBIprot**, RecName: Full=Acyl carrier protein MbtL; Short=ACP; AltName: Full=Mycobactin synthase protein L

Match to Query 1: 2304.170920 from(2305.178196,1+) index(2)
Data file Scans MbtL.txt

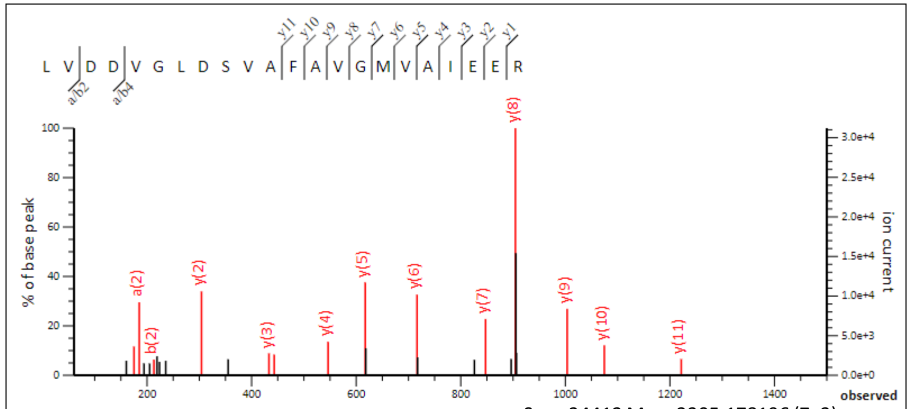

Scan 24412 Mass 2305.178196 (Z=3)
File 171213_PT_H37Rv_2-6.sqt

```
Monoisotopic mass of neutral peptide Mr(calc): 2304.1672
Fixed modifications: Carboxymethyl (C) (apply to specified residues or termini only)
Ions Score: 94  Expect: 3.5e-05   (help)
```

#	a	b	Seq.	y	y*	#
1	86.0964	114.0913	L			22
2	**185.1648**	**213.1598**	V	2192.0904	2175.0638	21
3	300.1918	328.1867	D	2093.0219	2075.9954	20
4	415.2187	**443.2136**	D	1977.9950	1960.9685	19
5	514.2871	542.2821	V	1862.9681	1845.9415	18
6	571.3086	599.3035	G	1763.8996	1746.8731	17
7	684.3927	712.3876	L	1706.8782	1689.8516	16
8	799.4196	827.4145	D	1593.7941	1576.7676	15
9	886.4516	914.4466	S	1478.7672	1461.7406	14
10	985.5201	1013.5150	V	1391.7351	1374.7086	13
11	1056.5572	1084.5521	A	1292.6667	1275.6402	12
12	1203.6256	1231.6205	F	**1221.6296**	1204.6031	11
13	1274.6627	1302.6576	A	**1074.5612**	1057.5347	10
14	1373.7311	1401.7260	V	**1003.5241**	986.4975	9
15	1430.7526	1458.7475	G	**904.4557**	887.4291	8
16	1561.7931	1589.7880	M	**847.4342**	830.4077	7
17	1660.8615	1688.8564	V	**716.3937**	699.3672	6
18	1731.8986	1759.8935	A	**617.3253**	600.2988	5
19	1844.9826	1872.9776	I	**546.2882**	529.2617	4
20	1974.0252	2002.0202	E	**433.2041**	416.1776	3
21	2103.0678	2131.0628	E	**304.1615**	287.1350	2
22			R	**175.1190**	158.0924	1

MbtL (Rv1344) Acyl carrier protein (ACP) MbtL

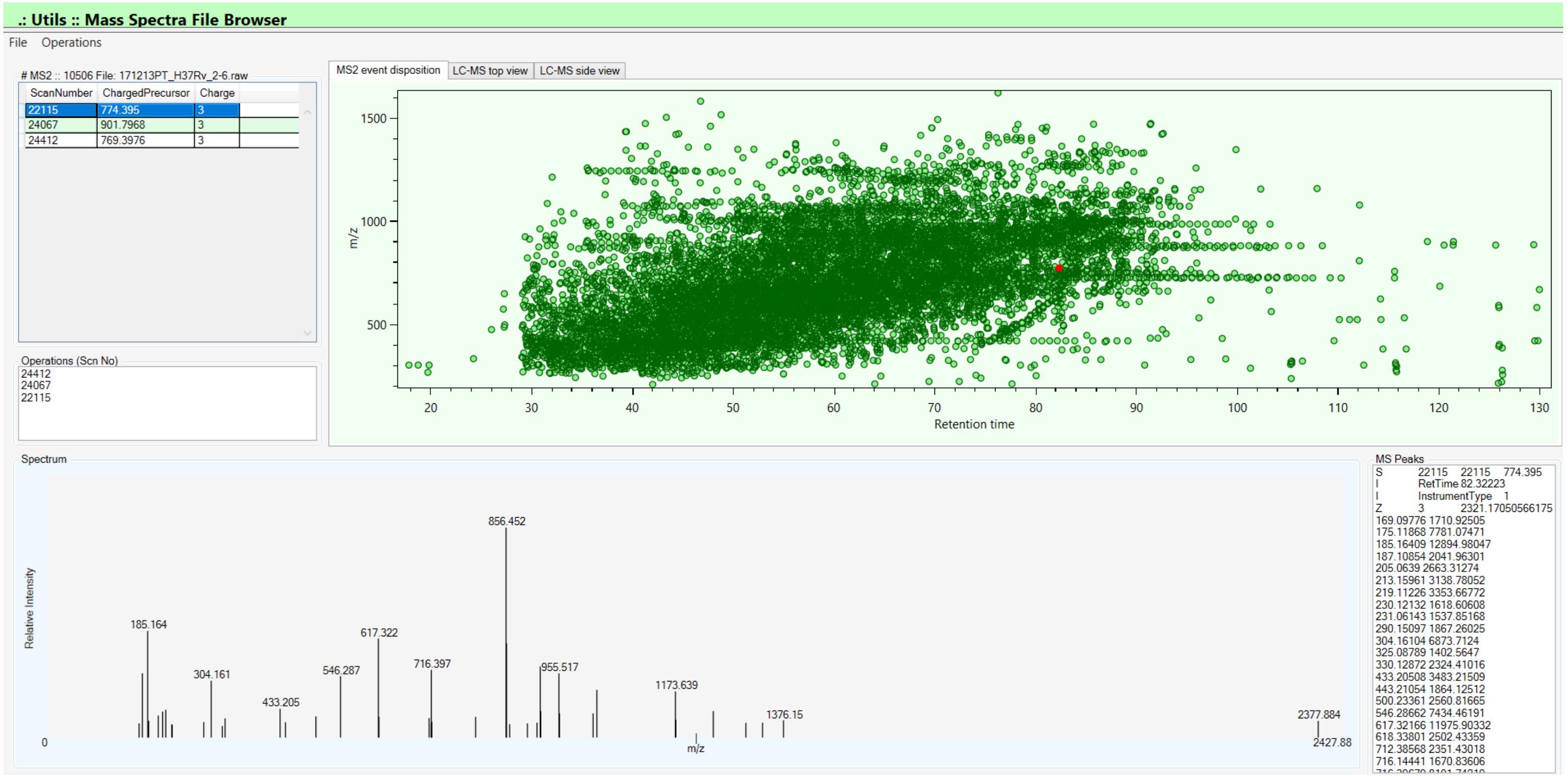

Peptide:
R.LVDDVGLDSVAFAVGM(+15.994900)VAIEER.L

MS/MS Fragmentation of **LVDDVGLDSVAFAVGMVAIEER**
Found in **Q73ZP6.1** in **NCBIprot**, RecName: Full=Acyl carrier protein MbtL; Short=ACP; AltName: Full=Mycobactin synthase protein L

Match to Query 2: 2320.163230 from(2321.170506,1+) index(0)
Data file Scans MbtL.txt

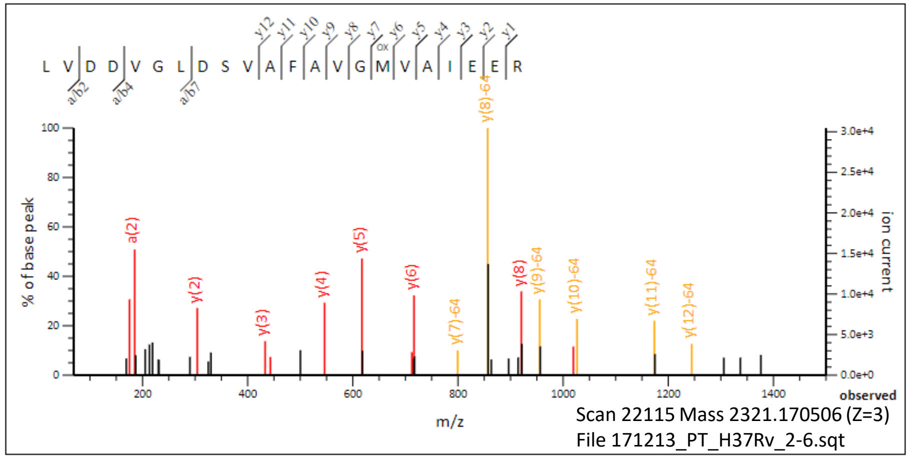


```
Monoisotopic mass of neutral peptide Mr(calc): 2320.1621
Fixed modifications: Carboxymethyl (C) (apply to specified residues or termini only)
Variable modifications:
M16    : Oxidation (M), with neutral losses 63.9983(shown in table), 0.0000
Ions Score: 77  Expect: 0.0019
Matches : 18/126 fragment ions using 29 most intense peaks   (help)
```

#	a	b	Seq.	y	y*	#
1	86.0964	114.0913	L			22
2	**185.1648**	213.1598	V	2144.0870	2127.0604	21
3	300.1918	328.1867	D	2045.0186	2027.9920	20
4	415.2187	**443.2136**	D	1929.9916	1912.9651	19
5	514.2871	542.2821	V	1814.9647	1797.9381	18
6	571.3086	599.3035	G	1715.8963	1698.8697	17
7	684.3927	**712.3876**	L	1658.8748	1641.8483	16
8	**799.4196**	827.4145	D	1545.7908	1528.7642	15
9	886.4516	914.4466	S	1430.7638	1413.7373	14
10	985.5201	1013.5150	V	1343.7318	1326.7052	13
11	1056.5572	1084.5521	A	***1244.6634***	1227.6368	12
12	1203.6256	1231.6205	F	***1173.6263***	1156.5997	11
13	1274.6627	1302.6576	A	***1026.5578***	1009.5313	10
14	1373.7311	1401.7260	V	***955.5207***	938.4942	9
15	1430.7526	1458.7475	G	***856.4523***	839.4258	8
16	1513.7897	1541.7846	M	***799.4308***	782.4043	7
17	1612.8581	1640.8530	V	***716.3937***	699.3672	6
18	1683.8952	1711.8901	A	***617.3253***	600.2988	5
19	1796.9793	1824.9742	I	***546.2882***	529.2617	4
20	1926.0219	1954.0168	E	***433.2041***	416.1776	3
21	2055.0645	2083.0594	E	***304.1615***	287.1350	2
22			R	***175.1190***	158.0924	1

MbtL (Rv1344) Acyl carrier protein (ACP) MbtL

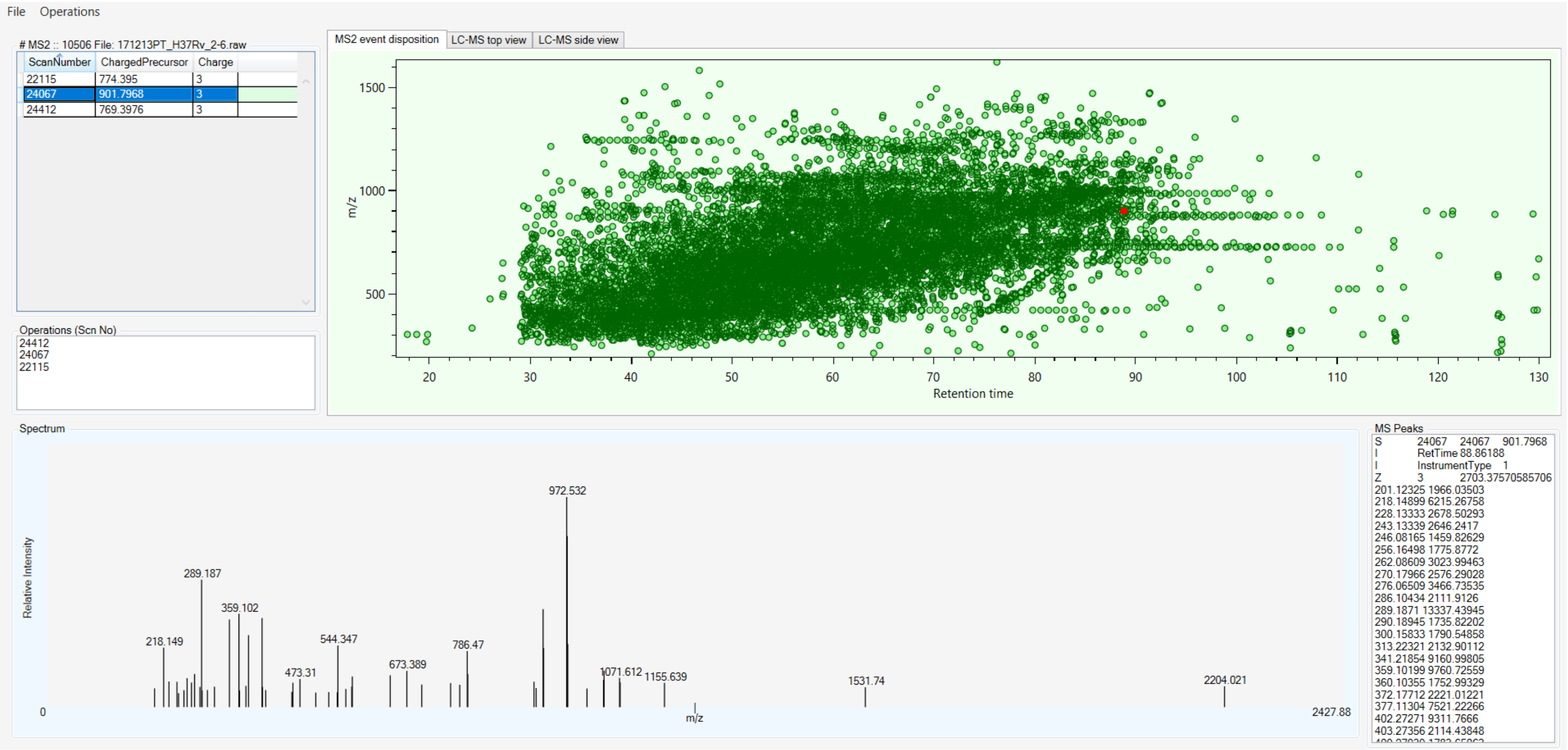

Peptide:
R.LGVALSEEELLTCDTVGELEAAIAAK.Y

MS/MS Fragmentation of **LGVALSEEELLTCDTVGELEAAIAAK**
Found in **KBE54844.1** in **NCBIprot**, acyl carrier protein MbtL [Mycobacterium tuberculosis H2102]

Match to Query 2: 2702.368430 from(2703.375706,1+) index(1)
Data file Scans MbtL.txt

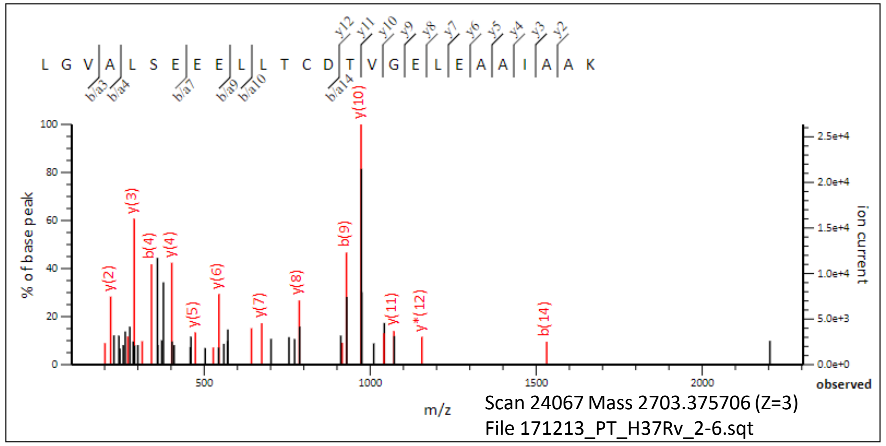

Supplement: S3 Fig — (PDF) [file pone.0221837.s004.pdf]
