# Supplementary material for: Integrative proteomic and glycoproteomic profiling of Mycobacterium tuberculosis culture filtrate
Source: PLoS One. 2020 Mar 3;15(3):e0221837. doi: 10.1371/journal.pone.0221837 (PMC7053730; doi:10.1371/journal.pone.0221837)
Supplement: S4 Fig — Some protein examples are shown: 1) Apa (modification: Hex), 2) LprF (modification: Hex), LppO (modification: Hex-Hex), Apa (modification: Hex-Hex-Hex). (PDF) [file pone.0221837.s005.pdf]

Apa (Rv1860) Mod=Hex

PatternLab for Proteomics Version=4.1.1.13

Generate Search DB | Search (Comet PSM) | Filter | Project Organization | Quant | Select | Analyze | Utils | Help

.: Filter :: Search Engine Processor (SEPro - for PSMs)

File | Statistics | Tools

Control | Follow up | Result Browser

Spec FDR: 44 / 36033 = 0.12% Pep FDR: 27 / 17879 = 0.15% Prot FDR: 14 / 1494 = 0.94% # Prot (Max Parsimony): 1474 Unique Prot: 1473 Unlabeled Decoys: 0 / 0

View mode: ○ Proteins ◉ Proteins Max Pars ○ Peptides ○ Scans alanine and pro

	enceCount	SpectrumCount	NSAF	Coverage	Protein Score	Description
1		84	0.00227426563649607	0.7262	292.4047	Alanine and proline-rich secreted protein Apa OS=Mycobacterium tuberculosis (strain ATCC 25618 / H37Rv) GN=apa PE=1 SV=1

	File Name	Scan No	Z	Unique	MeasuredMH	TheoreticalMH	PPM	PrimaryScore	SecondaryScore	DeltaCN	PeaksMatched	BayesianScore	PeptideSequence
69	171213_PT_H37Rv_1-3.sqt	20588	4	True	3133.529787	3132.549738	-7.43602	3.0849	5.15162298739723	0.36	38	0.5174	K.FSDPSKPNGQIWTGVIGSPAANAPDAGPPQR.W
70	171213_PT_H37Rv_1-3.sqt	19457	3	True	4243.27512	4240.27647	-2.6866	4.4726	9.46523262160497	0.4967	35	0.56732	K.ALAESIRPLVAPPPAPAPAPAEPAPAPAPAGEVAPTPTTPTPQR.T
71	171213_PT_H37Rv_1-3.sqt	19135	4	True	4242.275046	4240.27647	-1.91501	4.3682	7.61703184147197	0.4518	42	0.53736	K.ALAESIRPLVAPPPAPAPAPAEPAPAPAPAGEVAPTPTTPTPQR.T
72	171213_PT_H37Rv_1-3.sqt	18825	3	True	4566.377537	4564.382118	-2.47045	3.0734	4.99377817702983	0.0469	26	0.3594	K.ALAESIRPLVAPPPAPAPAPAEPAPAPAPAGEVAPTPT(+162.052824)T(+162.052824)PTPQR.T
73	171213_PT_H37Rv_1-3.sqt	18776	4	True	4566.377585	4564.382118	-2.45994	3.3228	5.22320989406123	0.0531	32	0.33023	K.ALAESIRPLVAPPPAPAPAPAEPAPAPAPAGEVAPTPT(+162.052824)T(+162.052824)PTPQR.T
74	171213_PT_H37Rv_1-3.sqt	18850	4	True	4564.378265	4564.382118	-0.84415	2.7023	-0.928219302739429	0.0578	32	0.31426	K.ALAESIRPLVAPPPAPAPAPAEPAPAPAPAGEVAPTPT(+162.052824)T(+162.052824)PTPQR.T
75	171213_PT_H37Rv_1-3.sqt	19094	3	True	4402.327971	4402.329294	-0.30052	3.9087	9.61081793857331	0.1726	28	0.38789	K.ALAESIRPLVAPPPAPAPAPAEPAPAPAPAGEVAPT(+162.052824)PTTPTPQR.T
76	171213_PT_H37Rv_1-3.sqt	19160	4	True	4405.340475	4402.329294	0.25673	4.0045	5.12941883008622	0.223	33	0.3666	K.ALAESIRPLVAPPPAPAPAPAEPAPAPAPAGEVAPT(+162.052824)PTTPTPQR.T
77	171213_PT_H37Rv_1-2.sqt	19370	4	True	4404.31655	4402.329294	-4.41476	2.9788	1.50058350752202	0.1886	33	0.34597	K.ALAESIRPLVAPPPAPAPAPAEPAPAPAPAGEVAPT(+162.052824)PTTPTPQR.T
78	171213_PT_H37Rv_1-3.sqt	20710	2	True	2807.54051	2807.545427	-1.75135	3.3637	12.0287636305598	0.5994	21	0.79808	K.ALAESIRPLVAPPPAPAPAPAEPAPAPAPA.G
79	171213_PT_H37Rv_1-3.sqt	20608	3	True	2807.539511	2807.545427	-2.10718	3.0844	6.91276782080568	0.38	19	0.65245	K.ALAESIRPLVAPPPAPAPAPAEPAPAPAPA.G
80	171213_PT_H37Rv_1-3.sqt	18551	3	True	2784.421482	2783.422352	-1.51558	3.1142	5.27655587476652	0.3501	28	0.71765	D.PSKPNGQIWTGVIGSPAANAPDAGPPQR.W
81	171213_PT_H37Rv_1-3.sqt	14912	2	True	1589.80266	1589.807067	-2.77204	3.2521	11.4641353008008	0.5696	16	0.80322	D.PPFPGQPPPVANDTR.I
82	171213_PT_H37Rv_1-3.sqt	15140	2	True	1832.016145	1832.017729	-0.86462	3.2069	12.1610992798874	0.6047	21	0.81038	D.PNAPPPPVIAPNAPQPVR.I
83	171213_PT_H37Rv_1-3.sqt	15989	3	True	2663.412205	2662.409996	-0.4284	3.4855	8.37309284744248	0.4948	26	0.76474	D.PNAAPPPADPNAPPPPVIAPNAPQPVR.I
84	171213_PT_H37Rv_1-3.sqt	31022	3	True	3212.554295	3210.553092	-1.7111	4.1085	14.6433326418508	0.5379	28	0.79416	D.NPVGGFSFALPAGWVESDAAHFDYGSALLSK.T

LprF (Rv1368) Mod=Hex

PatternLab for Proteomics Version=4.1.1.13

Generate Search DB | Search (Comet PSM) | Filter | Project Organization | Quant | Select | Analyze | Utils | Help

.: Filter :: Search Engine Processor (SEPro - for PSMs)

File | Statistics | Tools

Control | Follow up | Result Browser

Spec FDR: 44 / 36033 = 0.12% Pep FDR: 27 / 17879 = 0.15% Prot FDR: 14 / 1494 = 0.94% # Prot (Max Parsimony): 1474 Unique Prot: 1473 Unlabeled Decoys: 0 / 0

View mode: ○ Proteins ◉ Proteins Max Pars ○ Peptides ○ Scans lprf

	enceCount	SpectrumCount	NSAF	Coverage	Protein Score	Description
1		18	0.000606844279032202	0.6322	57.5418	Putative diacylated glycolipid transporter LprF OS=Mycobacterium tuberculosis (strain ATCC 25618 / H37Rv) GN=lprF PE=1 SV=1

	File Name	Scan No	Z	Unique	MeasuredMH	TheoreticalMH	PPM	PrimaryScore	SecondaryScore	DeltaCN	PeaksMatched	BayesianScore	PeptideSequence
3	171213_PT_H37Rv_1-4.sqt	22239	4	True	4216.116354	4213.12724	-4.96571	3.598	2.777400279237	0.2243	40	0.37338	K.GLHSVHVVVTVNNLSTLPFESVDADVTNQPQGNGQAVGNAK.V
4	171213_PT_H37Rv_1-4.sqt	17226	2	True	1417.766185	1417.768557	-1.67305	2.6259	7.75405363903626	0.5213	16	0.54237	K.IYDPGIILDKDR.G
5	171213_PT_H37Rv_1-4.sqt	17214	3	True	1417.766867	1417.768557	-1.19202	2.4377	5.11599580975408	0.3657	19	0.43355	K.IYDPGIILDKDR.G
6	171213_PT_H37Rv_1-4.sqt	21129	4	True	3093.67568	3092.685109	-4.13069	3.0991	4.24052707240018	0.3189	35	0.42083	K.IYDPGIILDKDRGLGAVVGQVQNPTIQGR.D
7	171213_PT_H37Rv_1-4.sqt	12961	3	True	2949.403721	2949.405879	-0.73167	3.4126	12.0909598384297	0.096	26	0.32854	K.KPTTASSPS(+162.052824)PGS(+162.052824)PSPEAQQILQDSSK.A
8	171213_PT_H37Rv_1-4.sqt	13266	3	True	2788.349278	2787.353055	-2.55599	3.0382	7.84680299797891	0.0764	29	0.31843	K.KPTTASSPSPGS(+162.052824)PSPEAQQILQDSSK.A
9	171213_PT_H37Rv_1-4.sqt	13707	3	True	2626.299839	2625.300231	-1.42482	3.8708	7.48045630646622	0.3047	31	0.42022	K.KPTTASSPSPGSPSPEAQQILQDSSK.A
10	171213_PT_H37Rv_1-4.sqt	8710	2	True	1334.666674	1334.669905	-2.42083	2.5707	6.67664355801875	0.4374	14	0.48627	K.RGGDYVSVGPAEK.I
11	171213_PT_H37Rv_1-4.sqt	23603	3	True	1893.068761	1893.069156	-0.20866	2.7493	6.46306945772069	0.4334	20	0.4939	K.VSGTIDAAVIDPIVPQLGK.G
12	171213_PT_H37Rv_1-4.sqt	23535	2	True	1893.064295	1893.069156	-2.56779	3.2671	9.83957422679248	0.525	17	0.56508	K.VSGTIDAAVIDPIVPQLGK.G
13	171213_PT_H37Rv_1-4.sqt	14269	2	True	1671.818256	1671.81842	-0.0981	3.0646	9.62585581593785	0.5693	17	0.79537	P.GSPSPEAQQILQDSSK.A
14	171213_PT_H37Rv_1-4.sqt	13027	2	True	1002.544191	1002.546602	-2.40488	1.6905	0.109814866007207	0.2561	12	0.35807	R.DAIDGLATVK.V
15	171213_PT_H37Rv_1-4.sqt	10611	2	True	1178.566698	1178.568794	-1.77843	1.4583	-0.157003748809665	0.1533	9	0.29922	R.GGDYVSVGPAEK.I
16	171213_PT_H37Rv_1-4.sqt	16227	2	True	1693.926806	1693.934393	-4.47894	3.4524	11.7537239515232	0.5277	17	0.5593	R.GLGAVVGQVQNPTIQGR.D
17	171213_PT_H37Rv_1-4.sqt	16305	3	True	1693.936341	1693.934393	1.14998	3.2621	9.66132599538616	0.5395	21	0.54928	R.GLGAVVGQVQNPTIQGR.D
18	171213_PT_H37Rv_1-4.sqt	28521	3	True	2433.346958	2433.350022	-1.25917	3.7486	8.2023824515762	0.4944	27	0.56355	R.LPITLWIVDTNASTPAPAANLVR.M

LppO (Rv2290) Mod= Hex-Hex

PatternLab for Proteomics Version=4.1.1.13

Generate Search DB | Search (Comet PSM) | Filter | Project Organization | Quant | Select | Analyze | Utils | Help

.: Filter :: Search Engine Processor (SEPro - for PSMs)

File | Statistics | Tools

Control | Follow up | Result Browser

Spec FDR: 47 / 35750 = 0.13% Pep FDR: 22 / 17513 = 0.13% Prot FDR: 14 / 1467 = 0.95% # Prot (Max Parsimony): 1454 Unique Prot: 1453 Unlabeled Decoys: 0 / 0

View mode: ○ Proteins ◉ Proteins Max Pars ○ Peptides ○ Scans lppo

		Coverage	Protein Score	Description
1	:6801147621	0.386	12.924	Putative lipoprotein LppO OS=Mycobacterium tuberculosis (strain ATCC 25618 / H37Rv) GN=lppO PE=1 SV=1

	File Name	Scan No	Z	Unique	MeasuredMH	TheoreticalMH	PPM	PrimaryScore	SecondaryScore	DeltaCN	PeaksMatched	BayesianScore	PeptideSequence
1	171213_PT_H37Rv_1-5.sqt	13877	3	True	2610.249681	2610.250429	-0.28656	3.1995	10.404362845449	0.504	23	0.62307	K.IGSVDYQM(+15.994900)PYQPVQSPTQVEATR.Q
2	171213_PT_H37Rv_1-5.sqt	16352	3	True	2594.248692	2594.255529	-2.63545	2.0502	2.81842325858358	0.2536	17	0.40816	K.IGSVDYQMPYQPVQSPTQVEATR.Q
3	171213_PT_H37Rv_1-5.sqt	3439	2	True	1660.735033	1660.739514	-2.6982	1.8711	6.2606520369236	0.3618	10	0.45859	R.TAT(+324.105600)PSESGTQTTR.V
4	171213_PT_H37Rv_1-5.sqt	3796	2	True	1336.631226	1336.633914	-2.01103	2.1087	7.02428909523809	0.5115	12	0.57607	R.TATPSESGTQTTR.V
5	171213_PT_H37Rv_1-5.sqt	20182	3	True	3044.490025	3043.485466	0.39711	3.6945	8.62255370707406	0.4541	29	0.58379	R.VNAHDDSASVTLSLSDSTPPDVNGFGISLK.I

Apa (Rv1860) Mod=Hex-Hex-Hex

PatternLab for Proteomics Version=4.1.1.13

Generate Search DB | Search (Comet PSM) | Filter | Project Organization | Quant | Select | Analyze | Utils | Help

.: Filter :: Search Engine Processor (SEPro - for PSMs)

File | Statistics | Tools

Control | Follow up | Result Browser

Spec FDR: 49 / 36000 = 0.14% Pep FDR: 24 / 17635 = 0.14% Prot FDR: 15 / 1505 = 1% # Prot (Max Parsimony): 1487 Unique Prot: 1482 Unlabeled Decoys: 0 / 0

View mode: ( ) Proteins (•) Proteins Max Pars ( ) Peptides ( ) Scans   alan

	Coverage	Protein Score	Description
1	0.7262	290.7926	Alanine and proline-rich secreted protein Apa OS=Mycobacterium tuberculosis (strain ATCC 25618 / H37Rv) GN=apa PE=1 SV=1

	File Name	Scan No	Z	Unique	MeasuredMH	TheoreticalMH	PPM	PrimaryScore	SecondaryScore	DeltaCN	PeaksMatched	BayesianScore	PeptideSequence
4	171213_PT_H37Rv_1-3.sqt	14912	2	True	1589.80266	1589.807067	-2.77204	3.2521	9.63346041532307	0.5696	16	0.87783	D.PPFPGQPPPVANDTR.I
5	171213_PT_H37Rv_1-3.sqt	18551	3	True	2784.421482	2783.422352	-1.51558	3.1142	7.05741605353654	0.4147	28	0.88319	D.PSKPNGQIWTGVIGSPAANAPDAGPPQR.W
6	171213_PT_H37Rv_1-3.sqt	20710	2	True	2807.54051	2807.545427	-1.75135	3.3637	13.9582268601659	0.6612	21	0.88537	K.ALAESIRPLVAPPPAPAPAPAEPAPAPAPA.G
7	171213_PT_H37Rv_1-3.sqt	20608	3	True	2807.539511	2807.545427	-2.10718	3.0844	6.13302811142977	0.4044	19	0.74689	K.ALAESIRPLVAPPPAPAPAPAEPAPAPAPA.G
8	171213_PT_H37Rv_1-3.sqt	18664	4	True	4727.432272	4726.43487	-1.25819	3.8271	6.21460809842219	0.2129	40	0.45812	K.ALAESIRPLVAPPPAPAPAPAEPAPAPAPAGEVAPT(+486.158400)PTTPTPQR.T
9	171213_PT_H37Rv_1-2.sqt	18976	4	True	4729.443991	4726.43487	-0.19643	2.8751	0.632993257740198	0.1703	34	0.44238	K.ALAESIRPLVAPPPAPAPAPAEPAPAPAPAGEVAPT(+486.158400)PTTPTPQR.T
10	171213_PT_H37Rv_1-3.sqt	18680	3	True	4729.444676	4726.43487	-0.05159	2.9347	0.809680996815897	0.1332	24	0.43961	K.ALAESIRPLVAPPPAPAPAPAEPAPAPAPAGEVAPT(+486.158400)PTTPTPQR.T
11	171213_PT_H37Rv_1-3.sqt	18662	4	True	4729.442038	4726.43487	-0.60937	3.2876	3.21637894466961	0.2441	39	0.46967	K.ALAESIRPLVAPPPAPAPAPAEPAPAPAPAGEVAPT(+486.158400)PTTPTPQR.T
12	171213_PT_H37Rv_1-2.sqt	18970	3	True	4729.434055	4726.43487	-2.29732	2.8923	4.45675018086982	0.1416	27	0.43944	K.ALAESIRPLVAPPPAPAPAPAEPAPAPAPAGEVAPT(+486.158400)PTTPTPQR.T
13	171213_PT_H37Rv_1-4.sqt	17848	4	True	4727.4186	4726.43487	-4.15026	2.5616	0.877070018720874	0.116	38	0.41738	K.ALAESIRPLVAPPPAPAPAPAEPAPAPAPAGEVAPT(+486.158400)PTTPTPQR.T
14	171213_PT_H37Rv_1-3.sqt	19135	4	True	4242.275046	4240.27647	-1.91501	4.3682	8.58976388425107	0.4678	42	0.53825	K.ALAESIRPLVAPPPAPAPAPAEPAPAPAPAGEVAPTPTTPTPQR.T
15	171213_PT_H37Rv_1-3.sqt	19457	3	True	4243.27512	4240.27647	-2.6866	4.4726	13.9399406363425	0.4594	35	0.52648	K.ALAESIRPLVAPPPAPAPAPAEPAPAPAPAGEVAPTPTTPTPQR.T
16	171213_PT_H37Rv_1-2.sqt	20426	3	True	3133.547581	3132.549738	-1.75743	4.8469	17.0700135604756	0.6323	34	0.75078	K.FSDPSKPNGQIWTGVIGSPAANAPDAGPPQR.W
17	171213_PT_H37Rv_1-4.sqt	19127	4	True	3133.548727	3132.549738	-1.39171	3.7053	7.5752347127935	0.4352	30	0.59224	K.FSDPSKPNGQIWTGVIGSPAANAPDAGPPQR.W
18	171213_PT_H37Rv_1-3.sqt	20066	2	True	3133.550585	3132.549738	-0.79877	4.7078	22.519033327572	0.6916	27	0.77766	K.FSDPSKPNGQIWTGVIGSPAANAPDAGPPQR.W
19	171213_PT_H37Rv_1-3.sqt	20441	3	True	3133.533824	3132.549738	-6.14769	3.9926	11.9498812401698	0.5666	28	0.6711	K.FSDPSKPNGQIWTGVIGSPAANAPDAGPPQR.W
[illegible]	[illegible]	[illegible]	[illegible]	[illegible]	[illegible]	[illegible]	[illegible]	[illegible]	[illegible]	[illegible]	[illegible]	[illegible]	[illegible]
