# Supplementary material for: Integrative proteomic and glycoproteomic profiling of Mycobacterium tuberculosis culture filtrate
Source: PLoS One. 2020 Mar 3;15(3):e0221837. doi: 10.1371/journal.pone.0221837 (PMC7053730; doi:10.1371/journal.pone.0221837)

Probable conserved lipoprotein DsbF (Rv1677)
Modification: Hex-Hex

	File Name	Scan No	Z	Unique	MeasuredMH	TheoreticalMH	PPM	PrimaryScore	SecondaryScore	DeltaCN	PeaksMatched	BayesianScore	PeptideSequence
1	171213_PT_H37Rv_1-5.sqt	23510	4	True	4023.931051	4022.930772	-0.76318	3.7776	12.5207833903699	0.5146	48	0.63195	K.TFTQLADTDGSVWANFGVTQQPAYAFVDPHGNVDVVR.G
2	171213_PT_H37Rv_1-5.sqt	23537	3	True	4025.932224	4022.930772	-2.13565	3.23	11.2660653870387	0.5172	34	0.61561	K.TFTQLADTDGSVWANFGVTQQPAYAFVDPHGNVDVVR.G
3	171213_PT_H37Rv_1-5.sqt	14987	3	True	3403.688206	3402.691054	-1.82097	3.433	9.45752050111863	0.4195	28	0.53295	K.SQPAVAPTGDAAAAT(+324.105600)QVPAGQTVPAQLQFSAK.T
4	171213_PT_H37Rv_1-5.sqt	5339	2	True	995.445299	995.446222	-0.92722	1.3154	0.179126665897435	0.274	7	0.37592	R.M(+15.994900)SQDELTR.R
5	171213_PT_H37Rv_1-5.sqt	6210	3	True	1135.551226	1135.552433	-1.06292	1.6862	0.603306476560156	0.1518	12	0.33335	R.MSQDELTRR.V

☑ All peptide labels   Infer Domains

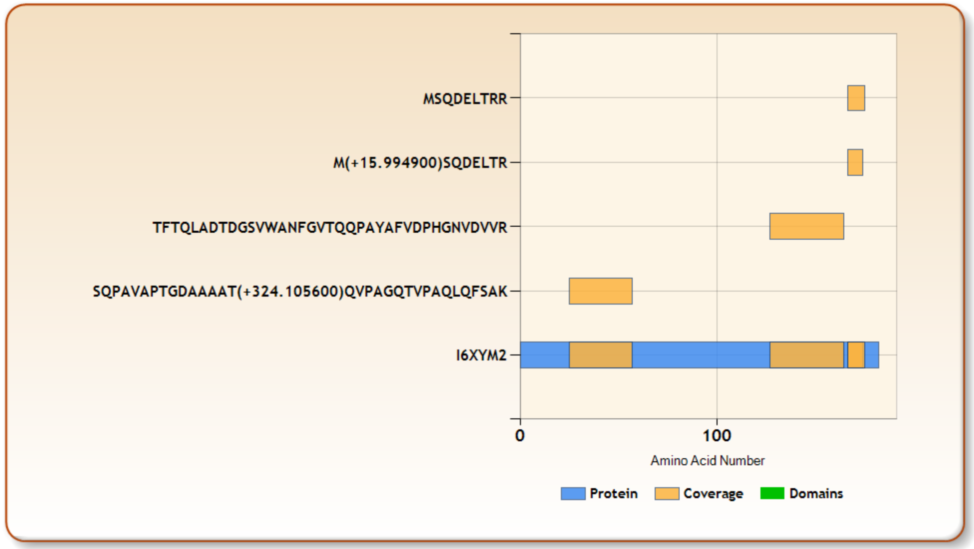

Peptide View

MS/MS Fragmentation of **SQPAVAPTGDAAAATQVPAGQTVPAQLQFSAK**
Found in **AFE16571.1** in **NCBIprot**, putative lipoprotein DSBF [Mycobacterium tuberculosis RGTB327]

Match to Query 4: 3401.680930 from(3402.688206,1+) index(1)
Data file Scans DsbF Hex-Hex Mascot.txt

171213_PT_H37Rv_1-5.sqt
Scan=14987 z=3

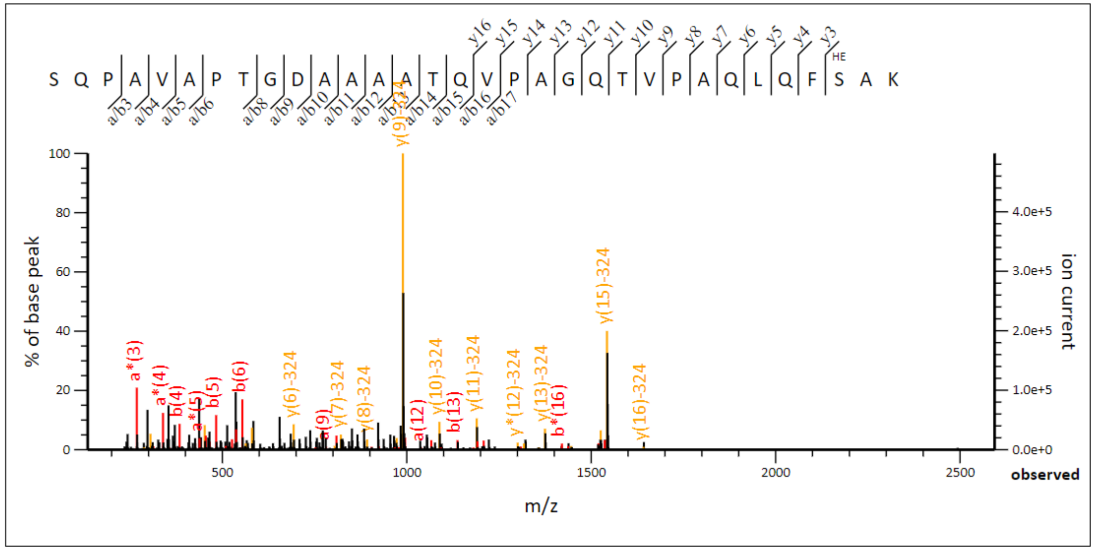

Protein family 1 (out of 1)

10 per page 1 [Expand all] [Collapse all]

Accession contains [Find] [Clear]

▼1 **AFE16571.1** 75 putative lipoprotein DSBF [Mycobacterium tuberculosis RGTB327]

		Score	Mass	Matches	Sequences	
1.1	AFE16571.1	75	19224	2 (2)	1 (1)	putative lipoprotein DSBF [Mycobacterium tuberculosis RGTB327]

▶23 samesets of AFE16571.1

▼*2 peptide matches (1 non-duplicate, 1 duplicate)*

☑ Auto-fit to window

Query	Dupes	Observed	Mr(expt)	Mr(calc)	ppm	M	Score	Expect	Rank	U	Peptide
4	▼1	3402.6882	3401.6809	3401.6838	-0.85	0	48	9.2e-05	▶1	U	K.SQPAVAPTGDAAAATQVPAGQTVPAQLQFSAK.T + Hex(2) (ST)
3		3402.6834	3401.6762	3401.6838	-2.25	0	(47)	0.00012	▶1	U	K.SQPAVAPTGDAAAATQVPAGQTVPAQLQFSAK.T + Hex(2) (ST)

Liporotein O (LppO, Rv2290)
Modification: Hex

Mascot Score Histogram

Ions score is -10*Log(P), where P is the probability that the observed match is a random event.
Individual ions scores > 38 indicate identity or extensive homology (p<0.05).
Protein scores are derived from ions scores as a non-probabilistic basis for ranking protein hits.

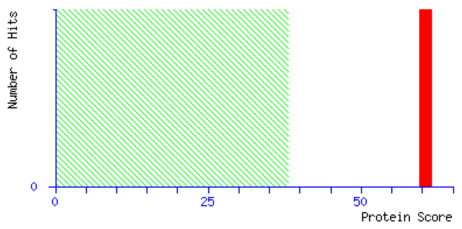

Peptide Summary Report

Format As: Peptide Summary — Help
Significance threshold p< 0.05 — Max. number of hits AUTO
Standard scoring — MudPIT scoring — Display non-significant matches — Show sub-sets 0
Show pop-ups — Suppress pop-ups — Sort unassigned Decreasing Score — Require bold red
Preferred taxonomy All entries

Select All — Select None — Search Selected — Error tolerant

1. CKZ21714.1 Mass: 12789 Score: 61 Matches: 3(3) Sequences: 1(1)
lipoprotein [Mycobacterium tuberculosis]
Check to include this hit in error tolerant search

Query	Observed	Mr(expt)	Mr(calc)	ppm	Miss	Score	Expect	Rank	Unique	Peptide
1	1336.6320	1335.6247	1335.6266	-1.44	0	(45)	0.017	1	U	R.TATPSESGTQTTR.V
2	1498.6841	1497.6769	1497.6795	-1.75	0	61	0.00034	1	U	R.TATPSESGTQTTR.V + Hex (ST)
3	1660.7371	1659.7298	1659.7323	-1.49	0	(48)	0.0048	1	U	R.TATPSESGTQTTR.V + 2 Hex (ST)

Sequence Explorer
Fasta Coverage — Protein Coverage — Group view
All peptide labe — Infer Domains

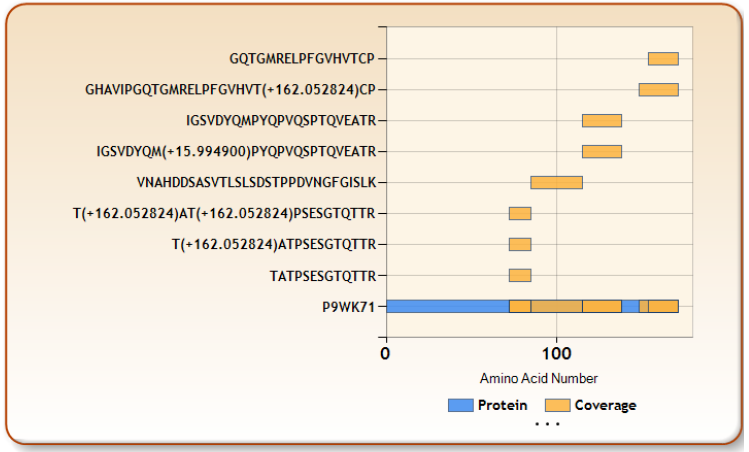

Match to Query 1: 1335.624730 from(1336.632006,1+) index(2)
Data file P9WK71 R1-5.txt

171213_PT_H37Rv_1-5.sqt
Scan=3796 z=2

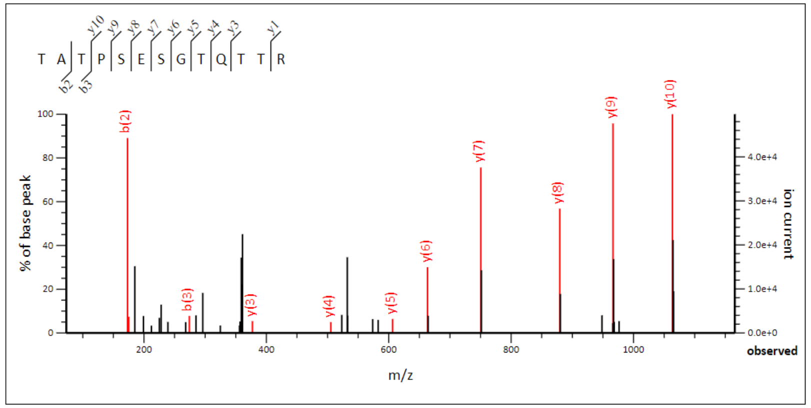

Match to Query 2: 1497.676854 from(1498.684130,1+) index(1)
Data file P9WK71 R1-5.txt

171213_PT_H37Rv_1-5.sqt
Scan=3473 z=2

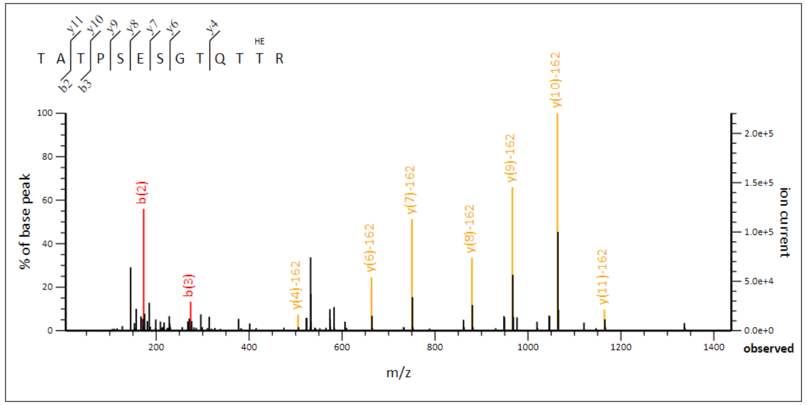

Match to Query 3: 1659.729832 from(1660.737108,1+) index(0)
Data file P9WK71 R1-5.txt

171213_PT_H37Rv_1-5.sqt
Scan=3439 z=2

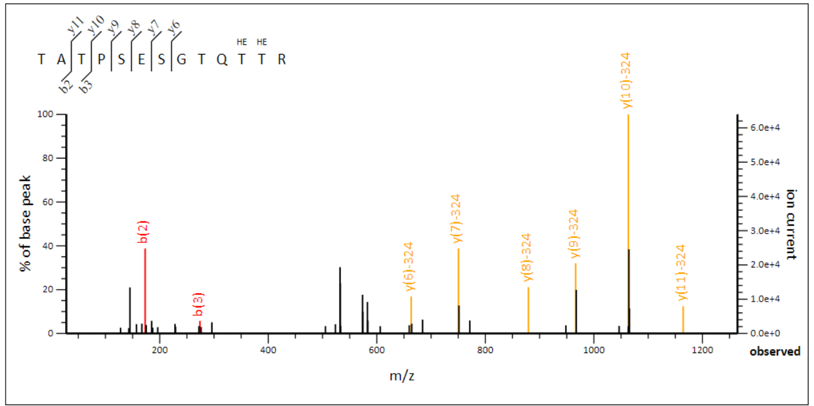

Periplasmic phosphate-binding lipoprotein PstS1 (Rv0934)
Modification: Hex

MS/MS Fragmentation of **PANQAISMIDGPAPDGYPIINYEYAIVNNR**
Found in **WP_014585113.1** in **NCBIprot**, phosphate ABC transporter substrate-binding protein PstS [Mycobacterium tuberculosis]

Match to Query 3: 3453.638816 from(3454.646092,1+) index(2)
Data file Scans pstS1 Hex Mascot.txt

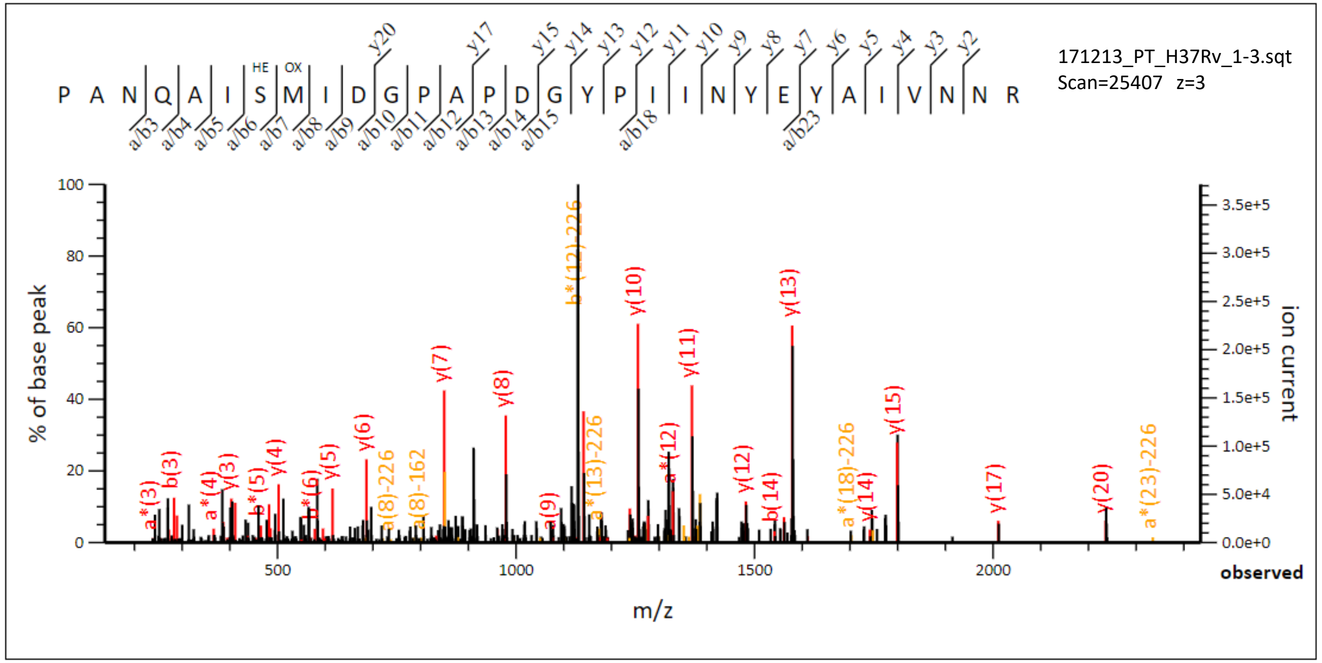

#	a	a*	b	b*	Seq.	y	y*	#
1	70.0651		98.0600		P			30
2	141.1022		169.0972		A	3357.5944	3340.5678	29
3	255.1452	238.1186	283.1401	266.1135	N	3286.5573	3269.5307	28
4	383.2037	366.1772	411.1987	394.1721	Q	3172.5143	3155.4878	27
5	454.2409	437.2143	482.2358	465.2092	A	3044.4557	3027.4292	26
6	567.3249	550.2984	595.3198	578.2933	I	2973.4186	2956.3921	25
7	816.4098	799.3832	844.4047	827.3781	S	2860.3346	2843.3080	24
8	963.4452	946.4186	991.4401	974.4135	M	2611.2497	2594.2232	23
9	1076.5292	1059.5027	1104.5242	1087.4976	I	2464.2143	2447.1878	22
10	1191.5562	1174.5296	1219.5511	1202.5245	D	2351.1303	2334.1037	21
11	1248.5776	1231.5511	1276.5726	1259.5460	G	2236.1033	2219.0768	20
12	1345.6304	1328.6039	1373.6253	1356.5988	P	2179.0818	2162.0553	19
13	1416.6675	1399.6410	1444.6624	1427.6359	A	2082.0291	2065.0025	18
14	1513.7203	1496.6937	1541.7152	1524.6887	P	2010.9920	1993.9654	17
15	1628.7472	1611.7207	1656.7421	1639.7156	D	1913.9392	1896.9127	16
16	1685.7687	1668.7421	1713.7636	1696.7371	G	1798.9123	1781.8857	15
17	1848.8320	1831.8055	1876.8269	1859.8004	Y	1741.8908	1724.8642	14
18	1945.8848	1928.8582	1973.8797	1956.8532	P	1578.8275	1561.8009	13
19	2058.9689	2041.9423	2086.9638	2069.9372	I	1481.7747	1464.7482	12
20	2172.0529	2155.0264	2200.0478	2183.0213	I	1368.6906	1351.6641	11
21	2286.0958	2269.0693	2314.0908	2297.0642	N	1255.6066	1238.5800	10
22	2449.1592	2432.1326	2477.1541	2460.1275	Y	1141.5636	1124.5371	9
23	2578.2018	2561.1752	2606.1967	2589.1701	E	978.5003	961.4738	8
24	2741.2651	2724.2385	2769.2600	2752.2335	Y	849.4577	832.4312	7
25	2812.3022	2795.2757	2840.2971	2823.2706	A	686.3944	669.3678	6
26	2925.3863	2908.3597	2953.3812	2936.3546	I	615.3573	598.3307	5
27	3024.4547	3007.4281	3052.4496	3035.4231	V	502.2732	485.2467	4
28	3138.4976	3121.4711	3166.4925	3149.4660	N	403.2048	386.1783	3
29	3252.5405	3235.5140	3280.5355	3263.5089	N	289.1619	272.1353	2
30					R	175.1190	158.0924	1

Conserved protein with FHA domain, FhaA (Rv0020c)
Modification: Hex

MS/MS Fragmentation of **EGSYVPSGPPGPPEQRPAYPDQGGYDQGYQQGATTYGR**
Found in **WP_003912368.1** in **NCBIprot**, phosphopeptide-binding protein [Mycobacterium tuberculosis]

Match to Query 3: 4350.924996 from(4351.932272,1+) index(2)
Data file Scans FhaA Hex-Hex-Hex Mascot.txt

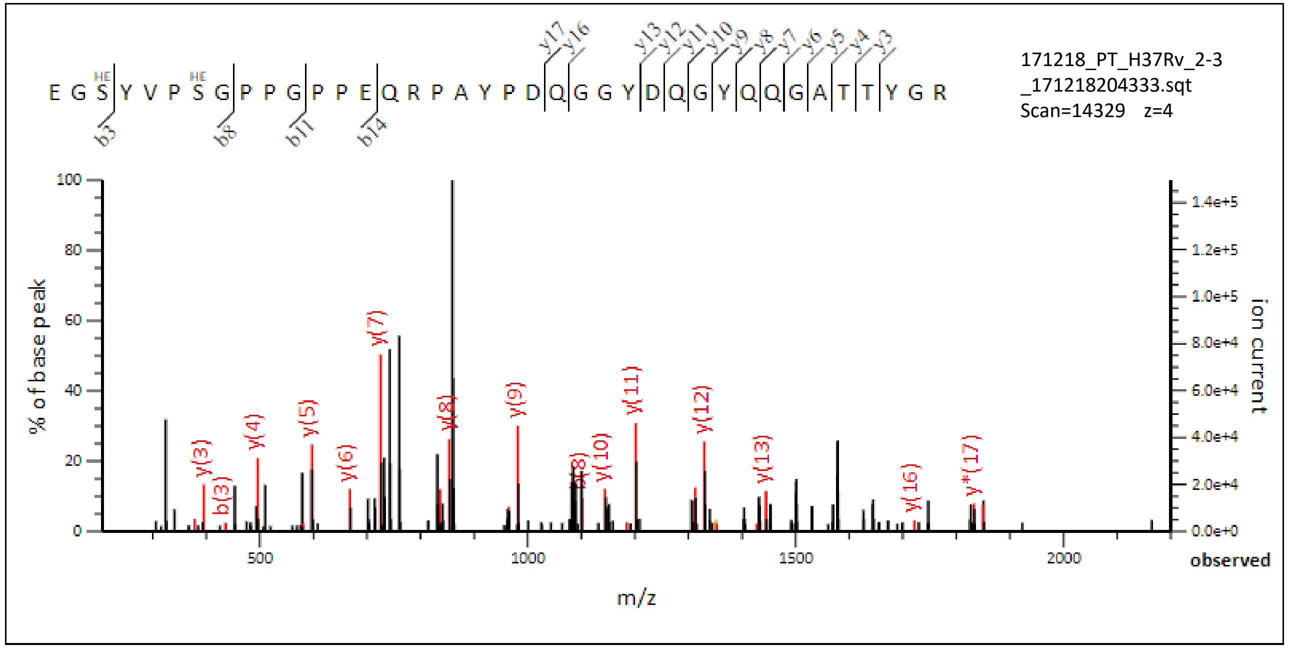

#	a	a*	b	b*	Seq.	y	y*	#
1	102.0550		130.0499		E			38
2	159.0764		187.0713		G	4222.8748	4205.8483	37
3	408.1613		436.1562		S	4165.8534	4148.8268	36
4	571.2246		599.2195		Y	3916.7685	3899.7420	35
5	670.2930		698.2879		V	3753.7052	3736.6786	34
6	767.3458		795.3407		P	3654.6368	3637.6102	33
7	1016.4306		1044.4255		S	3557.5840	3540.5575	32
8	1073.4521		1101.4470		G	3308.4992	3291.4726	31
9	1170.5049		1198.4998		P	3251.4777	3234.4512	30
10	1267.5576		1295.5525		P	3154.4249	3137.3984	29
11	1324.5791		1352.5740		G	3057.3722	3040.3456	28
12	1421.6318		1449.6268		P	3000.3507	2983.3242	27
13	1518.6846		1546.6795		P	2903.2979	2886.2714	26
14	1647.7272		1675.7221		E	2806.2452	2789.2186	25
15	1775.7858	1758.7592	1803.7807	1786.7541	Q	2677.2026	2660.1760	24
16	1931.8869	1914.8603	1959.8818	1942.8553	R	2549.1440	2532.1175	23
17	2028.9397	2011.9131	2056.9346	2039.9080	P	2393.0429	2376.0164	22
18	2099.9768	2082.9502	2127.9717	2110.9451	A	2295.9901	2278.9636	21
19	2263.0401	2246.0136	2291.0350	2274.0085	Y	2224.9530	2207.9265	20
20	2360.0929	2343.0663	2388.0878	2371.0612	P	2061.8897	2044.8631	19
21	2475.1198	2458.0933	2503.1147	2486.0882	D	1964.8369	1947.8104	18
22	2603.1784	2586.1518	2631.1733	2614.1468	Q	1849.8100	1832.7834	17
23	2660.1999	2643.1733	2688.1948	2671.1682	G	1721.7514	1704.7249	16
24	2717.2213	2700.1948	2745.2162	2728.1897	G	1664.7299	1647.7034	15
25	2880.2846	2863.2581	2908.2796	2891.2530	Y	1607.7085	1590.6819	14
26	2995.3116	2978.2850	3023.3065	3006.2800	D	1444.6452	1427.6186	13
27	3123.3702	3106.3436	3151.3651	3134.3385	Q	1329.6182	1312.5917	12
28	3180.3916	3163.3651	3208.3865	3191.3600	G	1201.5596	1184.5331	11
29	3343.4550	3326.4284	3371.4499	3354.4233	Y	1144.5382	1127.5116	10
30	3471.5135	3454.4870	3499.5085	3482.4819	Q	981.4748	964.4483	9
31	3599.5721	3582.5456	3627.5670	3610.5405	Q	853.4163	836.3897	8
32	3656.5936	3639.5670	3684.5885	3667.5619	G	725.3577	708.3311	7
33	3727.6307	3710.6041	3755.6256	3738.5991	A	668.3362	651.3097	6
34	3828.6784	3811.6518	3856.6733	3839.6467	T	597.2991	580.2726	5
35	3929.7261	3912.6995	3957.7210	3940.6944	T	496.2514	479.2249	4
36	4092.7894	4075.7628	4120.7843	4103.7577	Y	395.2037	378.1772	3
37	4149.8108	4132.7843	4177.8058	4160.7792	G	232.1404	215.1139	2
38					R	175.1190	158.0924	1

Two component transcriptional regulatory protein DevR (Rv3133c)
Modification: Hex-Hex

MS/MS Fragmentation of **LSGLTDQERTLLGLLSEGLTNKQIADR**
Found in **AFE17937.1** in **NCBIprot**, two component transcriptional regulatory protein [Mycobacterium tuberculosis RGTB327]

Match to Query 3: 3264.691184 from(3265.698460,1+) index(1)
Data file Scans DevR Hex-Hex Mascot.txt

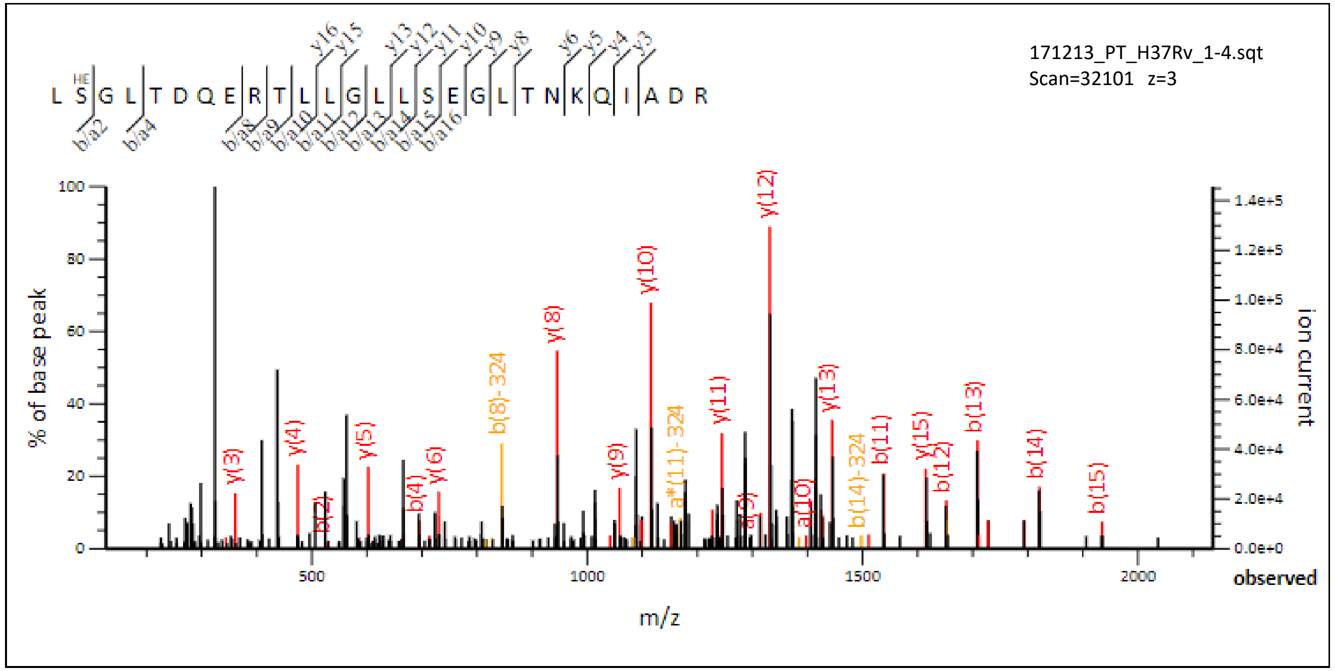

#	a	a*	b	b*	Seq.	y	y*	#
1	86.0964		114.0913		L			27
2	497.2341		525.2290		S	3152.6169	3135.5903	26
3	554.2556		582.2505		G	2741.4792	2724.4527	25
4	667.3396		695.3345		L	2684.4577	2667.4312	24
5	768.3873		796.3822		T	2571.3737	2554.3471	23
6	883.4143		911.4092		D	2470.3260	2453.2994	22
7	1011.4728	994.4463	1039.4677	1022.4412	Q	2355.2990	2338.2725	21
8	1140.5154	1123.4889	1168.5103	1151.4838	E	2227.2405	2210.2139	20
9	1296.6165	1279.5900	1324.6114	1307.5849	R	2098.1979	2081.1713	19
10	1397.6642	1380.6377	1425.6591	1408.6326	T	1942.0968	1925.0702	18
11	1510.7483	1493.7217	1538.7432	1521.7166	L	1841.0491	1824.0225	17
12	1623.8323	1606.8058	1651.8273	1634.8007	L	1727.9650	1710.9385	16
13	1680.8538	1663.8273	1708.8487	1691.8222	G	1614.8810	1597.8544	15
14	1793.9379	1776.9113	1821.9328	1804.9062	L	1557.8595	1540.8329	14
15	1907.0219	1889.9954	1935.0168	1917.9903	L	1444.7754	1427.7489	13
16	1994.0540	1977.0274	2022.0489	2005.0223	S	1331.6914	1314.6648	12
17	2123.0966	2106.0700	2151.0915	2134.0649	E	1244.6593	1227.6328	11
18	2180.1180	2163.0915	2208.1129	2191.0864	G	1115.6167	1098.5902	10
19	2293.2021	2276.1755	2321.1970	2304.1704	L	1058.5953	1041.5687	9
20	2394.2498	2377.2232	2422.2447	2405.2181	T	945.5112	928.4847	8
21	2508.2927	2491.2661	2536.2876	2519.2611	N	844.4635	827.4370	7
22	2636.3876	2619.3611	2664.3826	2647.3560	K	730.4206	713.3941	6
23	2764.4462	2747.4197	2792.4411	2775.4146	Q	602.3257	585.2991	5
24	2877.5303	2860.5037	2905.5252	2888.4987	I	474.2671	457.2405	4
25	2948.5674	2931.5409	2976.5623	2959.5358	A	361.1830	344.1565	3
26	3063.5943	3046.5678	3091.5893	3074.5627	D	290.1459	273.1193	2
27					R	175.1190	158.0924	1

Probable chaperone protein DnaK (heat shock 70 kDa protein) (Rv0350)
Modification: Hex

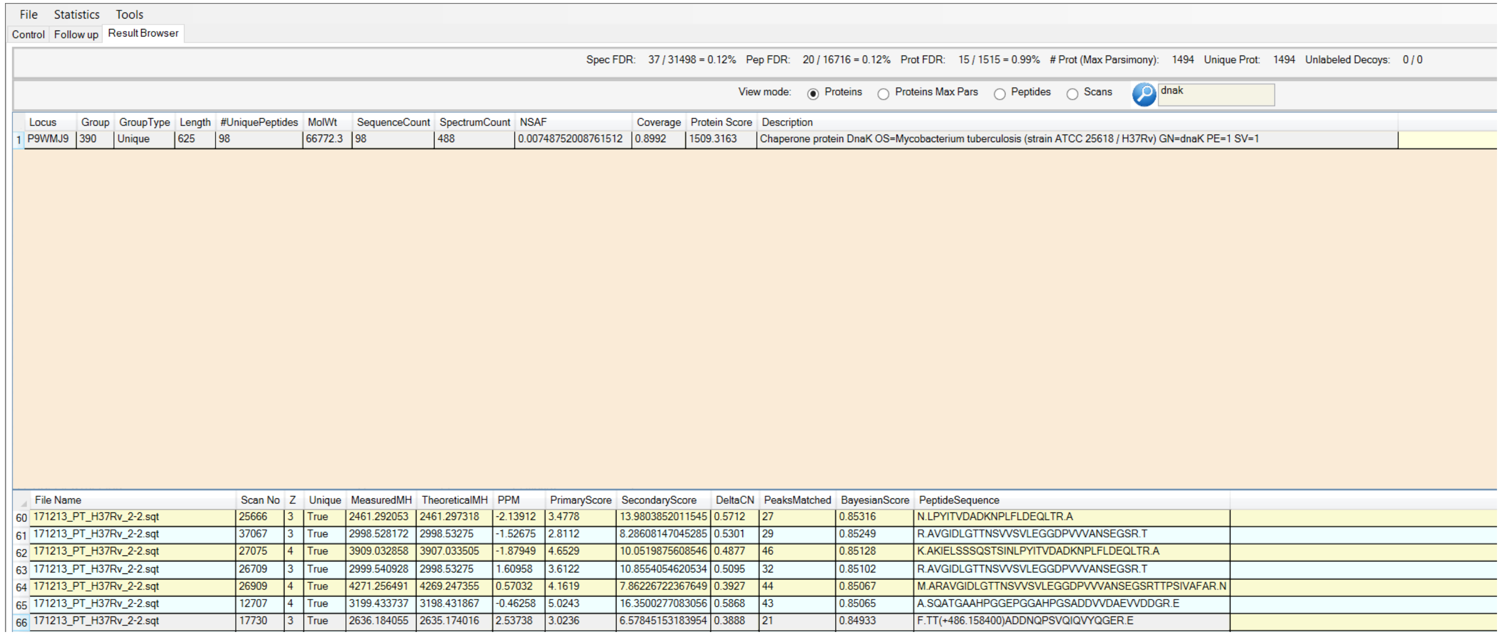

File Statistics Tools

Control | Follow up | Result Browser

Spec FDR: 37 / 31498 = 0.12% Pep FDR: 20 / 16716 = 0.12% Prot FDR: 15 / 1515 = 0.99% # Prot (Max Parsimony): 1494 Unique Prot: 1494 Unlabeled Decoys: 0 / 0

View mode: Proteins | Proteins Max Pars | Peptides | Scans | dnak

	Locus	Group	GroupType	Length	#UniquePeptides	MolWt	SequenceCount	SpectrumCount	NSAF	Coverage	Protein Score	Description
1	P9WMJ9	390	Unique	625	98	66772.3	98	488	0.00748752008761512	0.8992	1509.3163	Chaperone protein DnaK OS=Mycobacterium tuberculosis (strain ATCC 25618 / H37Rv) GN=dnaK PE=1 SV=1

	File Name	Scan No	Z	Unique	MeasuredMH	TheoreticalMH	PPM	PrimaryScore	SecondaryScore	DeltaCN	PeaksMatched	BayesianScore	PeptideSequence
60	171213_PT_H37Rv_2-2.sqt	25666	3	True	2461.292053	2461.297318	-2.13912	3.4778	13.9803852011545	0.5712	27	0.85316	N.LPYITVDADKNPLFLDEQLTR.A
61	171213_PT_H37Rv_2-2.sqt	37067	3	True	2998.528172	2998.53275	-1.52675	2.8112	8.28608147045285	0.5301	29	0.85249	R.AVGIDLGTTNSVVSVLEGGDPVVVANSEGSR.T
62	171213_PT_H37Rv_2-2.sqt	27075	4	True	3909.032858	3907.033505	-1.87949	4.6529	10.0519875608546	0.4877	46	0.85128	K.AKIELSSSQSTSINLPYITVDADKNPLFLDEQLTR.A
63	171213_PT_H37Rv_2-2.sqt	26709	3	True	2999.540928	2998.53275	1.60958	3.6122	10.8554054620534	0.5095	32	0.85102	R.AVGIDLGTTNSVVSVLEGGDPVVVANSEGSR.T
64	171213_PT_H37Rv_2-2.sqt	26909	4	True	4271.256491	4269.247355	0.57032	4.1619	7.86226722367649	0.3927	44	0.85067	M.ARAVGIDLGTTNSVVSVLEGGDPVVVANSEGSRTTPSIVAFAR.N
65	171213_PT_H37Rv_2-2.sqt	12707	4	True	3199.433737	3198.431867	-0.46258	5.0243	16.3500277083056	0.5868	43	0.85065	A.SQATGAAHPGGEPGGAHPGSADDVVDAEVVDDGR.E
66	171213_PT_H37Rv_2-2.sqt	17730	3	True	2636.184055	2635.174016	2.53738	3.0236	6.57845153183954	0.3888	21	0.84933	F.TT(+486.158400)ADDNQPSVQIQVYQGER.E

MS/MS Fragmentation of **TTADDNQPSVEIQVYQGER**
Found in **CND86259.1** in **NCBIprot**, Chaperone protein DnaK (Hsp 70) [Mycobacterium tuberculosis]

Match to Query 2: 2635.176779 from(2636.184055,1+) index(0)
Data file scans dnaK Hex-Hex-Hex Mascot.txt

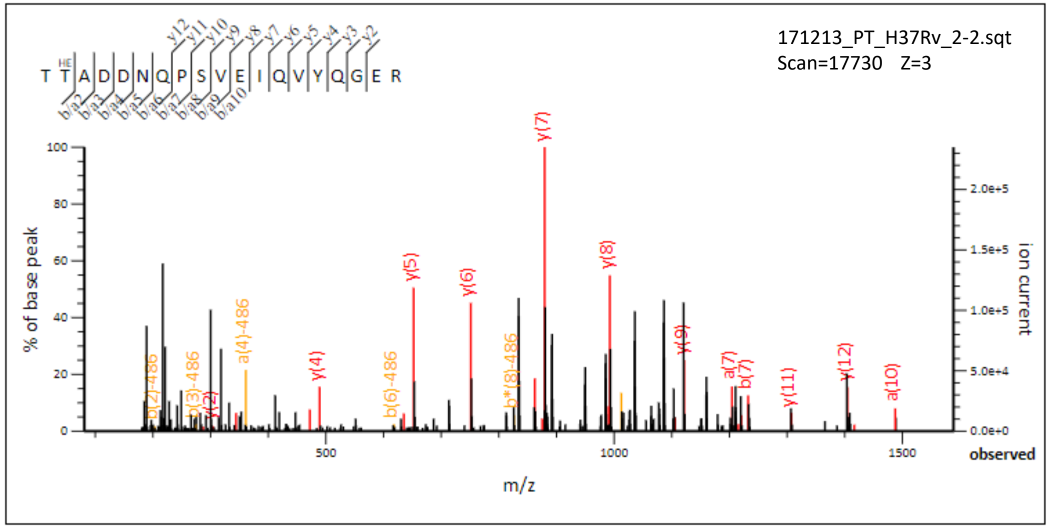

#	a	a*	b	b*	Seq.	y	y*	#
1	74.0600		102.0550		T			19
2	661.2662		689.2611		T	2535.1104	2518.0839	18
3	732.3033		760.2982		A	1947.9043	1930.8777	17
4	847.3302		875.3252		D	1876.8672	1859.8406	16
5	962.3572		990.3521		D	1761.8402	1744.8137	15
6	1076.4001	1059.3736	1104.3950	1087.3685	N	1646.8133	1629.7867	14
7	1204.4587	1187.4321	1232.4536	1215.4271	Q	1532.7703	1515.7438	13
8	1301.5115	1284.4849	1329.5064	1312.4798	P	1404.7118	1387.6852	12
9	1388.5435	1371.5169	1416.5384	1399.5119	S	1307.6590	1290.6325	11
10	1487.6119	1470.5854	1515.6068	1498.5803	V	1220.6270	1203.6004	10
11	1616.6545	1599.6279	1644.6494	1627.6229	E	1121.5586	1104.5320	9
12	1729.7386	1712.7120	1757.7335	1740.7069	I	992.5160	975.4894	8
13	1857.7971	1840.7706	1885.7921	1868.7655	Q	879.4319	862.4054	7
14	1956.8656	1939.8390	1984.8605	1967.8339	V	751.3733	734.3468	6
15	2119.9289	2102.9023	2147.9238	2130.8972	Y	652.3049	635.2784	5
16	2247.9875	2230.9609	2275.9824	2258.9558	Q	489.2416	472.2150	4
17	2305.0089	2287.9824	2333.0038	2315.9773	G	361.1830	344.1565	3
18	2434.0515	2417.0250	2462.0464	2445.0199	E	304.1615	287.1350	2
19					R	175.1190	158.0924	1

Probable transmembrane carbonic anhydrase Rv3273 (Rv3273)
Modification: Hex-Hex-Hex

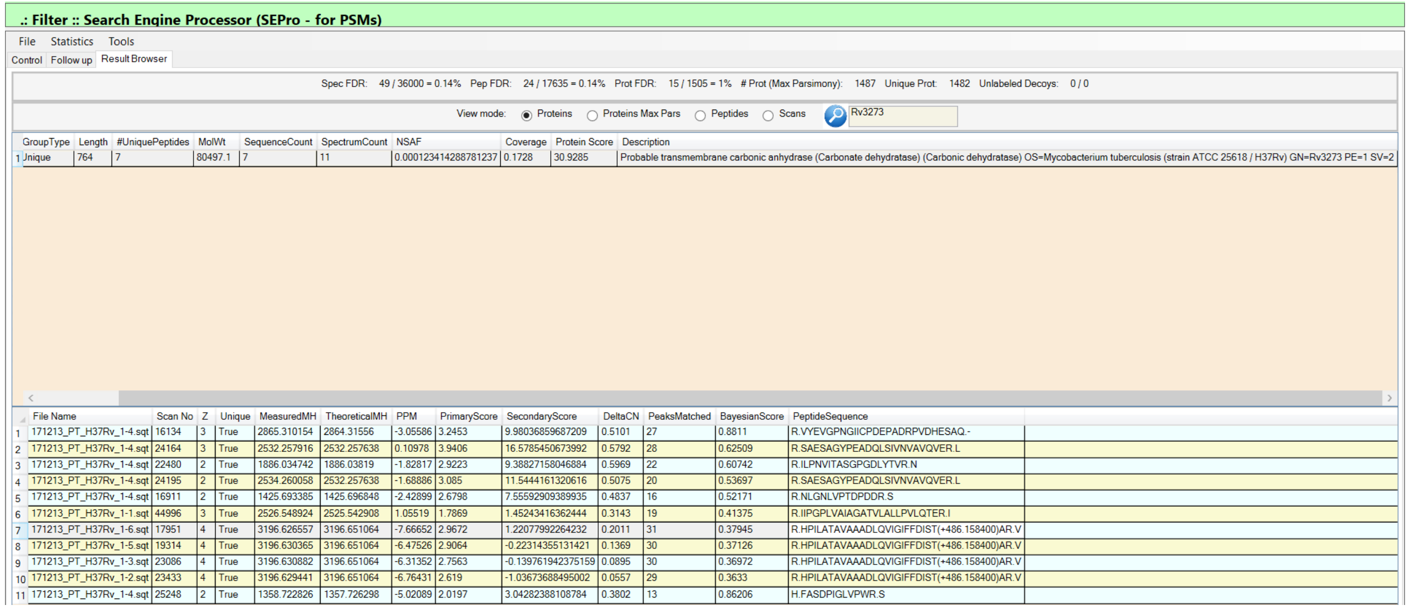

.: Filter :: Search Engine Processor (SEPro - for PSMs)

File Statistics Tools

Control Follow up Result Browser

Spec FDR: 49 / 36000 = 0.14% Pep FDR: 24 / 17635 = 0.14% Prot FDR: 15 / 1505 = 1% # Prot (Max Parsimony): 1487 Unique Prot: 1482 Unlabeled Decoys: 0 / 0

View mode: Proteins Proteins Max Pars Peptides Scans Rv3273

	GroupType	Length	#UniquePeptides	MolWt	SequenceCount	SpectrumCount	NSAF	Coverage	Protein Score	Description
1	Unique	764	7	80497.1	7	11	0.000123414288781237	0.1728	30.9285	Probable transmembrane carbonic anhydrase (Carbonate dehydratase) (Carbonic dehydratase) OS=Mycobacterium tuberculosis (strain ATCC 25618 / H37Rv) GN=Rv3273 PE=1 SV=2

	File Name	Scan No	Z	Unique	MeasuredMH	TheoreticalMH	PPM	PrimaryScore	SecondaryScore	DeltaCN	PeaksMatched	BayesianScore	PeptideSequence
1	171213_PT_H37Rv_1-4.sqt	16134	3	True	2865.310154	2864.31556	-3.05586	3.2453	9.98036859687209	0.5101	27	0.8811	R.VYEVGPNGIICPDEPADRPVDHESAQ.-
2	171213_PT_H37Rv_1-4.sqt	24164	3	True	2532.257916	2532.257638	0.10978	3.9406	16.5785450673992	0.5792	28	0.62509	R.SAESAGYPEADQLSIVNVAVQVER.L
3	171213_PT_H37Rv_1-4.sqt	22480	2	True	1886.034742	1886.03819	-1.82817	2.9223	9.38827158046884	0.5969	22	0.60742	R.ILPNVITASGPGDLYTVR.N
4	171213_PT_H37Rv_1-4.sqt	24195	2	True	2534.260058	2532.257638	-1.68886	3.085	11.5444161320616	0.5075	20	0.53697	R.SAESAGYPEADQLSIVNVAVQVER.L
5	171213_PT_H37Rv_1-4.sqt	16911	2	True	1425.693385	1425.696848	-2.42899	2.6798	7.55592909389935	0.4837	16	0.52171	R.NLGNLVPTDPDDR.S
6	171213_PT_H37Rv_1-1.sqt	44996	3	True	2526.548924	2525.542908	1.05519	1.7869	1.45243416362444	0.3143	19	0.41375	R.IIPGPLVAIAGATVLALLPVLQTER.I
7	171213_PT_H37Rv_1-6.sqt	17951	4	True	3196.626557	3196.651064	-7.66652	2.9672	1.22077992264232	0.2011	31	0.37945	R.HPILATAVAAADLQVIGIFFDIST(+486.158400)AR.V
8	171213_PT_H37Rv_1-5.sqt	19314	4	True	3196.630365	3196.651064	-6.47526	2.9064	-0.22314355131421	0.1369	30	0.37126	R.HPILATAVAAADLQVIGIFFDIST(+486.158400)AR.V
9	171213_PT_H37Rv_1-3.sqt	23086	4	True	3196.630882	3196.651064	-6.31352	2.7563	-0.139761942375159	0.0895	30	0.36972	R.HPILATAVAAADLQVIGIFFDIST(+486.158400)AR.V
10	171213_PT_H37Rv_1-2.sqt	23433	4	True	3196.629441	3196.651064	-6.76431	2.619	-1.03673688495002	0.0557	29	0.3633	R.HPILATAVAAADLQVIGIFFDIST(+486.158400)AR.V
11	171213_PT_H37Rv_1-4.sqt	25248	2	True	1358.722826	1357.726298	-5.02089	2.0197	3.04282388108784	0.3802	13	0.86206	H.FASDPIGLVPWR.S

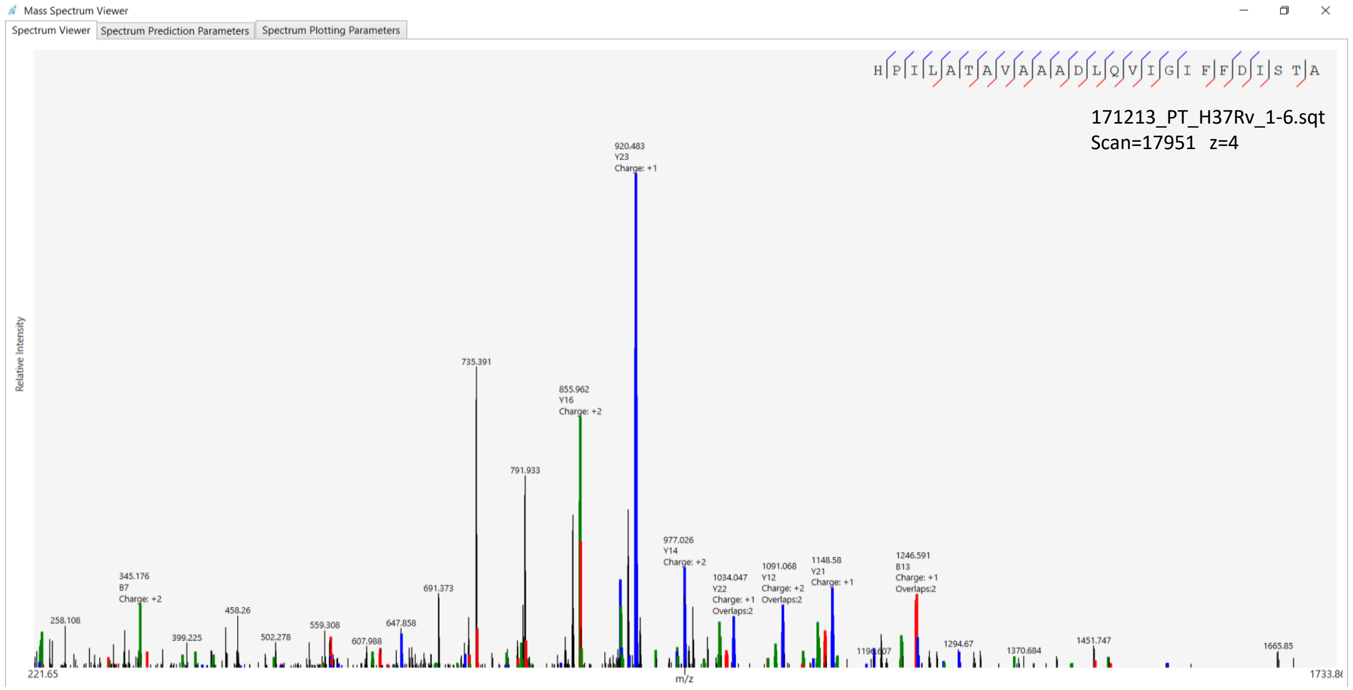

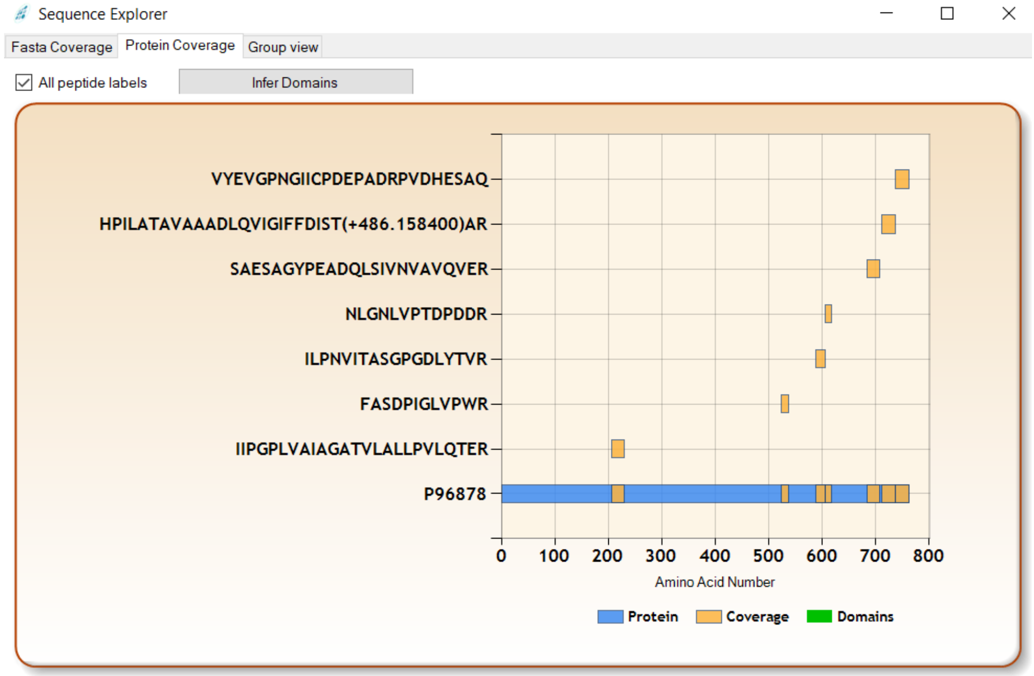

Probable isocitrate dehydrogenase [NADP] Icd2 (Rv0066c)
Modification: Hex-Hex-Hex

File Statistics Tools

Control Follow up Result Browser

Spec FDR: 49 / 36000 = 0.14% Pep FDR: 24 / 17635 = 0.14% Prot FDR: 15 / 1505 = 1% # Prot (Max Parsimony): 1487 Unique Prot: 1482 Unlabeled Decoys: 0 / 0

View mode: Proteins Proteins Max Pars Peptides Scans icd2

	Locus	Group	GroupType	Length	#UniquePeptides	MolWt	SequenceCount	SpectrumCount	NSAF	Coverage	Protein Score	Description
1	O53611	2	Unique	745	70	82479.6	70	143	0.00164530297473188	0.8148	426.9556	Isocitrate dehydrogenase OS=Mycobacterium tuberculosis (strain ATCC 25618 / H37Rv) GN=icd2 PE=1 SV=1

	File Name	Scan No	Z	Unique	MeasuredMH	TheoreticalMH	PPM	PrimaryScore	SecondaryScore	DeltaCN	PeaksMatched	BayesianScore	PeptideSequence
21	171213_PT_H37Rv_1-1.sqt	9824	3	True	1640.739505	1640.742041	-1.54564	2.5435	6.3367757323964	0.4812	26	0.52035	R.KHPHSMGEWSMASR.T
22	171213_PT_H37Rv_1-1.sqt	9850	4	True	1807.829272	1807.832643	-1.86467	2.8478	3.56489347433295	0.3102	26	0.40338	R.CHEHRPELAMVDSAR.G
23	171213_PT_H37Rv_1-1.sqt	9853	2	True	1640.741747	1640.742041	-0.17919	2.3621	11.4546565568463	0.6184	16	0.60275	R.KHPHSMGEWSMASR.T
24	171213_PT_H37Rv_1-1.sqt	9877	4	True	1640.739068	1640.742041	-1.81199	1.6825	0.0747235461959364	0.2233	24	0.37579	R.KHPHSMGEWSMASR.T
25	171213_PT_H37Rv_1-3.sqt	10270	2	True	1632.744163	1630.754078	-10.17612	1.6712	-1.30291275218084	0.0955	9	0.30473	K.LLDNDKSPS(+486.158400)R.K
26	171213_PT_H37Rv_1-2.sqt	10328	2	True	1632.745375	1630.754078	-9.4338	1.9178	0.619896718820353	0.1459	11	0.30923	K.LLDNDKSPS(+486.158400)R.K
27	171213_PT_H37Rv_1-2.sqt	10354	2	True	788.450629	788.451245	-0.78128	1.3248	-2.00821403239147	0.1581	9	0.3438	K.TLDAAIGK.L
28	171213_PT_H37Rv_1-1.sqt	10652	2	True	789.452379	788.451245	-2.80701	1.6396	1.8578992717326	0.2814	9	0.38279	K.TLDAAIGK.L
29	171213_PT_H37Rv_1-1.sqt	10681	2	True	788.450497	788.451245	-0.9487	1.4266	0.202116184122134	0.2568	9	0.35368	K.TLDAAIGK.L
30	171213_PT_H37Rv_1-2.sqt	11345	4	True	1318.734525	1318.737866	-2.53349	2.2022	2.44992568089275	0.2692	26	0.41477	K.VSHPIVFGHAVR.I
31	171213_PT_H37Rv_1-1.sqt	11452	4	True	1318.734595	1318.737866	-2.48041	2.4445	3.11226608980994	0.3324	26	0.4476	K.VSHPIVFGHAVR.I
32	171213_PT_H37Rv_1-1.sqt	11463	3	True	1318.736951	1318.737866	-0.69385	2.637	6.05333995082607	0.4563	20	0.51641	K.VSHPIVFGHAVR.I
33	171213_PT_H37Rv_1-1.sqt	11557	2	True	1318.735295	1318.737866	-1.9496	2.2498	4.6831317274578	0.3506	11	0.4507	K.VSHPIVFGHAVR.I
34	171213_PT_H37Rv_1-1.sqt	12496	2	True	1107.542331	1107.542914	-0.52639	2.3404	8.73410619297981	0.5606	12	0.58235	K.AVNPESTFSR.I

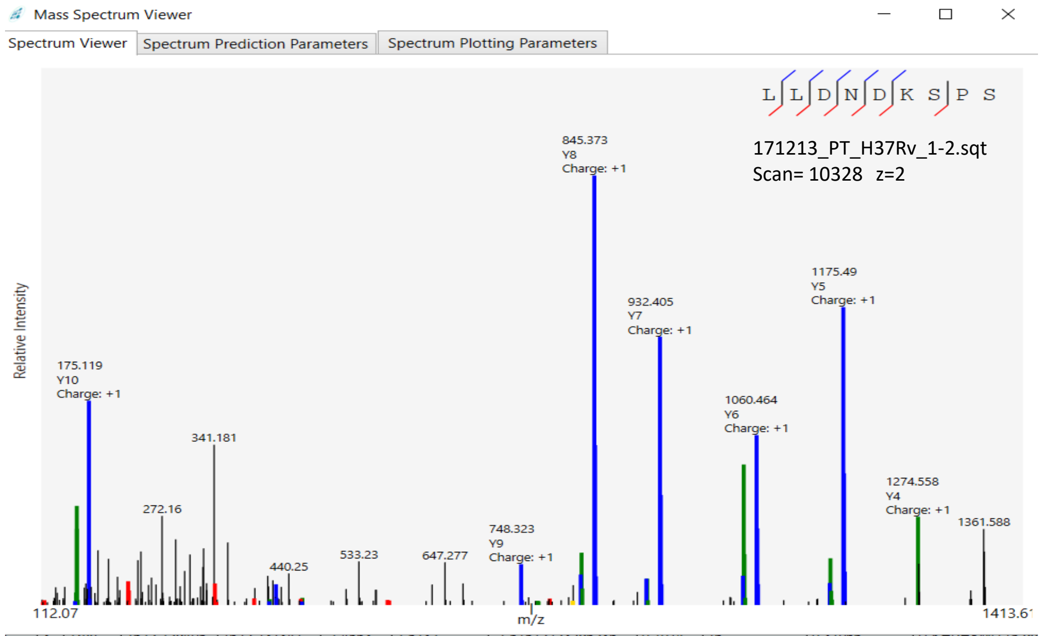

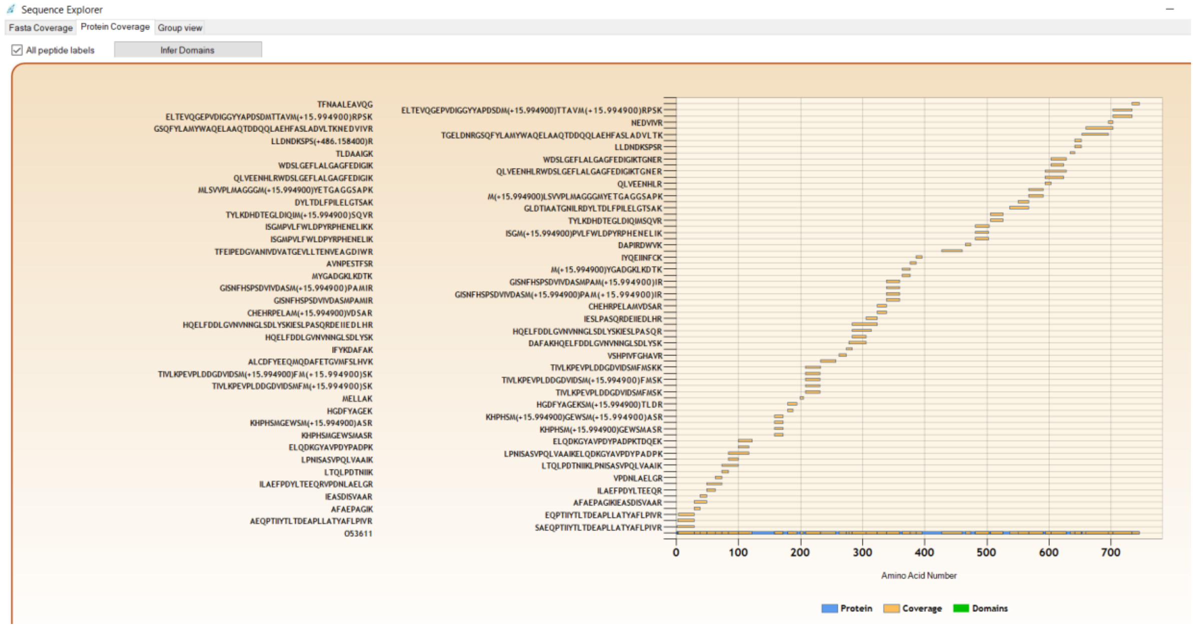

ESAT-6 like protein EsxC (Rv3890c)
Modification: Hex

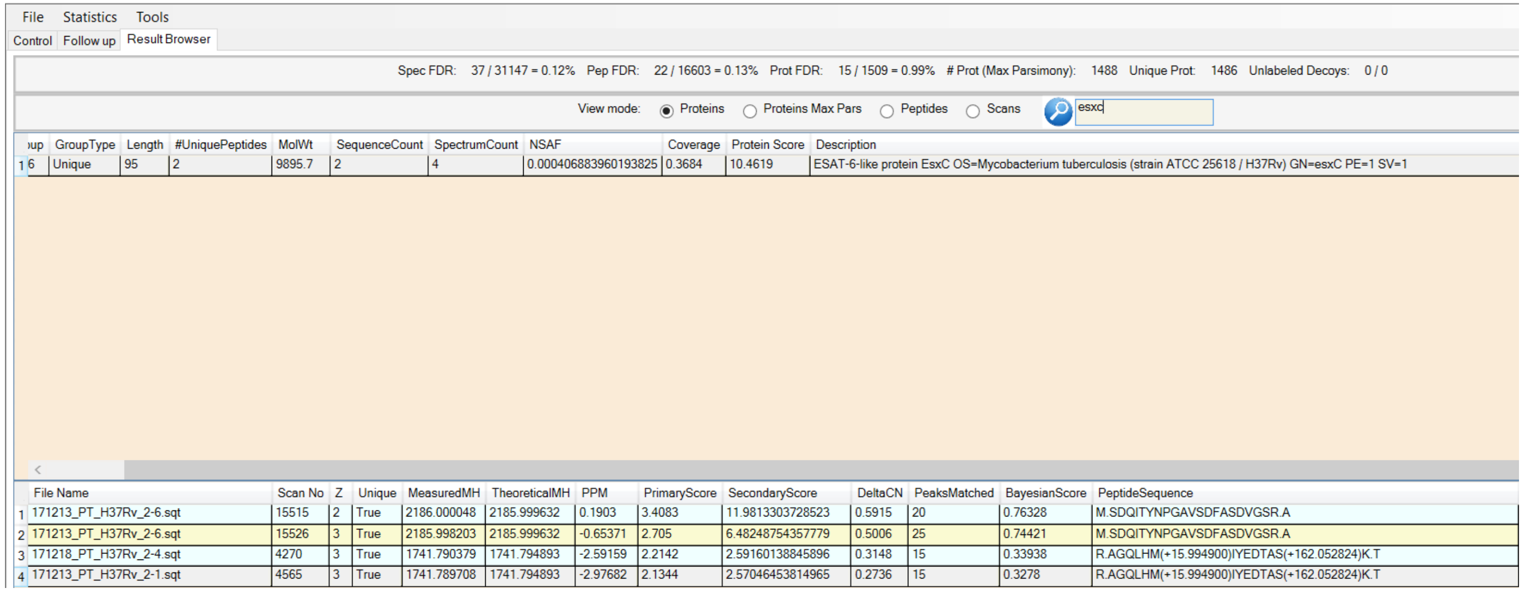

	up	GroupType	Length	#UniquePeptides	MolWt	SequenceCount	SpectrumCount	NSAF	Coverage	Protein Score	Description
1	6	Unique	95	2	9895.7	2	4	0.000406883960193825	0.3684	10.4619	ESAT-6-like protein EsxC OS=Mycobacterium tuberculosis (strain ATCC 25618 / H37Rv) GN=esxC PE=1 SV=1

	File Name	Scan No	Z	Unique	MeasuredMH	TheoreticalMH	PPM	PrimaryScore	SecondaryScore	DeltaCN	PeaksMatched	BayesianScore	PeptideSequence
1	171213_PT_H37Rv_2-6.sqt	15515	2	True	2186.000048	2185.999632	0.1903	3.4083	11.9813303728523	0.5915	20	0.76328	M.SDQITYNPGAVSDFASDVGSR.A
2	171213_PT_H37Rv_2-6.sqt	15526	3	True	2185.998203	2185.999632	-0.65371	2.705	6.48248754357779	0.5006	25	0.74421	M.SDQITYNPGAVSDFASDVGSR.A
3	171218_PT_H37Rv_2-4.sqt	4270	3	True	1741.790379	1741.794893	-2.59159	2.2142	2.59160138845896	0.3148	15	0.33938	R.AGQLHM(+15.994900)IYEDTAS(+162.052824)K.T
4	171213_PT_H37Rv_2-1.sqt	4565	3	True	1741.789708	1741.794893	-2.97682	2.1344	2.57046453814965	0.2736	15	0.3278	R.AGQLHM(+15.994900)IYEDTAS(+162.052824)K.T

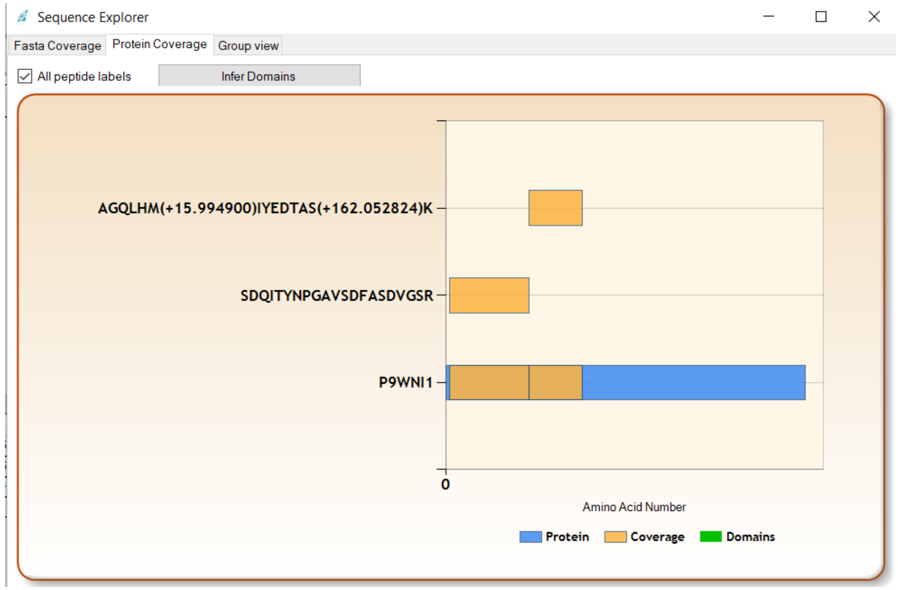

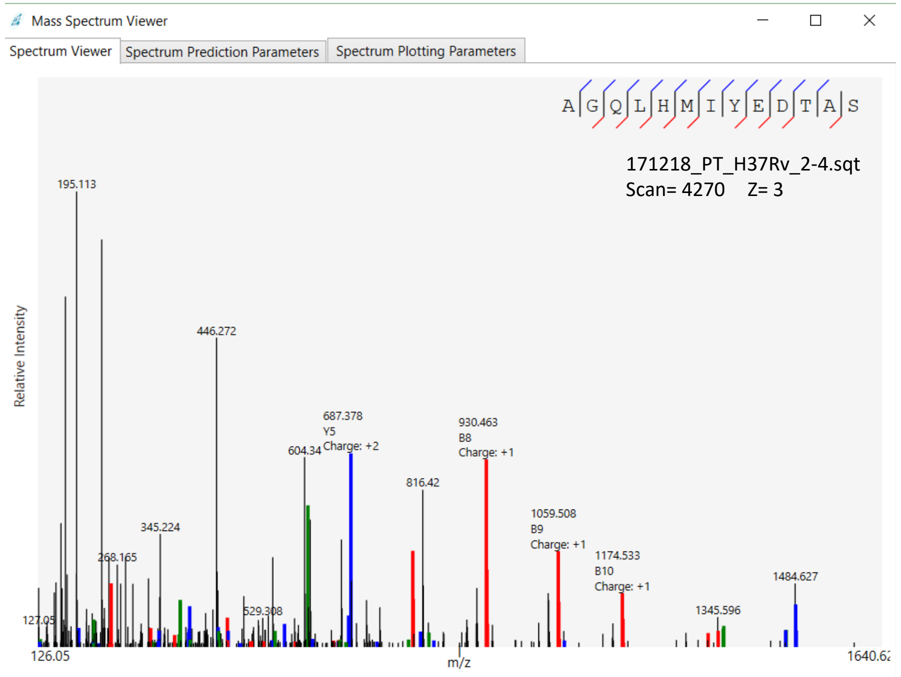

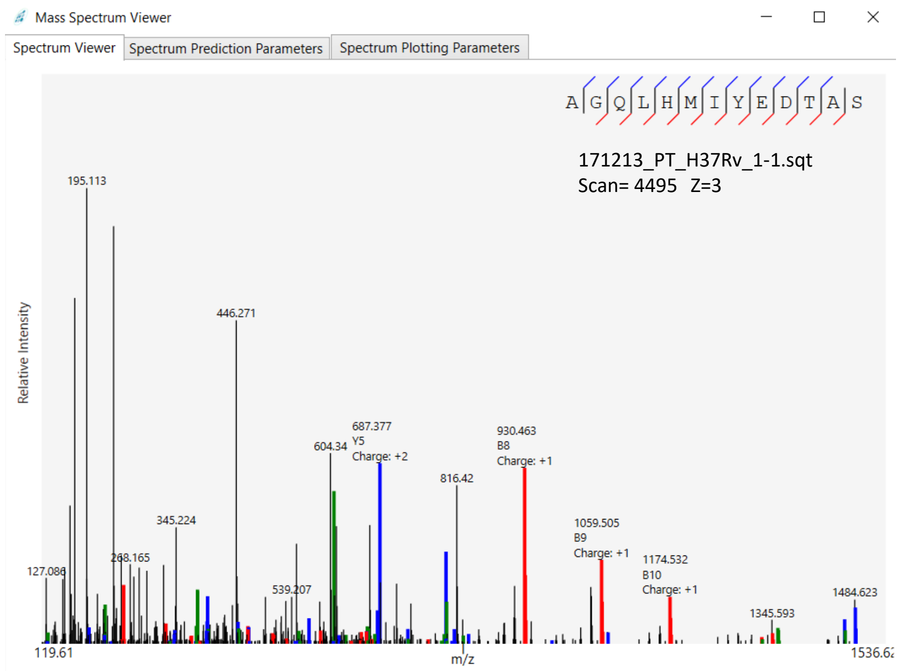

Probable adenosylhomocysteinase SahH (AhcY) (Rv3248c)
Modification: Hex

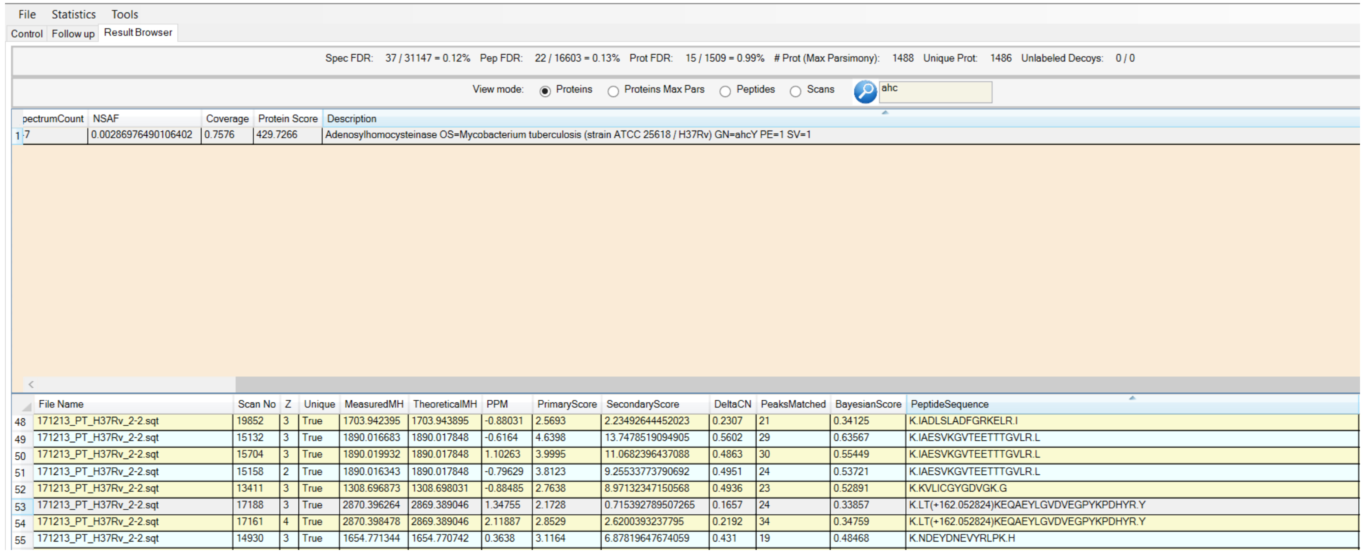
File Statistics Tools
Control Follow up Result Browser

Spec FDR: 37 / 31147 = 0.12% Pep FDR: 22 / 16603 = 0.13% Prot FDR: 15 / 1509 = 0.99% # Prot (Max Parsimony): 1488 Unique Prot: 1486 Unlabeled Decoys: 0 / 0

View mode: Proteins Proteins Max Pars Peptides Scans ahc

	pectrumCount	NSAF	Coverage	Protein Score	Description
1	7	0.00286976490106402	0.7576	429.7266	Adenosylhomocysteinase OS=Mycobacterium tuberculosis (strain ATCC 25618 / H37Rv) GN=ahcY PE=1 SV=1

	File Name	Scan No	Z	Unique	MeasuredMH	TheoreticalMH	PPM	PrimaryScore	SecondaryScore	DeltaCN	PeaksMatched	BayesianScore	PeptideSequence
48	171213_PT_H37Rv_2-2.sqt	19852	3	True	1703.942395	1703.943895	-0.88031	2.5693	2.23492644452023	0.2307	21	0.34125	K.IADLSLADFGRKELR.I
49	171213_PT_H37Rv_2-2.sqt	15132	3	True	1890.016683	1890.017848	-0.6164	4.6398	13.7478519094905	0.5602	29	0.63567	K.IAESVKGVTEETTTGVLR.L
50	171213_PT_H37Rv_2-2.sqt	15704	3	True	1890.019932	1890.017848	1.10263	3.9995	11.0682396437088	0.4863	30	0.55449	K.IAESVKGVTEETTTGVLR.L
51	171213_PT_H37Rv_2-2.sqt	15158	2	True	1890.016343	1890.017848	-0.79629	3.8123	9.25533773790692	0.4951	24	0.53721	K.IAESVKGVTEETTTGVLR.L
52	171213_PT_H37Rv_2-2.sqt	13411	3	True	1308.696873	1308.698031	-0.88485	2.7638	8.97132347150568	0.4936	23	0.52891	K.KVLICGYGDVGK.G
53	171213_PT_H37Rv_2-2.sqt	17188	3	True	2870.396264	2869.389046	1.34755	2.1728	0.715392789507265	0.1657	24	0.33857	K.LT(+162.052824)KEQAEYLGVDVEGPYKPDHYR.Y
54	171213_PT_H37Rv_2-2.sqt	17161	4	True	2870.398478	2869.389046	2.11887	2.8529	2.6200393237795	0.2192	34	0.34759	K.LT(+162.052824)KEQAEYLGVDVEGPYKPDHYR.Y
55	171213_PT_H37Rv_2-2.sqt	14930	3	True	1654.771344	1654.770742	0.3638	3.1164	6.87819647674059	0.431	19	0.48468	K.NDEYDNEVYRLPK.H

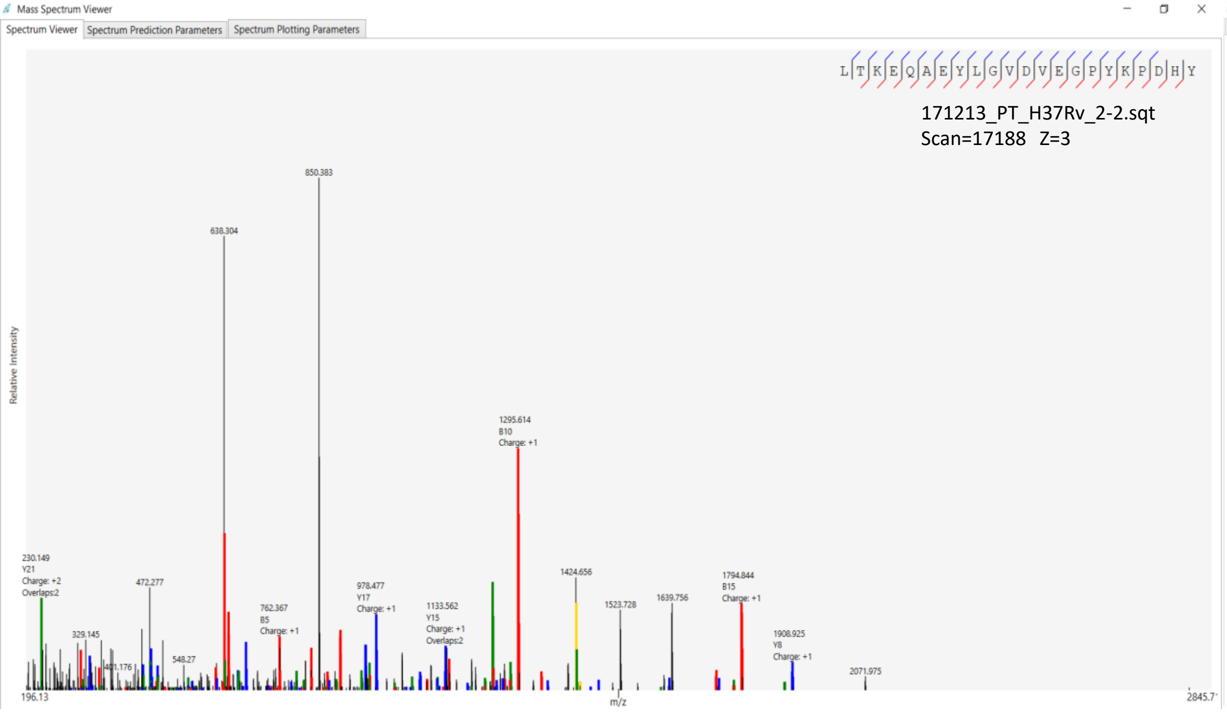

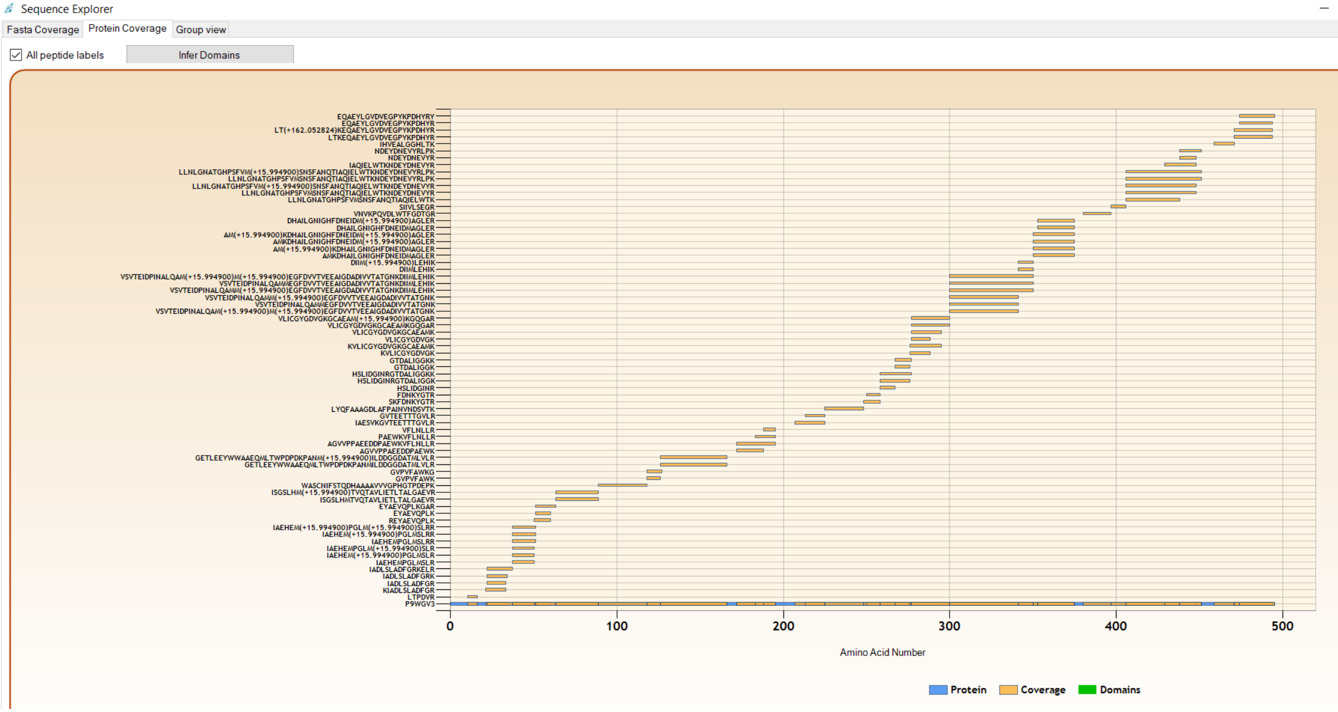

Probable cold-shock DeaD-box protein A homolog DeaD (Rv1253)
Modification: Hex

File Statistics Tools

Control | Follow up | Result Browser

Spec FDR: 37 / 31147 = 0.12% Pep FDR: 22 / 16603 = 0.13% Prot FDR: 15 / 1509 = 0.99% # Prot (Max Parsimony): 1488 Unique Prot: 1486 Unlabeled Decoys: 0 / 0

View mode: (•) Proteins ( ) Proteins Max Pars ( ) Peptides ( ) Scans dead

	Locus	Group	GroupType	Length	#UniquePeptides	MolWt	SequenceCount	SpectrumCount	NSAF	Coverage	Protein Score	Description
1	P9WH05	340	Unique	563	3	61396.6	3	5	8.58214392060687E-05	0.0515	12.2048	ATP-dependent RNA helicase DeaD OS=Mycobacterium tuberculosis (strain ATCC 25618 / H37Rv) GN=deaD PE=1 SV=1

	File Name	Scan No	Z	Unique	MeasuredMH	TheoreticalMH	PPM	PrimaryScore	SecondaryScore	DeltaCN	PeaksMatched	BayesianScore	PeptideSequence
1	171213_PT_H37Rv_2-2.sqt	26209	3	True	2260.216892	2260.203083	6.10959	3.6222	9.04482593349861	0.4626	29	0.73782	T.IT(+162.052824)ALRDGDIDILVATDVAAR.G
2	171213_PT_H37Rv_2-1.sqt	27626	3	True	2261.225071	2260.203083	8.24243	3.1385	7.3697907385787	0.4117	23	0.71018	T.IT(+162.052824)ALRDGDIDILVATDVAAR.G
3	171213_PT_H37Rv_2-2.sqt	11311	2	True	1181.564388	1180.557955	2.60925	1.7886	0.805196684368568	0.2194	11	0.3386	K.QAT(+162.052824)EEIAEK.L
4	171213_PT_H37Rv_2-1.sqt	11837	2	True	1181.56621	1180.557955	4.15127	1.627	-1.18172719537862	0.0711	11	0.31228	K.QAT(+162.052824)EEIAEK.L
5	171213_PT_H37Rv_2-1.sqt	14580	2	True	1028.608446	1028.609871	-1.38537	2.0285	0.959720289801491	0.3592	12	0.68362	D.ILVATDVAAR.G

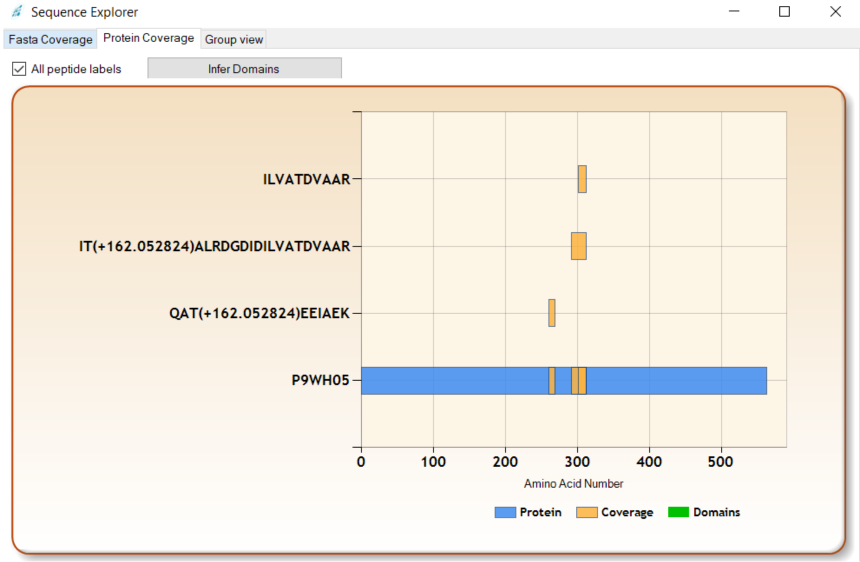

MS/MS Fragmentation of **ITALRDGDIDILVATDVAAR**
Found in **CNW03826.1** in **NCBIprot**, cold-shock DEAD-box protein A [Mycobacterium tuberculosis]

Match to Query 2: 2259.209616 from(2260.216892,1+) index(1)
Data file scans DeaD Hex Mascot.txt

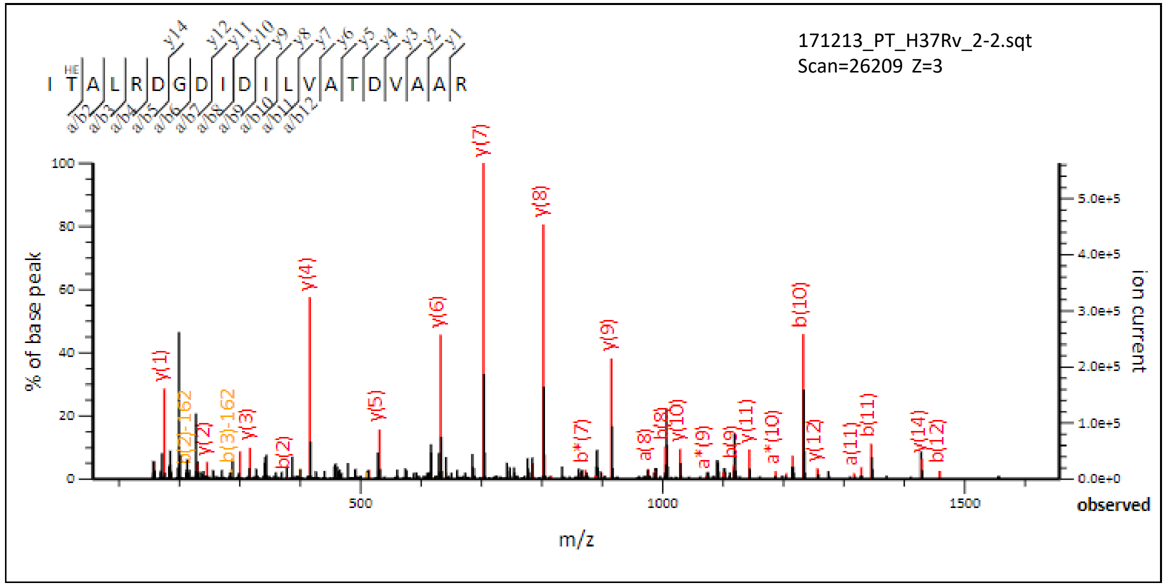

#	a	a*	b	b*	Seq.	y	y*	#
1	86.0964		114.0913		I			20
2	349.1969		377.1918		T	2147.1190	2130.0925	19
3	420.2340		448.2290		A	1884.0185	1866.9920	18
4	533.3181		561.3130		L	1812.9814	1795.9549	17
5	689.4192	672.3927	717.4141	700.3876	R	1699.8973	1682.8708	16
6	804.4462	787.4196	832.4411	815.4145	D	1543.7962	1526.7697	15
7	861.4676	844.4411	889.4625	872.4360	G	1428.7693	1411.7427	14
8	976.4946	959.4680	1004.4895	987.4629	D	1371.7478	1354.7213	13
9	1089.5786	1072.5521	1117.5735	1100.5470	I	1256.7209	1239.6943	12
10	1204.6056	1187.5790	1232.6005	1215.5739	D	1143.6368	1126.6103	11
11	1317.6896	1300.6631	1345.6846	1328.6580	I	1028.6099	1011.5833	10
12	1430.7737	1413.7472	1458.7686	1441.7421	L	915.5258	898.4993	9
13	1529.8421	1512.8156	1557.8370	1540.8105	V	802.4417	785.4152	8
14	1600.8792	1583.8527	1628.8741	1611.8476	A	703.3733	686.3468	7
15	1701.9269	1684.9004	1729.9218	1712.8953	T	632.3362	615.3097	6
16	1816.9539	1799.9273	1844.9488	1827.9222	D	531.2885	514.2620	5
17	1916.0223	1898.9957	1944.0172	1926.9906	V	416.2616	399.2350	4
18	1987.0594	1970.0328	2015.0543	1998.0277	A	317.1932	300.1666	3
19	2058.0965	2041.0699	2086.0914	2069.0649	A	246.1561	229.1295	2
20					R	175.1190	158.0924	1

Probable polyketide synthase Pks5 (Rv1527C)
Modification: Hex-Hex-Hex

File Statistics Tools

Control | Follow up | Result Browser

Spec FDR: 49 / 36000 = 0.14% Pep FDR: 24 / 17635 = 0.14% Prot FDR: 15 / 1505 = 1% # Prot (Max Parsimony): 1487 Unique Prot: 1482 Unlabeled Decoys: 0 / 0

View mode: ◉ Proteins ○ Proteins Max Pars ○ Peptides ○ Scans pks5

	Locus	Group	GroupType	Length	#UniquePeptides	MolWt	SequenceCount	SpectrumCount	NSAF	Coverage	Protein Score	Description
1	O53901	1399	Some	2108	14	223731.9	21	24	9.759032254152E-05	0.1565	69.2663	Mycocerosic acid synthase-like polyketide synthase OS=Mycobacterium tuberculosis (strain ATCC 25618 / H37Rv) GN=pks5 PE=1 SV=2

	File Name	Scan No	Z	Unique	MeasuredMH	TheoreticalMH	PPM	PrimaryScore	SecondaryScore	DeltaCN	PeaksMatched	BayesianScore	PeptideSequence
1	171213_PT_H37Rv_1-1.sqt	26484	3	True	3202.624851	3201.617483	1.2546	3.9814	14.927208086181	0.588	30	0.62926	R.LRDQLGTGGFTGVFVLTAPQTGDPDAESPVR.G
2	171213_PT_H37Rv_1-1.sqt	37697	3	True	3168.669773	3166.678292	-4.80296	4.4336	13.6585068091546	0.5782	34	0.60394	R.LGPAFAGLVDAYIAEGAGDTVLAEVNLPGPLR.S
3	171213_PT_H37Rv_1-1.sqt	35476	2	True	2334.233446	2333.234717	-1.97966	3.5114	13.2731862671389	0.5838	20	0.61053	R.DTAGYGVDIVLNSVTGAAQLAGLK.L
4	171213_PT_H37Rv_1-1.sqt	32620	3	True	2283.178318	2282.18156	-2.8872	4.4933	12.8334320855521	0.5772	34	0.62047	R.ALAGDLYAAGAAVDFAVLYPTGR.L
5	171213_PT_H37Rv_1-1.sqt	32797	4	True	3118.620749	3117.621506	-1.31693	5.4759	12.3136578562101	0.5409	38	0.60693	R.GVLHAAAVVEDATLANITDELIERDWAPK.A
6	171213_PT_H37Rv_1-1.sqt	25327	3	False	1864.073914	1864.076307	-1.28375	3.8732	10.9140889638815	0.5816	30	0.62143	K.RLPDALADGDRILAVIR.G
7	171213_PT_H37Rv_1-1.sqt	37624	3	True	3167.68137	3166.678292	-0.08587	4.2994	10.860602789305	0.5488	36	0.59866	R.LGPAFAGLVDAYIAEGAGDTVLAEVNLPGPLR.S
8	171213_PT_H37Rv_1-1.sqt	30620	2	True	2457.251024	2456.245616	0.83752	4.0152	8.61701352669845	0.4888	24	0.54504	R.LADWVDAQGPELAPADLAYTLAR.R
9	171213_PT_H37Rv_1-1.sqt	32293	3	True	2756.356175	2754.362103	-4.58141	3.1139	8.44023215028011	0.4666	26	0.50612	R.AIAFAEQTGDAIAPEEGAYAFETLLR.H
10	171213_PT_H37Rv_1-1.sqt	12190	2	True	1374.693773	1374.697183	-2.48055	2.2444	6.63772814176908	0.4112	11	0.41716	R.GLQLGTGASQASER.A
11	171213_PT_H37Rv_1-1.sqt	33012	3	True	2486.22855	2486.214365	5.70543	3.1085	6.53619172254965	0.3578	20	0.86098	F.T(+486.158400)ELTPHPLLTHAVDQTAR.S

Sequence Explorer

Fasta Coverage | Protein Coverage | Group view

☑ All peptide labels Infer Domains

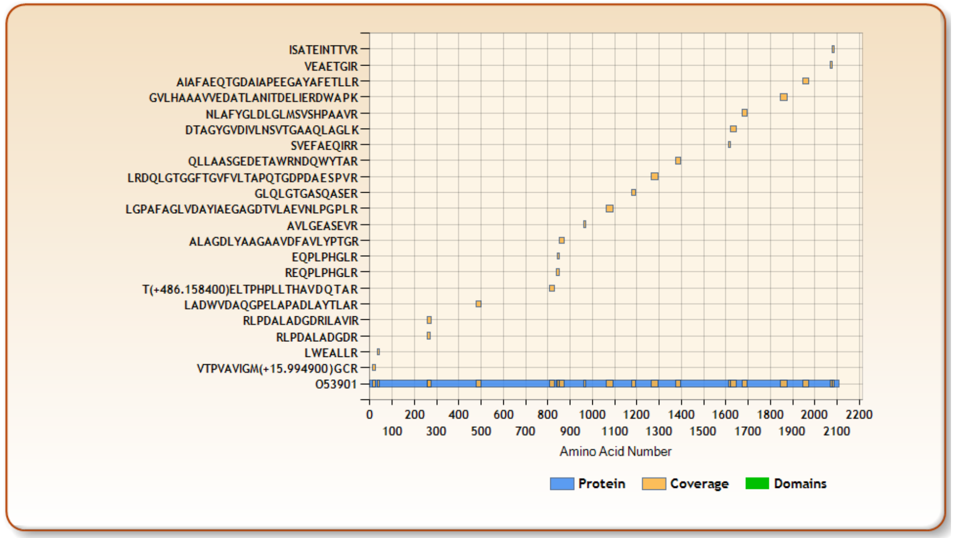

MS/MS Fragmentation of **TELTPHPLLTHAVDQTAR**
Found in **WP_003901190.1** in **NCBIprot**, type I polyketide synthase [Mycobacterium tuberculosis]

Match to Query 1: 2485.221274 from(2486.228550,1+) index(1)
Data file scans pks5 Mascot.txt

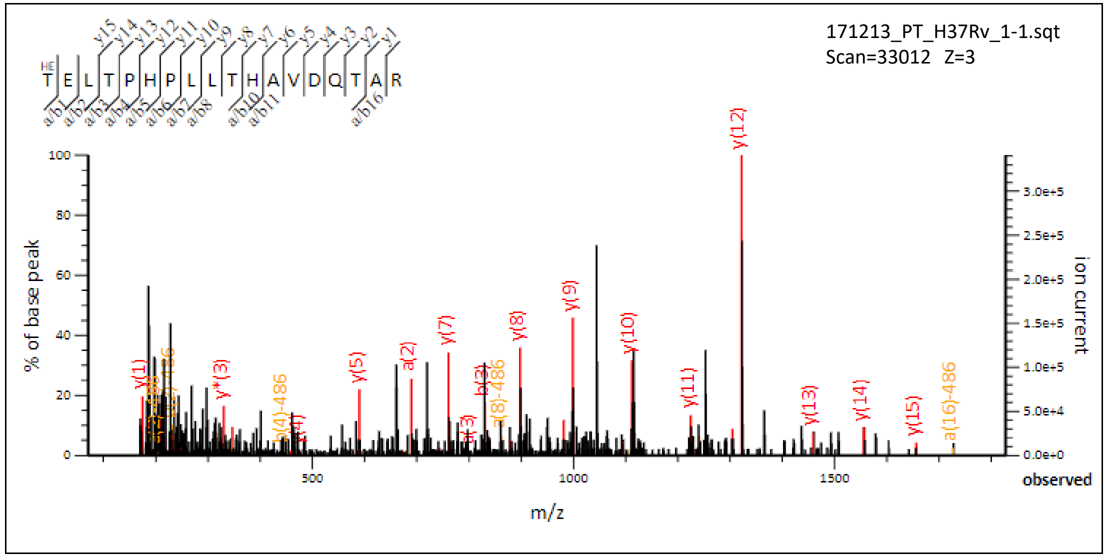

#	a	a*	b	b*	Seq.	y	y*	#
1	560.2185		588.2134		T			18
2	689.2611		717.2560		E	1899.0083	1881.9817	17
3	802.3452		830.3401		L	1769.9657	1752.9391	16
4	903.3928		931.3878		T	1656.8816	1639.8551	15
5	1000.4456		1028.4405		P	1555.8340	1538.8074	14
6	1137.5045		1165.4994		H	1458.7812	1441.7546	13
7	1234.5573		1262.5522		P	1321.7223	1304.6957	12
8	1347.6414		1375.6363		L	1224.6695	1207.6430	11
9	1460.7254		1488.7203		L	1111.5854	1094.5589	10
10	1561.7731		1589.7680		T	998.5014	981.4748	9
11	1698.8320		1726.8269		H	897.4537	880.4272	8
12	1769.8691		1797.8640		A	760.3948	743.3682	7
13	1868.9375		1896.9324		V	689.3577	672.3311	6
14	1983.9645		2011.9594		D	590.2893	573.2627	5
15	2112.0231	2094.9965	2140.0180	2122.9914	Q	475.2623	458.2358	4
16	2213.0707	2196.0442	2241.0656	2224.0391	T	347.2037	330.1772	3
17	2284.1078	2267.0813	2312.1028	2295.0762	A	246.1561	229.1295	2
18					R	175.1190	158.0924	1

Diviva family protein Wag31 (Rv2145c)
Modification: Hex

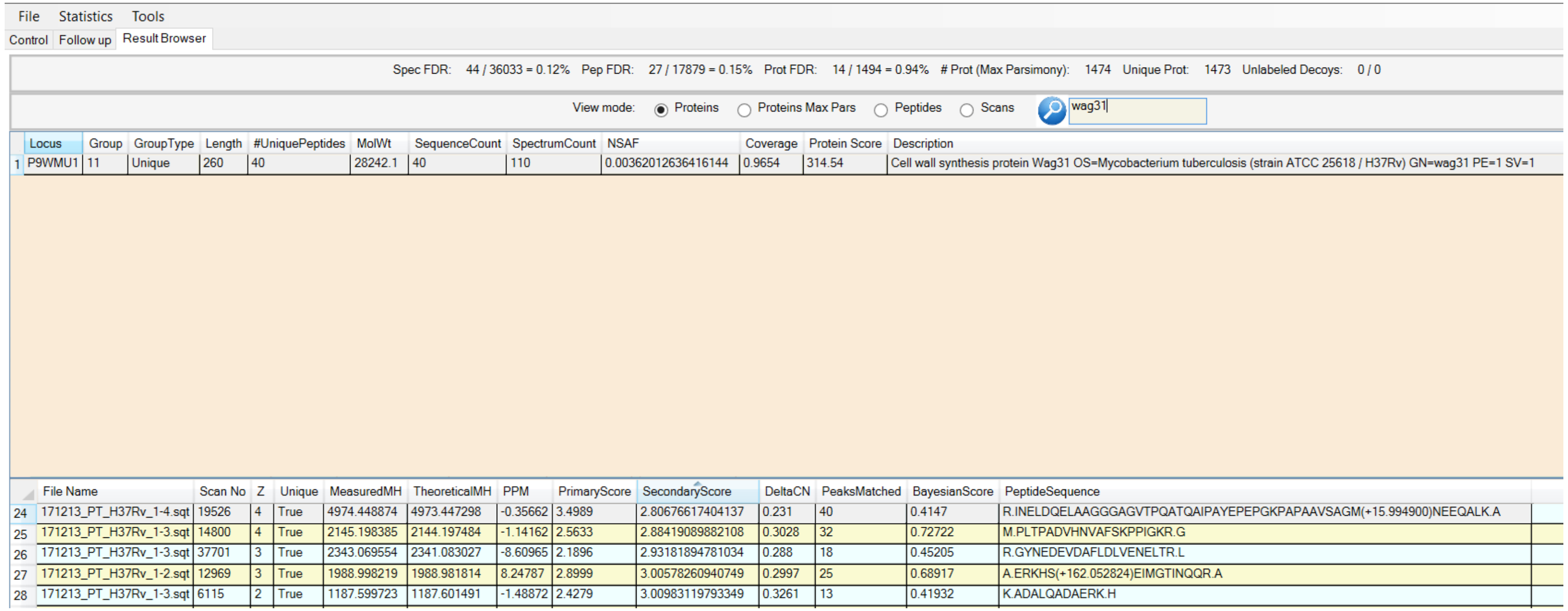

File Statistics Tools

Control Follow up Result Browser

Spec FDR: 44 / 36033 = 0.12% Pep FDR: 27 / 17879 = 0.15% Prot FDR: 14 / 1494 = 0.94% # Prot (Max Parsimony): 1474 Unique Prot: 1473 Unlabeled Decoys: 0 / 0

View mode: Proteins Proteins Max Pars Peptides Scans wag31

	Locus	Group	GroupType	Length	#UniquePeptides	MolWt	SequenceCount	SpectrumCount	NSAF	Coverage	Protein Score	Description
1	P9WMU1	11	Unique	260	40	28242.1	40	110	0.00362012636416144	0.9654	314.54	Cell wall synthesis protein Wag31 OS=Mycobacterium tuberculosis (strain ATCC 25618 / H37Rv) GN=wag31 PE=1 SV=1

	File Name	Scan No	Z	Unique	MeasuredMH	TheoreticalMH	PPM	PrimaryScore	SecondaryScore	DeltaCN	PeaksMatched	BayesianScore	PeptideSequence
24	171213_PT_H37Rv_1-4.sqt	19526	4	True	4974.448874	4973.447298	-0.35662	3.4989	2.80676617404137	0.231	40	0.4147	R.INELDQELAAGGGAGVTPQATQAIPAYEPEPGKPAPAAVSAGM(+15.994900)NEEQALK.A
25	171213_PT_H37Rv_1-3.sqt	14800	4	True	2145.198385	2144.197484	-1.14162	2.5633	2.88419089882108	0.3028	32	0.72722	M.PLTPADVHNVAFSKPPIGKR.G
26	171213_PT_H37Rv_1-3.sqt	37701	3	True	2343.069554	2341.083027	-8.60965	2.1896	2.93181894781034	0.288	18	0.45205	R.GYNEDEVDAFLDLVENELTR.L
27	171213_PT_H37Rv_1-2.sqt	12969	3	True	1988.998219	1988.981814	8.24787	2.8999	3.00578260940749	0.2997	25	0.68917	A.ERKHS(+162.052824)EIMGTINQQR.A
28	171213_PT_H37Rv_1-3.sqt	6115	2	True	1187.599723	1187.601491	-1.48872	2.4279	3.00983119793349	0.3261	13	0.41932	K.ADALQADAERK.H

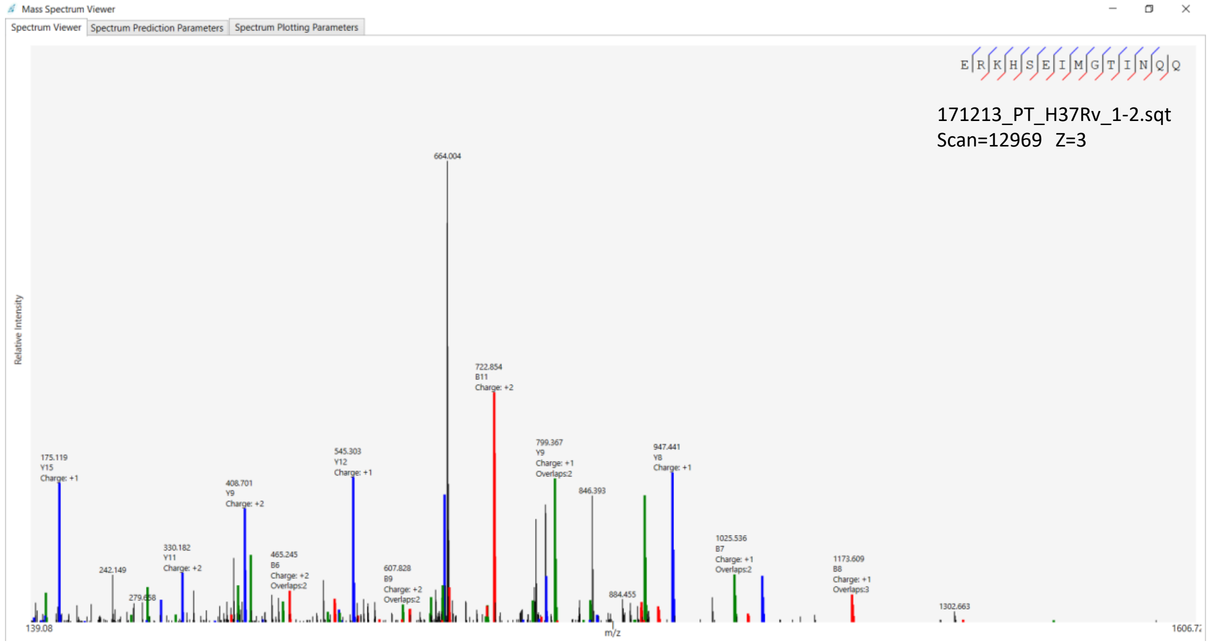

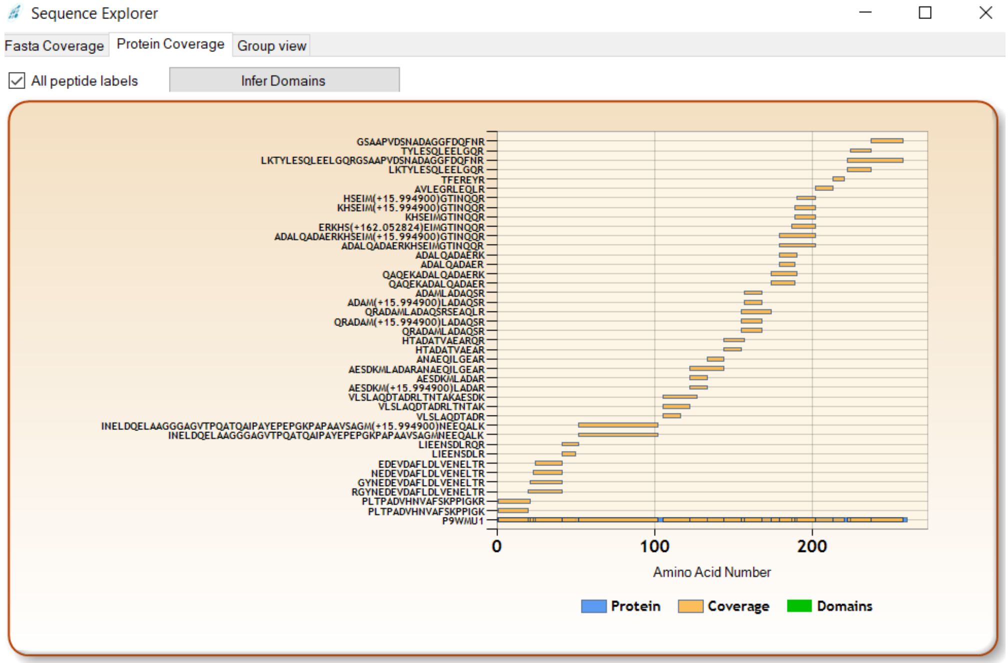

Glutamine synthetase GlnA1 (glutamine synthase) (Rv2220)
Modification: Hex

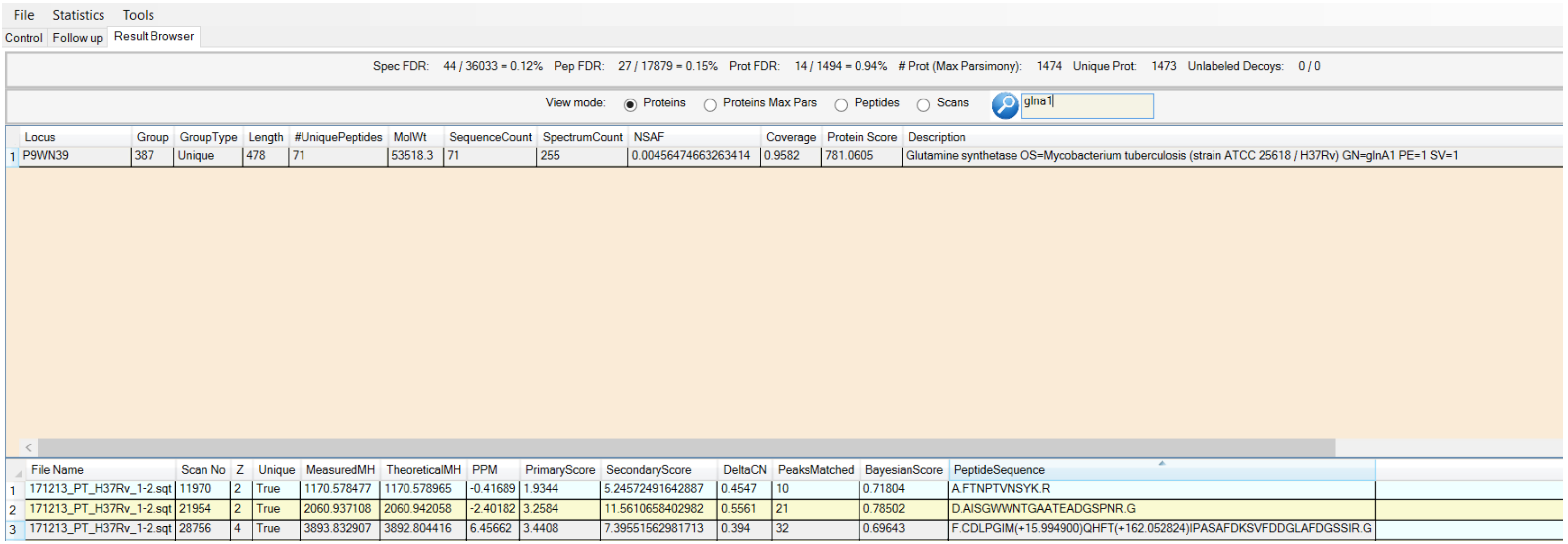

File Statistics Tools

Control Follow up Result Browser

Spec FDR: 44 / 36033 = 0.12% Pep FDR: 27 / 17879 = 0.15% Prot FDR: 14 / 1494 = 0.94% # Prot (Max Parsimony): 1474 Unique Prot: 1473 Unlabeled Decoys: 0 / 0

View mode: Proteins Proteins Max Pars Peptides Scans glna1

	Locus	Group	GroupType	Length	#UniquePeptides	MolWt	SequenceCount	SpectrumCount	NSAF	Coverage	Protein Score	Description
1	P9WN39	387	Unique	478	71	53518.3	71	255	0.00456474663263414	0.9582	781.0605	Glutamine synthetase OS=Mycobacterium tuberculosis (strain ATCC 25618 / H37Rv) GN=glnA1 PE=1 SV=1

	File Name	Scan No	Z	Unique	MeasuredMH	TheoreticalMH	PPM	PrimaryScore	SecondaryScore	DeltaCN	PeaksMatched	BayesianScore	PeptideSequence
1	171213_PT_H37Rv_1-2.sqt	11970	2	True	1170.578477	1170.578965	-0.41689	1.9344	5.24572491642887	0.4547	10	0.71804	A.FTNPTVNSYK.R
2	171213_PT_H37Rv_1-2.sqt	21954	2	True	2060.937108	2060.942058	-2.40182	3.2584	11.5610658402982	0.5561	21	0.78502	D.AISGWWNTGAATEADGSPNR.G
3	171213_PT_H37Rv_1-2.sqt	28756	4	True	3893.832907	3892.804416	6.45662	3.4408	7.39551562981713	0.394	32	0.69643	F.CDLPGIM(+15.994900)QHFT(+162.052824)IPASAFDKSVFDDGLAFDGSSIR.G

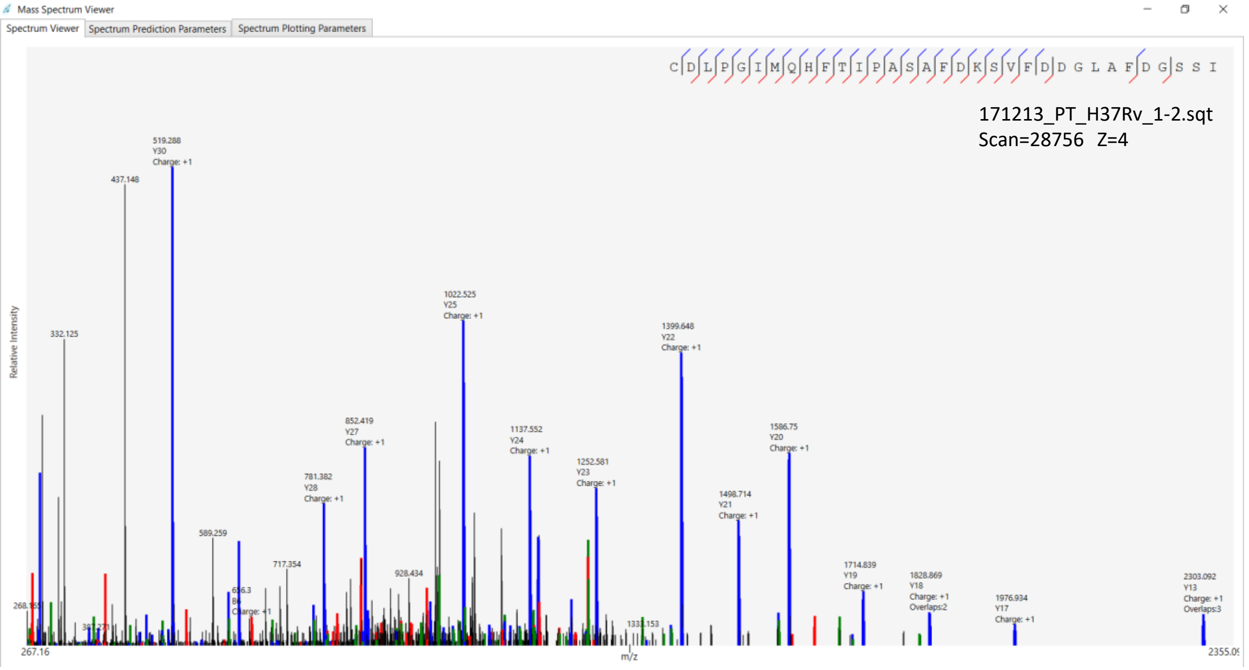

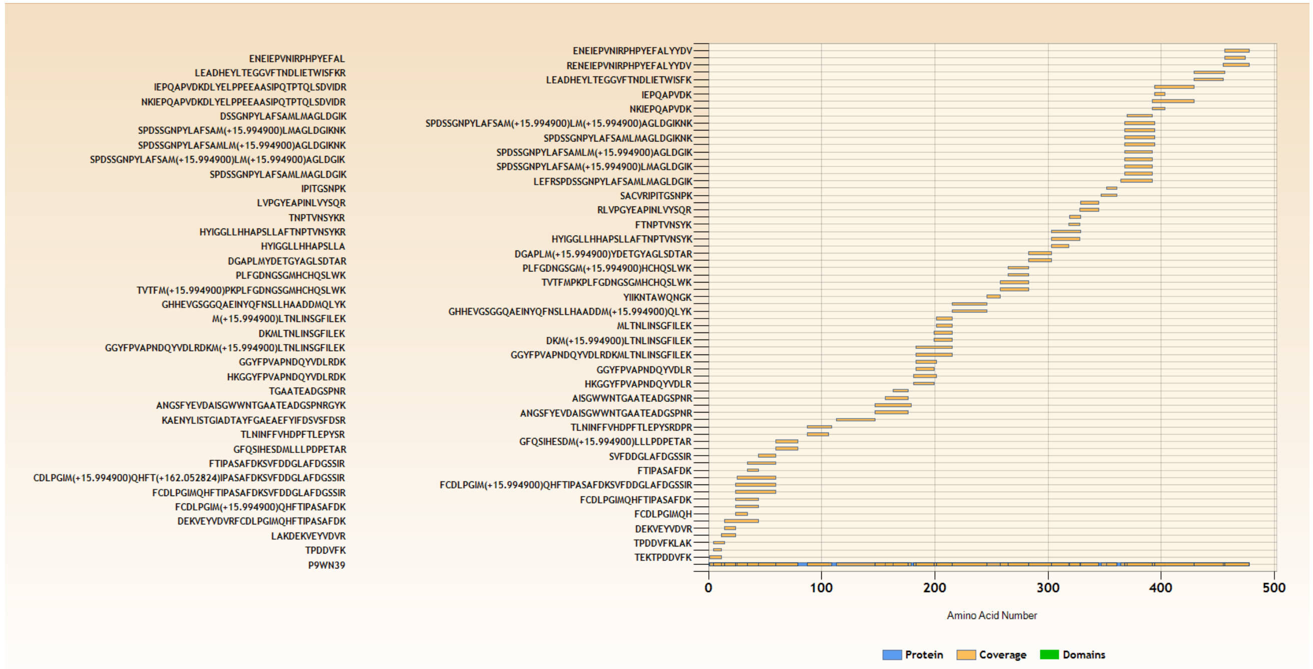

Pyruvate dehydrogenase E1 component AceE (Rv2241)
Modification: Hex-Hex

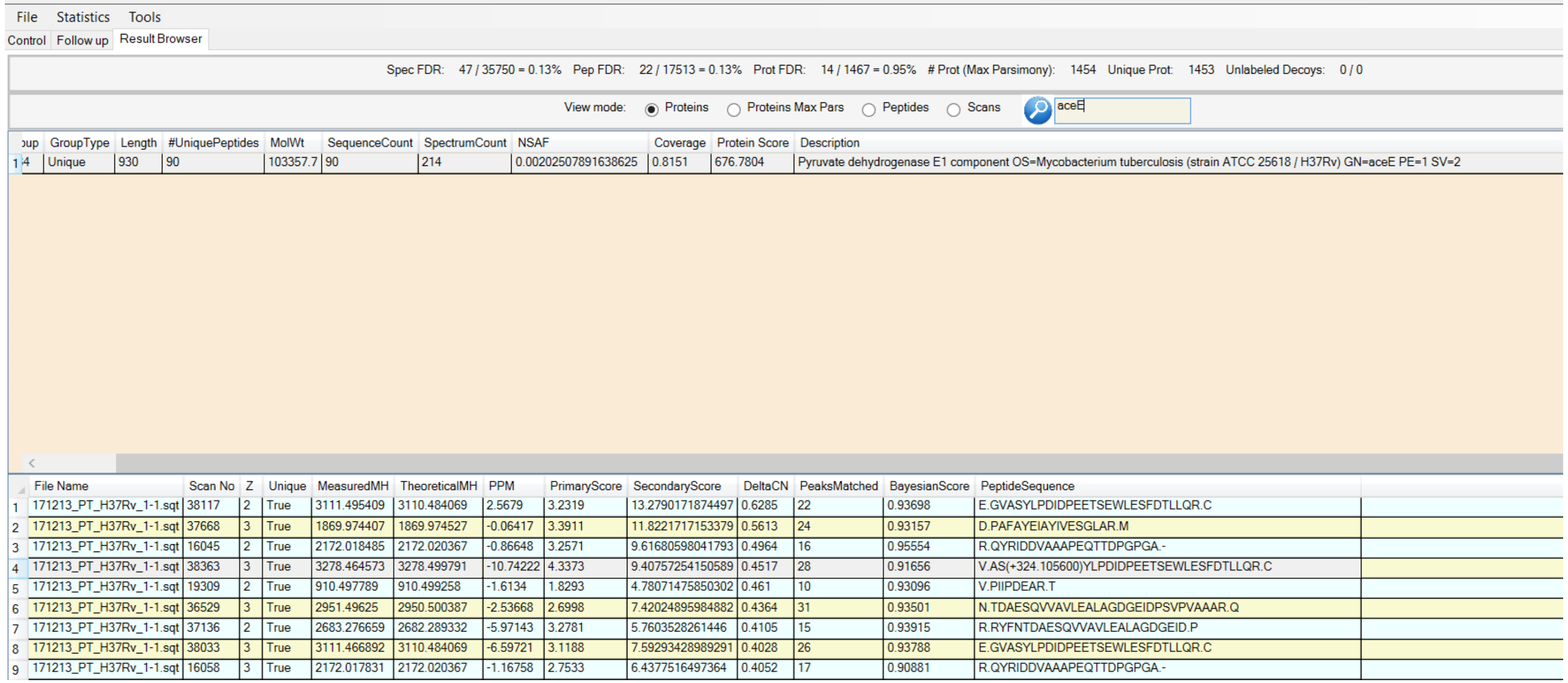

	oup	GroupType	Length	#UniquePeptides	MolWt	SequenceCount	SpectrumCount	NSAF	Coverage	Protein Score	Description
1	4	Unique	930	90	103357.7	90	214	0.00202507891638625	0.8151	676.7804	Pyruvate dehydrogenase E1 component OS=Mycobacterium tuberculosis (strain ATCC 25618 / H37Rv) GN=aceE PE=1 SV=2

	File Name	Scan No	Z	Unique	MeasuredMH	TheoreticalMH	PPM	PrimaryScore	SecondaryScore	DeltaCN	PeaksMatched	BayesianScore	PeptideSequence
1	171213_PT_H37Rv_1-1.sqt	38117	2	True	3111.495409	3110.484069	2.5679	3.2319	13.2790171874497	0.6285	22	0.93698	E.GVASYLPDIDPEETSEWLESFDTLLQR.C
2	171213_PT_H37Rv_1-1.sqt	37668	3	True	1869.974407	1869.974527	-0.06417	3.3911	11.8221717153379	0.5613	24	0.93157	D.PAFAYEIAYIVESGLAR.M
3	171213_PT_H37Rv_1-1.sqt	16045	2	True	2172.018485	2172.020367	-0.86648	3.2571	9.61680598041793	0.4964	16	0.95554	R.QYRIDDVAAAPEQTTDPGPGA.-
4	171213_PT_H37Rv_1-1.sqt	38363	3	True	3278.464573	3278.499791	-10.74222	4.3373	9.40757254150589	0.4517	28	0.91656	V.AS(+324.105600)YLPDIDPEETSEWLESFDTLLQR.C
5	171213_PT_H37Rv_1-1.sqt	19309	2	True	910.497789	910.499258	-1.6134	1.8293	4.78071475850302	0.461	10	0.93096	V.PIIPDEAR.T
6	171213_PT_H37Rv_1-1.sqt	36529	3	True	2951.49625	2950.500387	-2.53668	2.6998	7.42024895984882	0.4364	31	0.93501	N.TDAESQVVAVLEALAGDGEIDPSVPVAAAR.Q
7	171213_PT_H37Rv_1-1.sqt	37136	2	True	2683.276659	2682.289332	-5.97143	3.2781	5.7603528261446	0.4105	15	0.93915	R.RYFNTDAESQVVAVLEALAGDGEID.P
8	171213_PT_H37Rv_1-1.sqt	38033	3	True	3111.466892	3110.484069	-6.59721	3.1188	7.59293428989291	0.4028	26	0.93788	E.GVASYLPDIDPEETSEWLESFDTLLQR.C
9	171213_PT_H37Rv_1-1.sqt	16058	3	True	2172.017831	2172.020367	-1.16758	2.7533	6.4377516497364	0.4052	17	0.90881	R.QYRIDDVAAAPEQTTDPGPGA.-

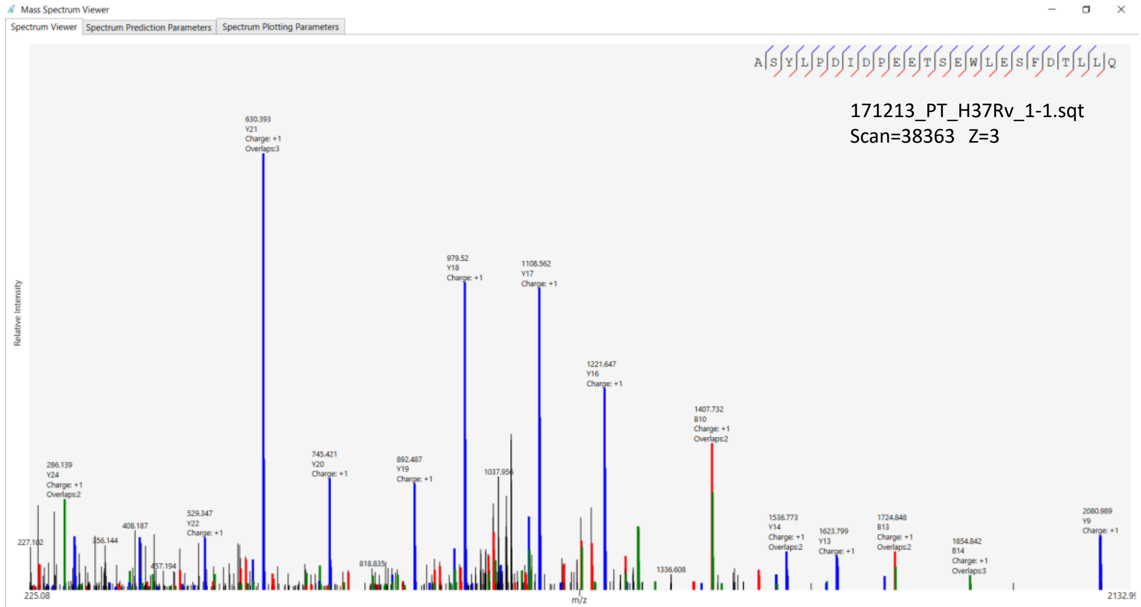

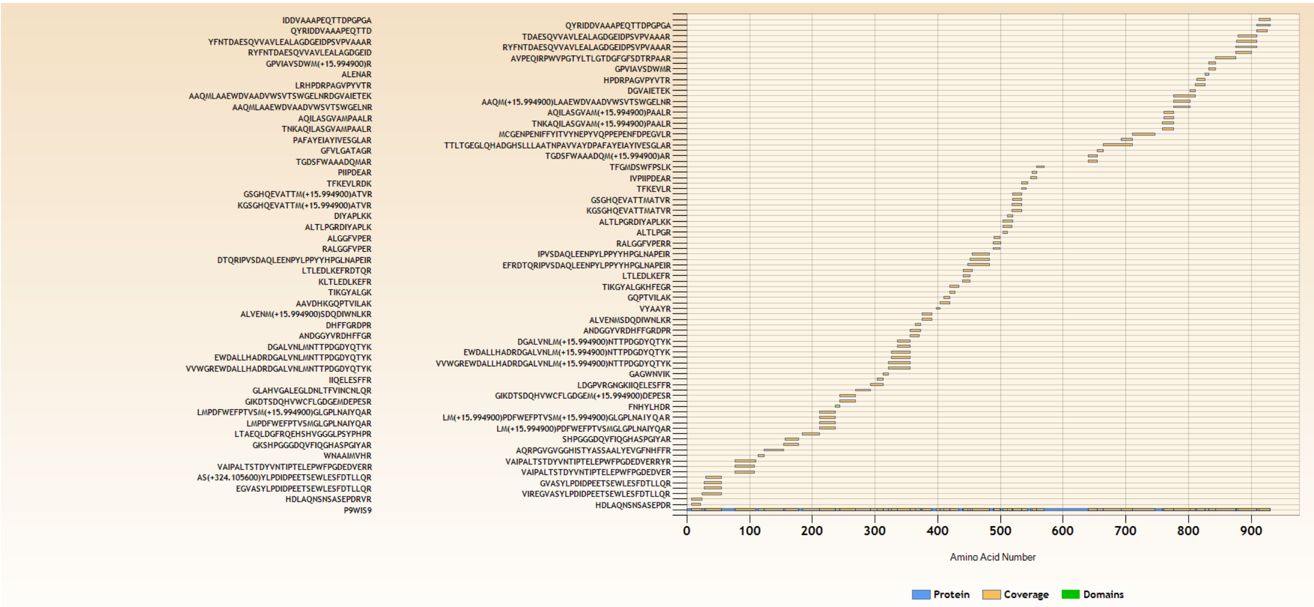

Fatty-acid-AMP ligase FadD28 (Rv2941)
Modification: Hex

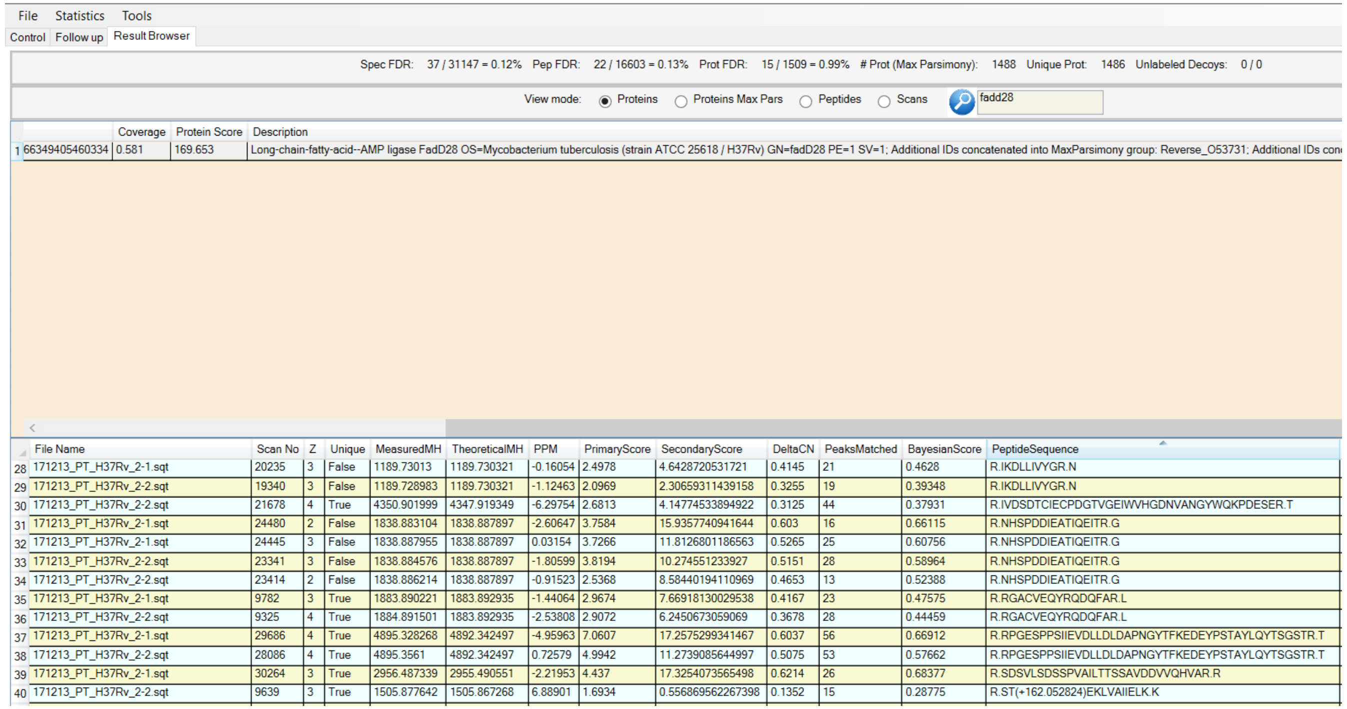

		Coverage	Protein Score	Description
1	66349405460334	0.581	169.653	Long-chain-fatty-acid--AMP ligase FadD28 OS=Mycobacterium tuberculosis (strain ATCC 25618 / H37Rv) GN=fadD28 PE=1 SV=1; Additional IDs concatenated into MaxParsimony group: Reverse_O53731; Additional IDs con

	File Name	Scan No	Z	Unique	MeasuredMH	TheoreticalMH	PPM	PrimaryScore	SecondaryScore	DeltaCN	PeaksMatched	BayesianScore	PeptideSequence
28	171213_PT_H37Rv_2-1.sqt	20235	3	False	1189.73013	1189.730321	-0.16054	2.4978	4.6428720531721	0.4145	21	0.4628	R.IKDLLIVYGR.N
29	171213_PT_H37Rv_2-2.sqt	19340	3	False	1189.728983	1189.730321	-1.12463	2.0969	2.30659311439158	0.3255	19	0.39348	R.IKDLLIVYGR.N
30	171213_PT_H37Rv_2-2.sqt	21678	4	True	4350.901999	4347.919349	-6.29754	2.6813	4.14774533894922	0.3125	44	0.37931	R.IVDSDTCIECPDGTVGEIWVHGDNVANGYWQKPDESER.T
31	171213_PT_H37Rv_2-1.sqt	24480	2	False	1838.883104	1838.887897	-2.60647	3.7584	15.9357740941644	0.603	16	0.66115	R.NHSPDDIEATIQEITR.G
32	171213_PT_H37Rv_2-1.sqt	24445	3	False	1838.887955	1838.887897	0.03154	3.7266	11.8126801186563	0.5265	25	0.60756	R.NHSPDDIEATIQEITR.G
33	171213_PT_H37Rv_2-2.sqt	23341	3	False	1838.884576	1838.887897	-1.80599	3.8194	10.274551233927	0.5151	28	0.58964	R.NHSPDDIEATIQEITR.G
34	171213_PT_H37Rv_2-2.sqt	23414	2	False	1838.886214	1838.887897	-0.91523	2.5368	8.58440194110969	0.4653	13	0.52388	R.NHSPDDIEATIQEITR.G
35	171213_PT_H37Rv_2-1.sqt	9782	3	True	1883.890221	1883.892935	-1.44064	2.9674	7.66918130029538	0.4167	23	0.47575	R.RGACVEQYRQDQFAR.L
36	171213_PT_H37Rv_2-2.sqt	9325	4	True	1884.891501	1883.892935	-2.53808	2.9072	6.2450673059069	0.3678	28	0.44459	R.RGACVEQYRQDQFAR.L
37	171213_PT_H37Rv_2-1.sqt	29686	4	True	4895.328268	4892.342497	-4.95963	7.0607	17.2575299341467	0.6037	56	0.66912	R.RPGESPPSIIEVDLLDLDAPNGYTFKEDEYPSTAYLQYTSGSTR.T
38	171213_PT_H37Rv_2-2.sqt	28086	4	True	4895.3561	4892.342497	0.72579	4.9942	11.2739085644997	0.5075	53	0.57662	R.RPGESPPSIIEVDLLDLDAPNGYTFKEDEYPSTAYLQYTSGSTR.T
39	171213_PT_H37Rv_2-1.sqt	30264	3	True	2956.487339	2955.490551	-2.21953	4.437	17.3254073565498	0.6214	26	0.68377	R.SDSVLSDSSPVAILTTSSAVDDVVQHVAR.R
40	171213_PT_H37Rv_2-2.sqt	9639	3	True	1505.877642	1505.867268	6.88901	1.6934	0.556869562267398	0.1352	15	0.28775	R.ST(+162.052824)EKLVAIIELK.K

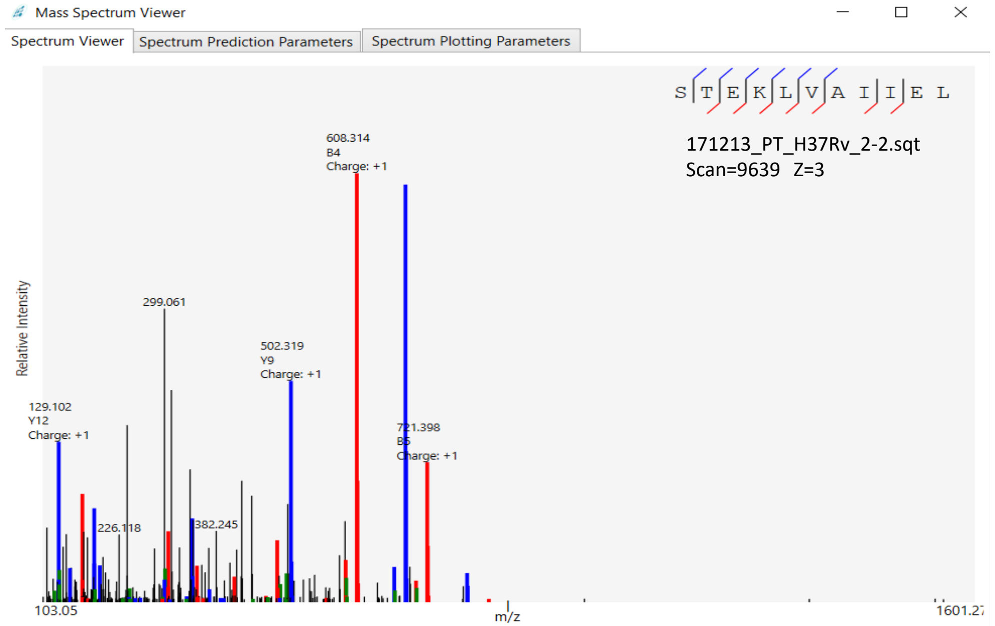

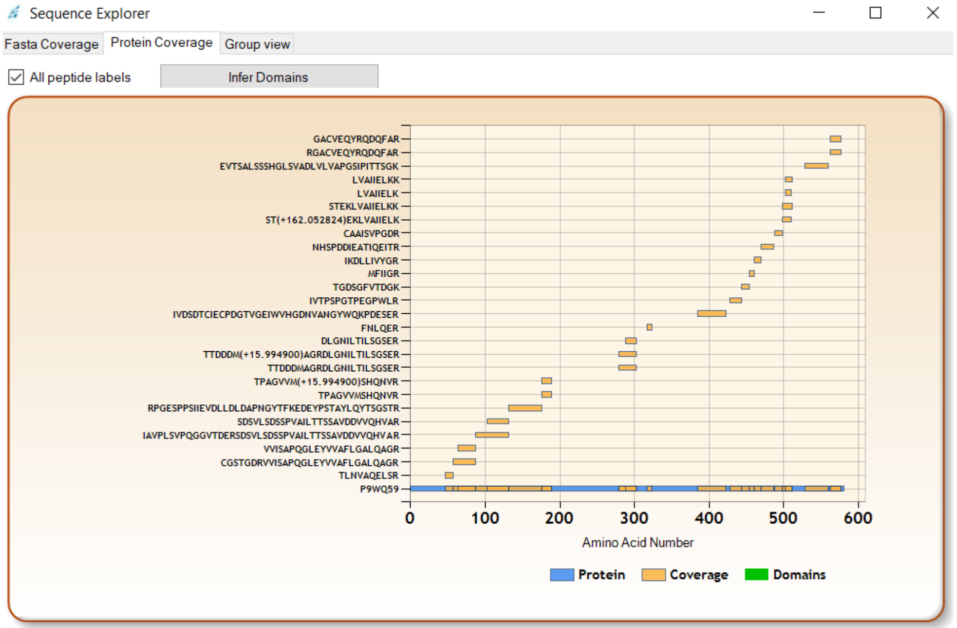

Unknown protein (Rv3491)
Modification: Hex

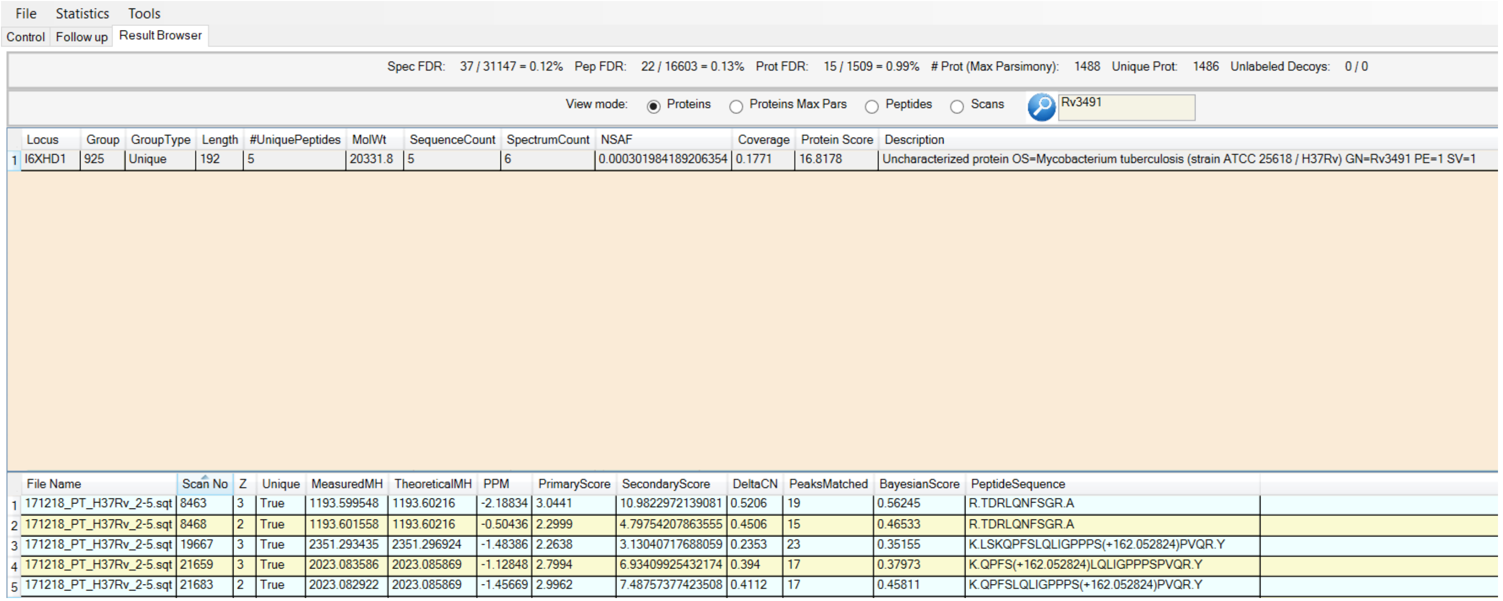

	Locus	Group	GroupType	Length	#UniquePeptides	MolWt	SequenceCount	SpectrumCount	NSAF	Coverage	Protein Score	Description
1	I6XHD1	925	Unique	192	5	20331.8	5	6	0.000301984189206354	0.1771	16.8178	Uncharacterized protein OS=Mycobacterium tuberculosis (strain ATCC 25618 / H37Rv) GN=Rv3491 PE=1 SV=1

	File Name	Scan No	Z	Unique	MeasuredMH	TheoreticalMH	PPM	PrimaryScore	SecondaryScore	DeltaCN	PeaksMatched	BayesianScore	PeptideSequence
1	171218_PT_H37Rv_2-5.sqt	8463	3	True	1193.599548	1193.60216	-2.18834	3.0441	10.9822972139081	0.5206	19	0.56245	R.TDRLQNFSGR.A
2	171218_PT_H37Rv_2-5.sqt	8468	2	True	1193.601558	1193.60216	-0.50436	2.2999	4.79754207863555	0.4506	15	0.46533	R.TDRLQNFSGR.A
3	171218_PT_H37Rv_2-5.sqt	19667	3	True	2351.293435	2351.296924	-1.48386	2.2638	3.13040717688059	0.2353	23	0.35155	K.LSKQPFSLQLIGPPPS(+162.052824)PVQR.Y
4	171218_PT_H37Rv_2-5.sqt	21659	3	True	2023.083586	2023.085869	-1.12848	2.7994	6.93409925432174	0.394	17	0.37973	K.QPFS(+162.052824)LQLIGPPPSPVQR.Y
5	171218_PT_H37Rv_2-5.sqt	21683	2	True	2023.082922	2023.085869	-1.45669	2.9962	7.48757377423508	0.4112	17	0.45811	K.QPFSLQLIGPPPS(+162.052824)PVQR.Y

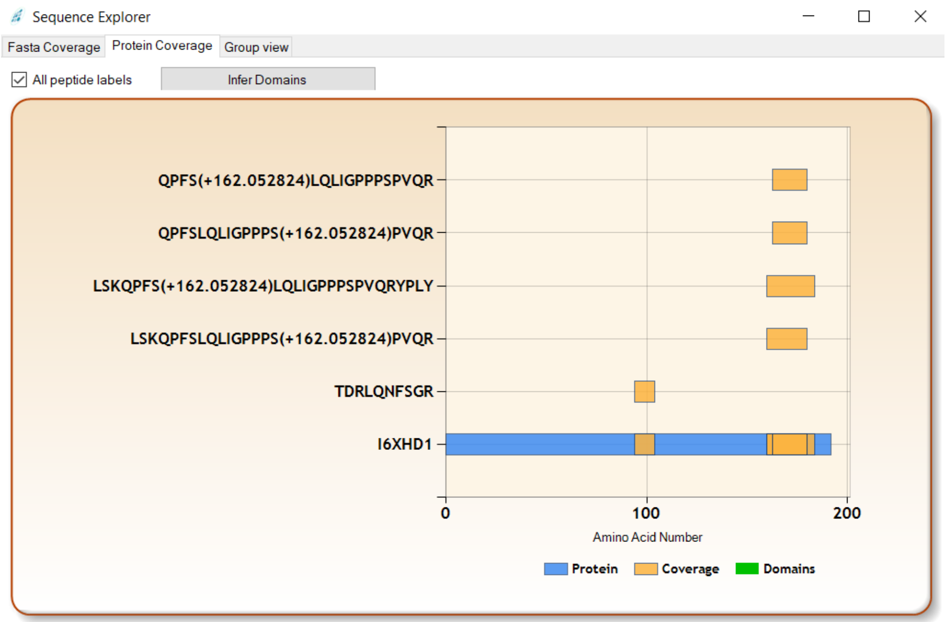

MS/MS Fragmentation of **LSKQPFSLQLIGPPPSPVQR**
Found in **WP_015303914.1** in **NCBIprot**, hypothetical protein [Mycobacterium canettii]

Match to Query 3: 2350.286093 from(2351.293369,1+) index(1)
Data file Scans Rv3491 Hex Mascot.txt

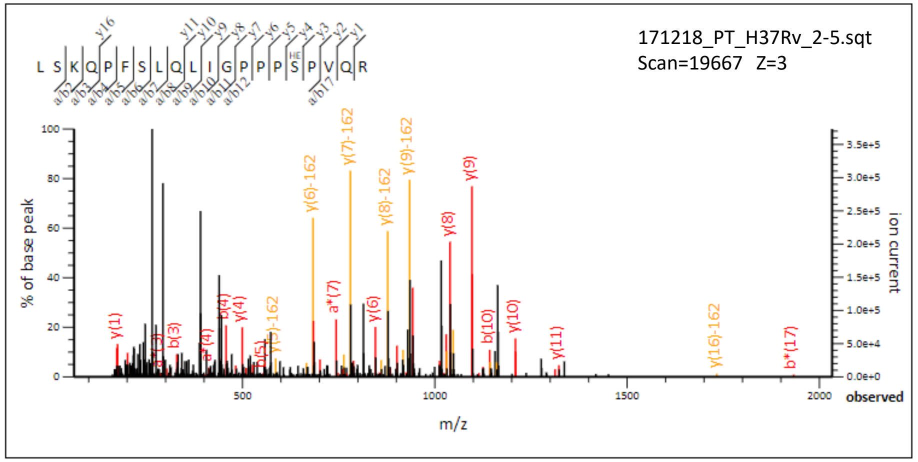

MS/MS Fragmentation of **QPFSLQLIGPPPSPVQR**
Found in **WP_015303914.1** in **NCBIprot**, hypothetical protein [Mycobacterium canettii]

Match to Query 2: 2022.076316 from(2023.083592,1+) index(2)
Data file Scans Rv3491 Hex Mascot.txt

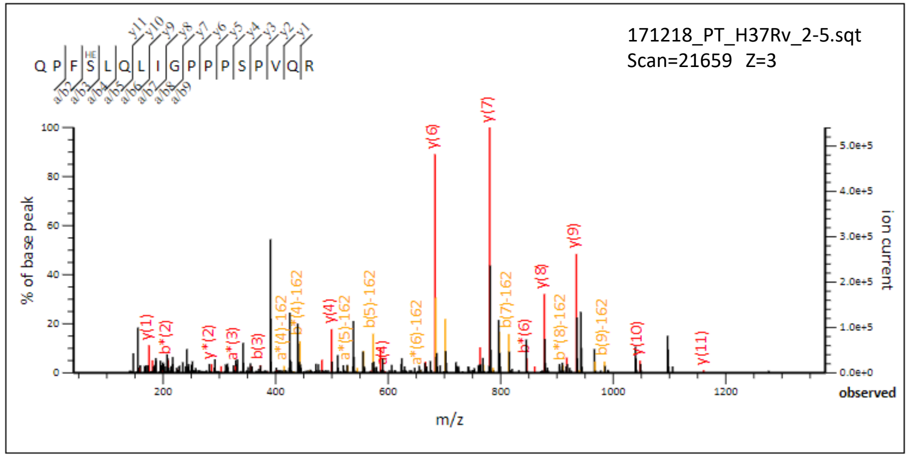

Conserved protein (Rv1352)
Modification: Hex-Hex

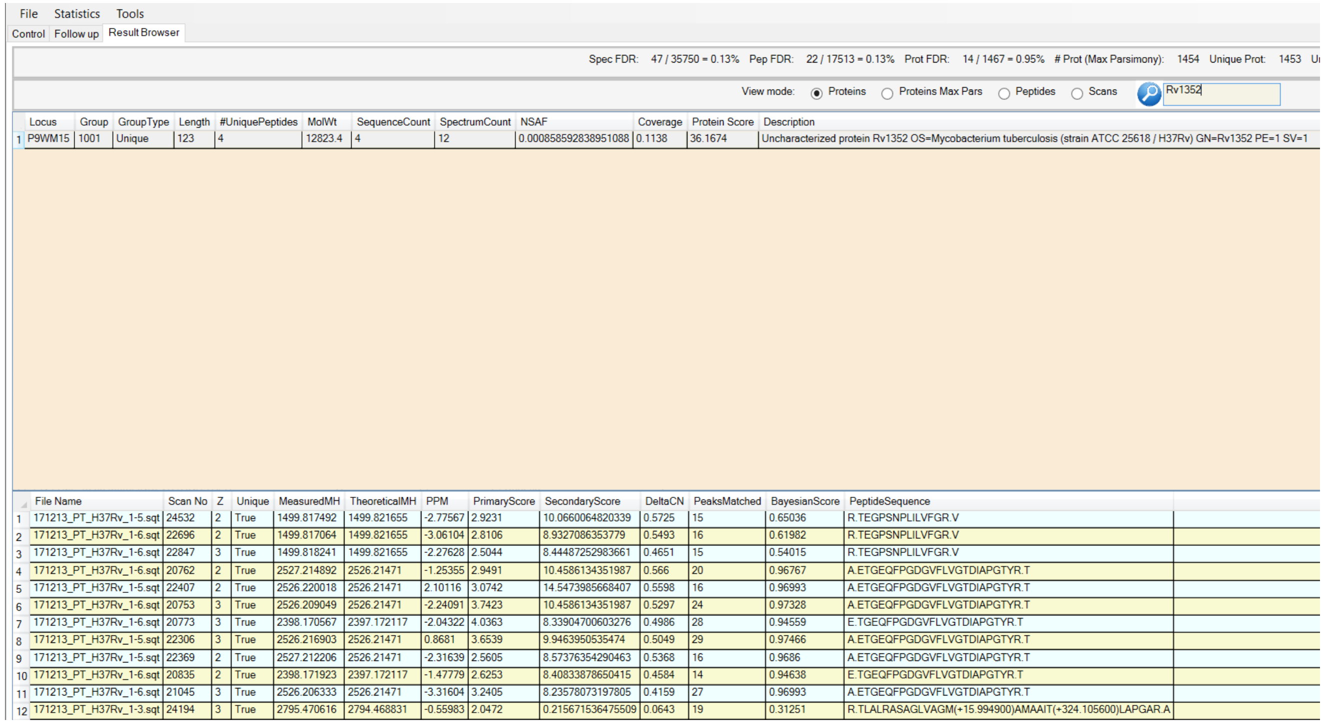

	Locus	Group	GroupType	Length	#UniquePeptides	MolWt	SequenceCount	SpectrumCount	NSAF	Coverage	Protein Score	Description
1	P9WM15	1001	Unique	123	4	12823.4	4	12	0.000858592838951088	0.1138	36.1674	Uncharacterized protein Rv1352 OS=Mycobacterium tuberculosis (strain ATCC 25618 / H37Rv) GN=Rv1352 PE=1 SV=1

	File Name	Scan No	Z	Unique	MeasuredMH	TheoreticalMH	PPM	PrimaryScore	SecondaryScore	DeltaCN	PeaksMatched	BayesianScore	PeptideSequence
1	171213_PT_H37Rv_1-5.sqt	24532	2	True	1499.817492	1499.821655	-2.77567	2.9231	10.0660064820339	0.5725	15	0.65036	R.TEGPSNPLILVFGR.V
2	171213_PT_H37Rv_1-6.sqt	22696	2	True	1499.817064	1499.821655	-3.06104	2.8106	8.9327086353779	0.5493	16	0.61982	R.TEGPSNPLILVFGR.V
3	171213_PT_H37Rv_1-6.sqt	22847	3	True	1499.818241	1499.821655	-2.27628	2.5044	8.44487252983661	0.4651	15	0.54015	R.TEGPSNPLILVFGR.V
4	171213_PT_H37Rv_1-6.sqt	20762	2	True	2527.214892	2526.21471	-1.25355	2.9491	10.4586134351987	0.566	20	0.96767	A.ETGEQFPGDGVFLVGTDIAPGTYR.T
5	171213_PT_H37Rv_1-5.sqt	22407	2	True	2526.220018	2526.21471	2.10116	3.0742	14.5473985668407	0.5598	16	0.96993	A.ETGEQFPGDGVFLVGTDIAPGTYR.T
6	171213_PT_H37Rv_1-6.sqt	20753	3	True	2526.209049	2526.21471	-2.24091	3.7423	10.4586134351987	0.5297	24	0.97328	A.ETGEQFPGDGVFLVGTDIAPGTYR.T
7	171213_PT_H37Rv_1-6.sqt	20773	3	True	2398.170567	2397.172117	-2.04322	4.0363	8.33904700603276	0.4986	28	0.94559	E.TGEQFPGDGVFLVGTDIAPGTYR.T
8	171213_PT_H37Rv_1-5.sqt	22306	3	True	2526.216903	2526.21471	0.8681	3.6539	9.9463950535474	0.5049	29	0.97466	A.ETGEQFPGDGVFLVGTDIAPGTYR.T
9	171213_PT_H37Rv_1-5.sqt	22369	2	True	2527.212206	2526.21471	-2.31639	2.5605	8.57376354290463	0.5368	16	0.9686	A.ETGEQFPGDGVFLVGTDIAPGTYR.T
10	171213_PT_H37Rv_1-6.sqt	20835	2	True	2398.171923	2397.172117	-1.47779	2.6253	8.40833878650415	0.4584	14	0.94638	E.TGEQFPGDGVFLVGTDIAPGTYR.T
11	171213_PT_H37Rv_1-6.sqt	21045	3	True	2526.206333	2526.21471	-3.31604	3.2405	8.23578073197805	0.4159	27	0.96993	A.ETGEQFPGDGVFLVGTDIAPGTYR.T
12	171213_PT_H37Rv_1-3.sqt	24194	3	True	2795.470616	2794.468831	-0.55983	2.0472	0.215671536475509	0.0643	19	0.31251	R.TLALRASAGLVAGM(+15.994900)AMAAIT(+324.105600)LAPGAR.A

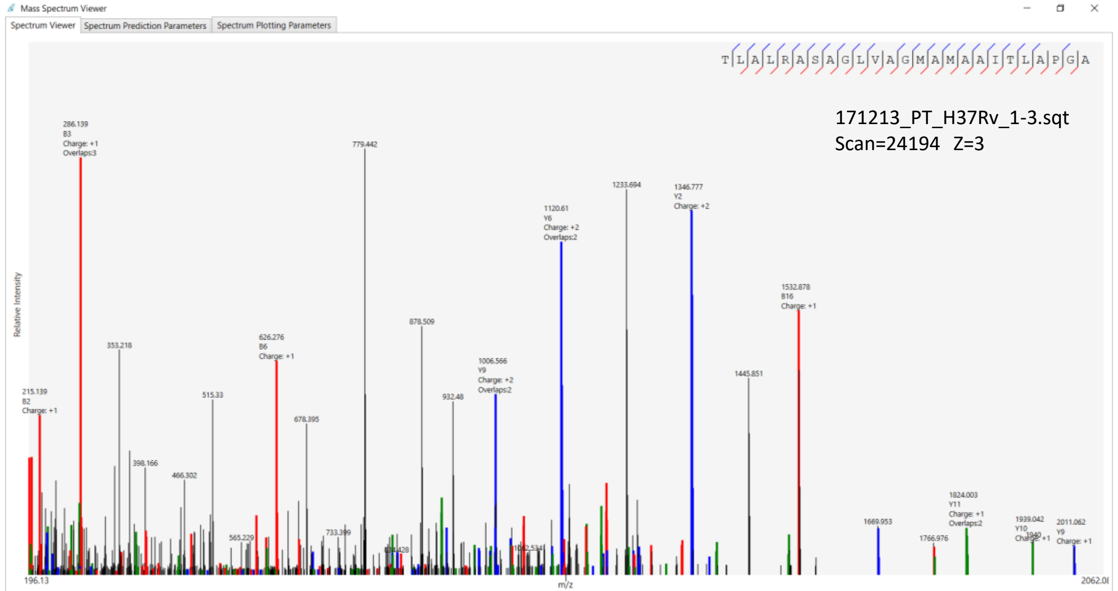

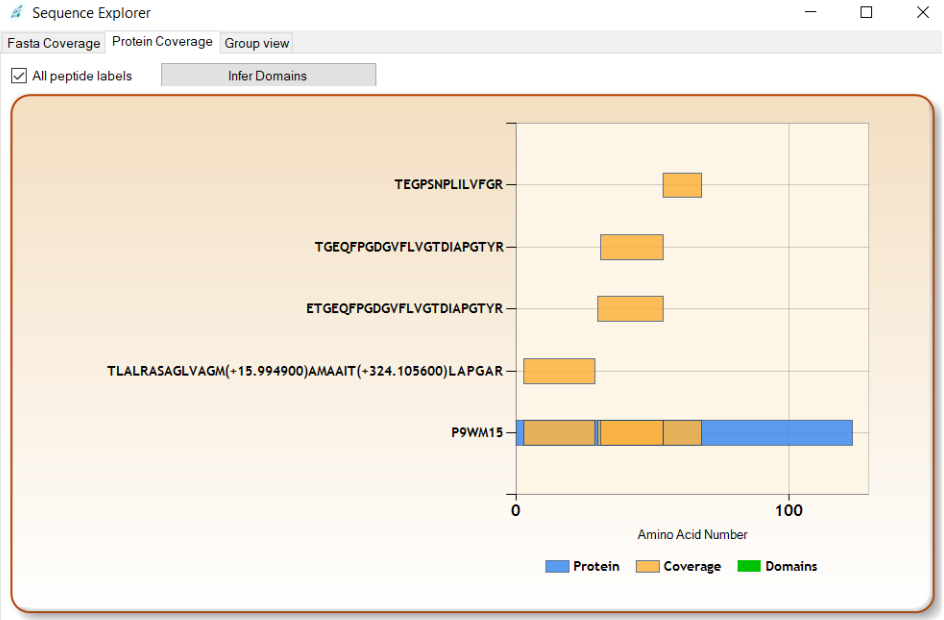

Probable carbamoyl-phosphate synthase large chain CarB (Rv1384)
Modification: Hex-Hex-Hex

File Statistics Tools

Control | Follow up | Result Browser

Spec FDR: 49 / 36000 = 0.14% Pep FDR: 24 / 17635 = 0.14% Prot FDR: 15 / 1505 = 1% # Prot (Max Parsimony): 1487 Unique Prot: 1482 Unlabeled Decoys: 0 / 0

View mode: Proteins | Proteins Max Pars | Peptides | Scans — P9WPK3

	Locus	Group	GroupType	Length	#UniquePeptides	MolWt	SequenceCount	SpectrumCount	NSAF	Coverage	Protein Score	Description
1	P9WPK3	902	Unique	1115	10	118868.8	10	14	0.000107626517146687	0.1695	32.3775	Carbamoyl-phosphate synthase large chain OS=Mycobacterium tuberculosis (strain ATCC 25618 / H37Rv) GN=carB PE=1 SV=1

	File Name	Scan No	Z	Unique	MeasuredMH	TheoreticalMH	PPM	PrimaryScore	SecondaryScore	DeltaCN	PeaksMatched	BayesianScore	PeptideSequence
1	171213_PT_H37Rv_1-1.sqt	32499	3	True	3685.94199	3684.948319	-2.62592	4.8518	11.6778001081605	0.539	32	0.58967	R.LADAGVPIVGTPPEAIDLAEDRGAFGDLLSAAGLPAPK.Y
2	171213_PT_H37Rv_1-1.sqt	27698	2	True	2002.94371	2002.950497	-3.38851	2.7751	6.66873837851164	0.4982	19	0.52184	R.AGFWTAPDPDGGIEEALTR.L
3	171213_PT_H37Rv_1-1.sqt	13664	3	True	1561.833225	1561.833282	-0.0365	2.6406	5.94077143279246	0.4122	22	0.4849	R.ATQLSPEHPVLVDR.F
4	171213_PT_H37Rv_1-1.sqt	36507	3	True	3132.708592	3130.699422	0.78846	2.6471	3.34670919637809	0.325	29	0.44915	R.GNKIDALLATLGGQTALNTAVALYESGVLEK.Y
5	171213_PT_H37Rv_1-1.sqt	36494	4	True	3132.710104	3130.699422	1.2711	2.6701	1.72036947314138	0.1999	35	0.40734	R.GNKIDALLATLGGQTALNTAVALYESGVLEK.Y
6	171213_PT_H37Rv_1-1.sqt	25169	3	True	2189.211704	2188.212466	-1.8783	2.4102	1.95899538860397	0.2309	20	0.38849	R.IAEEIGYPVLVRPSYVLGGR.G
7	171213_PT_H37Rv_1-1.sqt	36617	4	True	3130.704733	3130.699422	1.69642	2.3481	0.142716302201595	0.1241	37	0.38255	R.GNKIDALLATLGGQTALNTAVALYESGVLEK.Y
8	171213_PT_H37Rv_1-4.sqt	30194	4	True	3630.891175	3628.875167	2.56356	2.6021	0.944175935363691	0.1424	42	0.36121	R.IDGYEIRSAAVAGNIPCITTVQGASAAVQGIEAGIR.G
9	171213_PT_H37Rv_1-2.sqt	18460	3	True	2454.182163	2453.196258	-7.10827	2.0717	0.539568092631645	0.1547	22	0.34467	R.NFVEALGKVM(+15.994900)RSLETT(+486.158400)R.A
10	171213_PT_H37Rv_1-3.sqt	18252	3	True	2454.186375	2453.196258	-5.39201	1.7961	0.0954101848046582	0.022	21	0.34178	R.NFVEALGKVM(+15.994900)RSLETT(+486.158400)R.A

Mass Spectrum Viewer

Spectrum Viewer | Spectrum Prediction Parameters | Spectrum Plotting Parameters

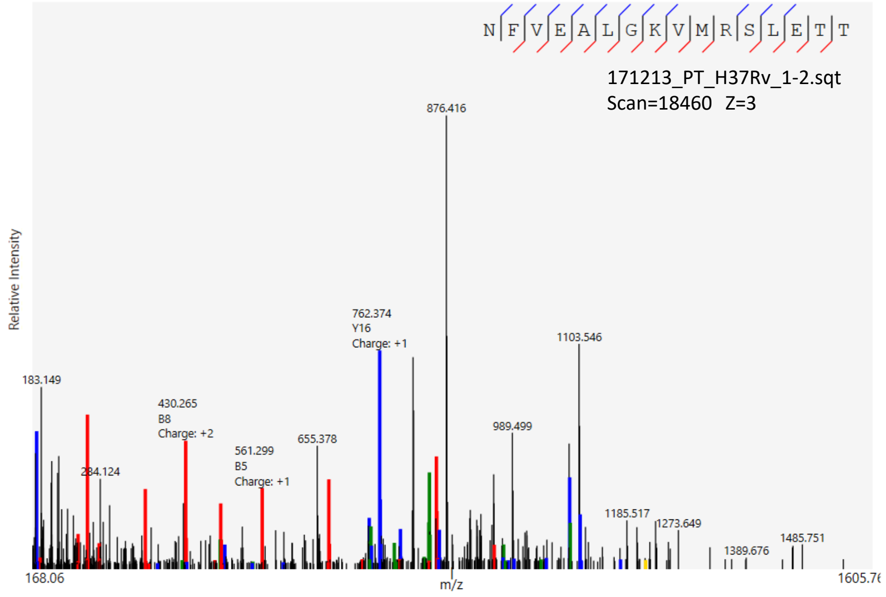

Sequence Explorer

Fasta Coverage | Protein Coverage | Group view

All peptide labels | Infer Domains

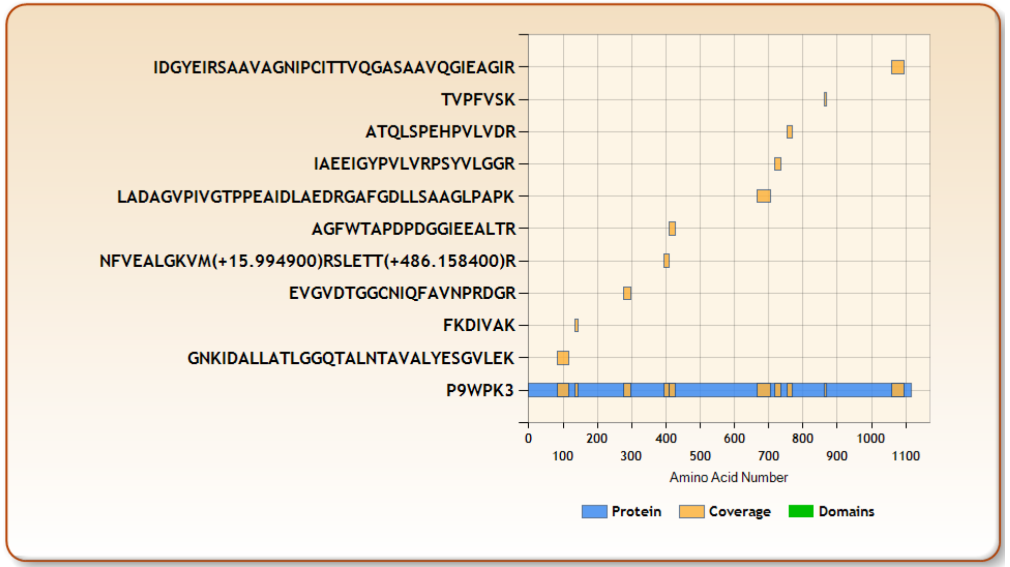

Supplement: S5 Fig — Some examples are shown: 1) DsbF (modification Hex-Hex), 2) LppO (modification: Hex), 3) PstS1 (modification Hex), 4) FhaA (modifcation Hex), 5) DevR (modification Hex-Hex), 6) DnaK (modification Hex-Hex-Hex), 7) Rv3273 (modification Hex-Hex-Hex), 8) Icd2 (modification Hex-Hex-Hex), 9) EsxC (modification Hex), 10) SahH (modification Hex), 11) DeaD (modification Hex), 12 Pks5 (modification Hex-Hex-Hex), 13) Wag31 (modification Hex), 14) GlnA1 (modification Hex), 15) AceE (modification Hex-Hex), 16) FadD28 (modification Hex), 17) Rv3491 (modification Hex), 18) Rv1352 (modification Hex-Hex), 19) CarB (modification Hex-Hex-Hex). (PDF) [file pone.0221837.s006.pdf]
