# Supplementary material for: Integrative proteomic and glycoproteomic profiling of Mycobacterium tuberculosis culture filtrate
Source: PLoS One. 2020 Mar 3;15(3):e0221837. doi: 10.1371/journal.pone.0221837 (PMC7053730; doi:10.1371/journal.pone.0221837)
Supplement: S4 Table — Comparison of our proteomic data against the proteomic quantitative approach performed by de Souza et al, 2011 [9]. (DOCX) [file pone.0221837.s010.docx]

**Table S4.** **Protein abundance comparison between this study and de Souza et al, 2011 (1)**

| CFP TB | CFP* | MPF* | WCL* | N | NSAF_tot | NSAF % | emPAI_tot* | emPAI %* |
| --- | --- | --- | --- | --- | --- | --- | --- | --- |
| ✓ | ✓ | ✓ | ✓ | 299 | 0.784 | 43.2 | 78.38 | 29.2 |
| ✓ | ✓ | ✓ |  | 18 | 0.019 | 1.0 | 49.68 | 18.5 |
| ✓ | ✓ |  | ✓ | 66 | 0.074 | 4.1 | 17.71 | 6.6 |
| ✓ | ✓ |  |  | 18 | 0.012 | 0.6 | 1.15 | 0.4 |
| ✓ |  | ✓ | ✓ | 499 | 0.582 | 32.1 | 76.76 | 28.6 |
| ✓ |  | ✓ |  | 45 | 0.030 | 1.7 | 1.24 | 0.5 |
| ✓ |  |  | ✓ | 245 | 0.169 | 9.3 | 5.03 | 1.9 |
| ✓ |  |  |  | 124 | 0.144 | 8.0 | - | - |
|  | ✓ | ✓ | ✓ | 19 | - | - | 1.53 | 0.6 |
|  | ✓ | ✓ |  | 11 | - | - | 0.29 | 0.1 |
|  | ✓ |  | ✓ | 6 | - | - | 0.60 | 0.2 |
|  | ✓ |  |  | 21 | - | - | 0.35 | 0.1 |
|  |  | ✓ | ✓ | 364 | - | - | 26.92 | 10.0 |
|  |  | ✓ |  | 186 | - | - | 5.49 | 2.0 |
|  |  |  | ✓ | 371 | - | - | 3.53 | 1.3 |

* Data obtained from de Souza *et al*, 2011 (1)


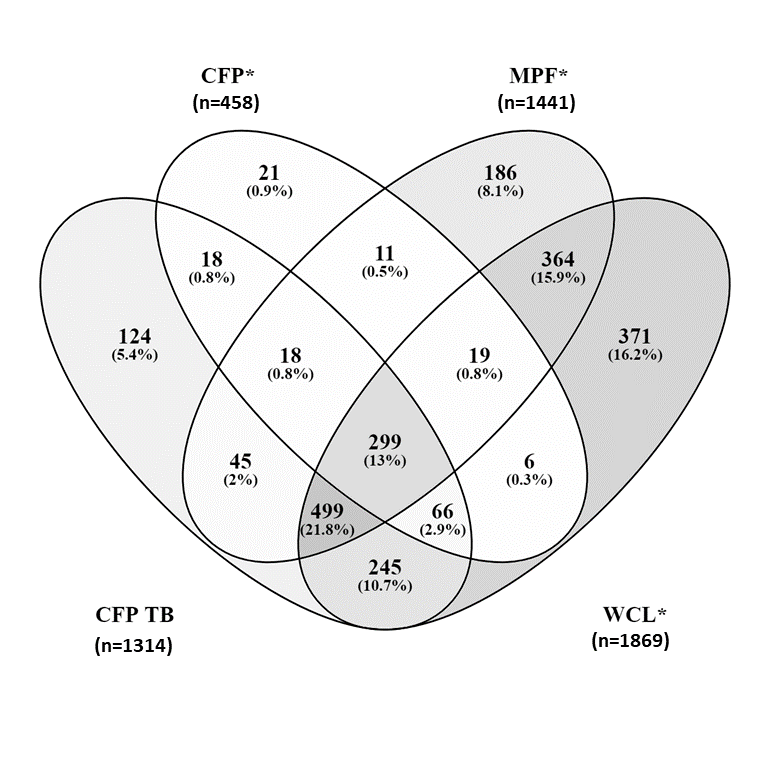


CFP TB: Culture filtrate proteins identified in this study, CFP*: Culture filtrate proteins identified in (1), MPF: Membrane protein fraction identified in (1), WCL: Whole cell lysate identified in (1), N: Number of proteins in each group. NSAF_tot: Sum of NSAF of proteins in each group, emPAI_tot: Sum of emPAI of proteins in each group, as defined in (1).

**Reference**

1. de Souza GA, Leversen NA, Malen H, Wiker HG. Bacterial proteins with cleaved or uncleaved signal peptides of the general secretory pathway. J Proteomics [Internet]. 2011;75(2):502–10. Available from: http://www.ncbi.nlm.nih.gov/entrez/query.fcgi?cmd=Retrieve&db=PubMed&dopt=Citation&list_uids=21920479
